# DREM 2.0: Improved reconstruction of dynamic regulatory networks from time-series expression data

**DOI:** 10.1186/1752-0509-6-104

**Published:** 2012-08-16

**Authors:** Marcel H Schulz, William E Devanny, Anthony Gitter, Shan Zhong, Jason Ernst, Ziv Bar-Joseph

**Affiliations:** 1 Ray and Stephanie Lane Center for Computational Biology, Carnegie Mellon University, 5000 Forbes Avenue, Pittsburgh, PA 15213 USA; 2 Machine Learning Department, Carnegie Mellon University, 5000 Forbes Avenue, Pittsburgh, 15213, PA, USA; 3 Computer Science Department, Carnegie Mellon University, 5000 Forbes Avenue, Pittsburgh, 15213, PA, USA; 4 Department of Biological Chemistry, University of California Los Angeles, Los Angeles, 90095, CA, USA

**Keywords:** Systems biology, Gene regulatory networks, Times series expression data, Dynamic networks, ChIP-chip, ChIP-Seq

## Abstract

**Background:**

Modeling dynamic regulatory networks is a major challenge since much of the protein-DNA interaction data available is static. The Dynamic Regulatory Events Miner (DREM) uses a Hidden Markov Model-based approach to integrate this static interaction data with time series gene expression leading to models that can determine when transcription factors (TFs) activate genes and what genes they regulate. DREM has been used successfully in diverse areas of biological research. However, several issues were not addressed by the original version.

**Results:**

DREM 2.0 is a comprehensive software for reconstructing dynamic regulatory networks that supports interactive graphical or batch mode. With version 2.0 a set of new features that are unique in comparison with other softwares are introduced. First, we provide static interaction data for additional species. Second, DREM 2.0 now accepts continuous binding values and we added a new method to utilize TF expression levels when searching for dynamic models. Third, we added support for discriminative motif discovery, which is particularly powerful for species with limited experimental interaction data. Finally, we improved the visualization to support the new features. Combined, these changes improve the ability of DREM 2.0 to accurately recover dynamic regulatory networks and make it much easier to use it for analyzing such networks in several species with varying degrees of interaction information.

**Conclusions:**

DREM 2.0 provides a unique framework for constructing and visualizing dynamic regulatory networks. DREM 2.0 can be downloaded from: www.sb.cs.cmu.edu/drem.

## Background

Modeling gene regulatory networks (GRNs) is a key challenge when studying development and disease progression. These networks are dynamic with different (overlapping) sets of transcription factors activating genes at different points in time or developmental stages. Reconstructing the dynamics of these networks is a non-trivial task that requires the integration of datasets from different types of genome-wide assays.

Several methods were proposed for reconstructing GRNs (see the following reviews for a general overview: [1-3]). These methods often combine expression and protein-DNA interaction data to recover the underlying networks. However, most methods to date focused on reconstructing static networks and the resulting models did not provide any temporal information. In this paper we focus on the reconstruction of dynamic GRNs using time-series expression data. Such data is prevalent for several species, mostly from microarray studies [4,5] and more recently using RNA-Seq methods [6-8].

While several studies measure time series expression data, the available protein-DNA interaction data is almost always static (either from sequence motifs or from ChIP-chip or ChIP-Seq experiments). This creates a major computational challenge when attempting to integrate these dynamic and static datasets.

Several methods were suggested for clustering time series expression data [9-11], or for constructing dynamic networks with regression-based techniques that rely on only the temporal expression data [12]. While these approaches led to some success, as we show in Results, methods that can utilize both the temporal expression data and the static interaction data can improve upon the expression-only methods.

A number of methods have been suggested for addressing these issues, though most of them were targeted at specific input datasets and did not offer any software to support their general use. For example, [Luscombe et al. 13] created a dynamic network by overlaying TFs regulating differentially expressed genes for different time points. [Lu et al. 14] created a 2D visualization for different dynamic measurements, including time series expression, histone modification, and Pol2-occupancy data using the GATE software [15] although no combined model is presented. Bromberg et al. measure TF activation as a time series and derive pathways that explain activated TFs by integrating subnetworks from PPI networks [16]. Baugh et al. relies on the expression data of transcription factors to identify representatives regulating early development of *C. elegans* embryos [17].

A different way of formulating the problem is to decompose the gene expression data into TF activity and TF affinity values for each expressed gene as suggested by Network Component Analysis [18]. From the matrix of TF affinity values one can construct a dynamic network with connections for each time point [19]. There have been many extensions to this idea with different underlying mathematical models, including ordinary differential equations [20] and Factor analysis [21]. Note however that such regression-based methods do not really take time into account. If one randomly reorders the temporal columns (exchanging, for example the second time point with the fourth etc.) these models will still result in the same network.

One of the first approaches to construct networks that change over time while still incorporating the ordering of time series data was suggested by [Friedman 22] using dynamic Bayesian networks (DBNs). A DBN is a set of directed networks, one for each time point. Although general learning of DBNs is NP-hard there exist conditions where these networks can be learned optimally [23,24]. However, these methods do not scale to hundreds of regulators.

To provide a general method that can be widely applied to reconstructing dynamic regulatory networks, [25] presented DREM, a method that integrates times series and static data using an Input-Output Hidden Markov Model (IOHMM). DREM learns a dynamic GRN by identifying bifurcation points, places in the time series where a group of co-expressed genes begins to diverge. These points are annotated with the TFs controlling the split leading to a combined dynamic model. Since its release 5 years ago the DREM software has been used for modeling a wide range of GRNs for example stress response in yeast [25] and *E. coli*[26], development in fly by the modENCODE consortium [8], stem cell differentiation in mice [27] and disease progression in human [28].

While DREM has been successfully used for multiple species, so far each group using it had to obtain its own protein-DNA interaction data. Since such data is often dispersed among several databases, websites and publications, this step was a major hurdle to using DREM. Other features not supported in the original DREM version included: the integration of motif discovery, the ability to utilize dynamic ChIP binding data [29,30] and TF expression data, and visualization of these new data types. In this paper we discuss a new version of DREM, termed DREM 2.0, that addresses all these limitations. As we show, by addressing these issues DREM 2.0 improves upon both methods that do not integrate static information in the analysis of dynamic data and the previous version of DREM which lacked the above features.

## Implementation

DREM 2.0 is implemented entirely in Java and will work with any operating system supporting Java 1.5 or later. Portions of the interface of DREM 2.0 are implemented using third party libraries, the Java Piccolo toolkit from the University of Maryland [31] and the Batik toolkit for svg export of network images [32]. DREM 2.0 also supports batch mode for automated execution. DREM 2.0 makes use of external Gene Ontology (GO) and gene annotation files. DREM 2.0 downloads these files directly from the GO website [33].

### Time-specific binding of regulators

**Figure 1:**
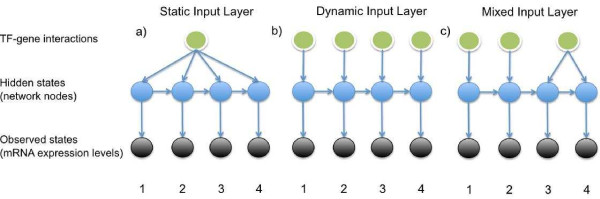
**Possible IOHMM topologies in DREM 2.0.** The basic topology for a DREM 2.0 IOHMM is shown. The hidden states represent the network nodes (in blue) that we are interested in. The observations (black nodes) are the gene expression ratios which are given to the model, these are dynamic and dependent on the time point. The protein-DNA interaction data (green nodes) are used as supervised *input* data to construct the network. (**a**) In the original DREM formulation only one static input node is connected to all hidden nodes. In DREM 2.0 the nodes in the input layer can be dynamic and dependent on the time point with a topology either fully dynamic (**b**) or a mix of static and dynamic input (**c**).

The underlying Input-Output Hidden Markov Model learning can now accommodate dynamic input data for each time point in the following way. The transition probabilities for the IOHMM are derived from a logistic regression classifier that uses the protein-DNA interaction data as supervised input and utilizes them to classify genes into diverging paths at a split node in the model. In the new version the nodes in the input layer can be dynamic and thus the function can depend on input from the specific time point it is associated with. See Figure 1 for an illustration.

## Results

### Using DREM 2.0

**Figure 2:**
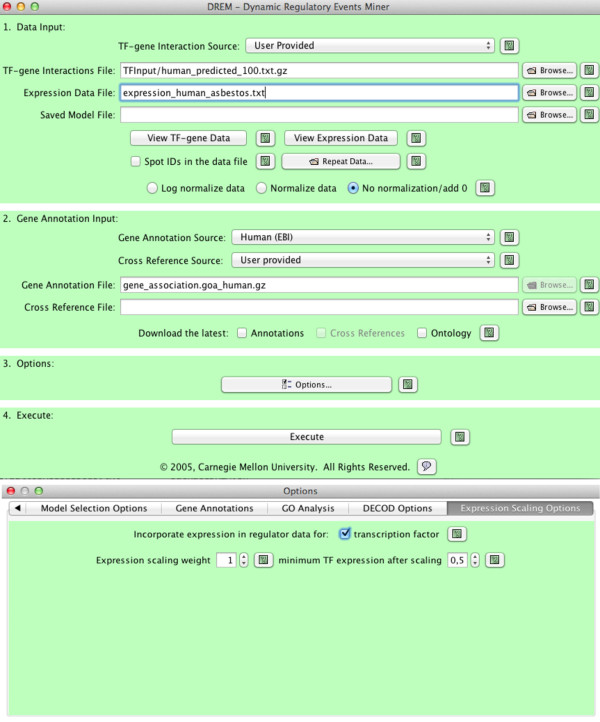
**DREM 2.0 input dialog.** Top: Input dialog for the DREM 2.0 software. Bottom: Selected tab for the Options window, shows the dialog for the activation of TF expression level scaling, see text for details.

**Figure 3:**
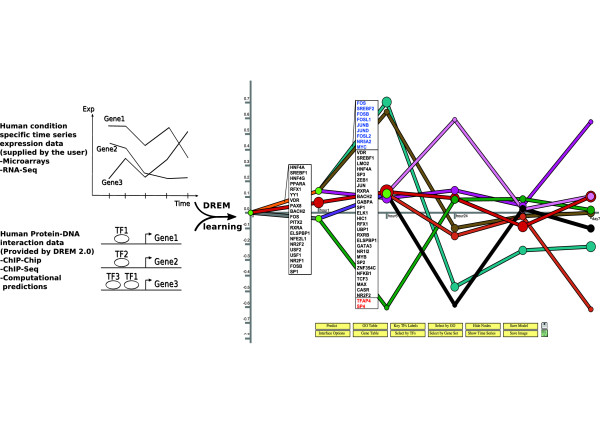
**Analysis of asbestos time series data set.** DREM 2.0 analysis of expression data from human A549 lung cells treated with asbestos using predicted protein-DNA interactions. *(left)* Input data supplied to DREM 2.0. *(right)* The model learned for the 5 time points. TFs (IDs in boxes) are predicted to regulate genes diverging at green split nodes. TFs in blue and red are up- and down-regulated, respectively.

**Figure 4:**
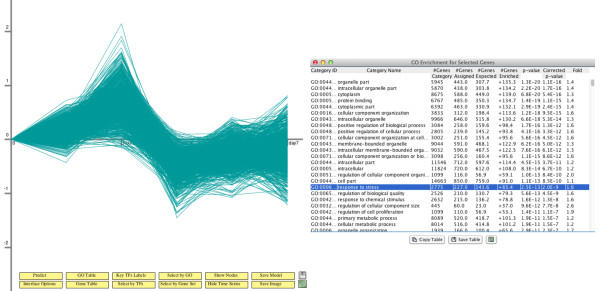
**GO enrichment analysis of DREM paths.** DREM facilitates downstream analysis of the regulatory network. As an example, DREM supports GO term enrichment analysis on paths of the model. *(left)* shows all genes that are assigned to the path with highest expression ratios at the 1 hour time point. *(right)* After clicking a path in the model, a GO enrichment analysis can be performed by DREM for all genes on the path.

**Figure 5:**
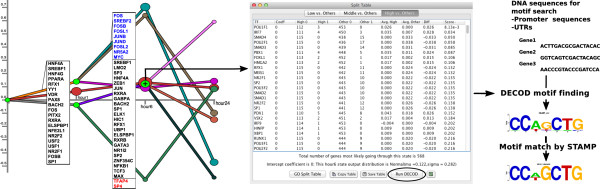
**DECOD motif search in DREM 2.0.***(left)* DECOD motif search was performed for one node (‘+’ sign). *(middle)* After clicking the node, the DREM split table opens which shows the enrichment of TFs on gene sets divided by the split. As this split has three outgoing paths, DECOD can be run in three different ways. Here, we compared genes in the highest path against the other two paths (Tab “High vs. Others”) by clicking the Run DECOD button (circled). *(right)* one of the TF motifs found by DECOD using EPD promoter sequences. Its most similar match in TRANSFAC according to STAMP highly resembles the TF binding motif of HEB/TCF12, see text for details.

Users input their time series expression data by using the graphical user interface (GUI) (see Figure 2). DREM 2.0 can transform the data and combine time point repeats. Next, users select a protein-DNA interaction data set for the species they are working with. DREM 2.0 includes protein-DNA interaction data for several species (see Table 1 for a full list). After selecting the species and interactions the user can set various learning parameters or use the default settings (see Additional file 1). Once the data is entered the user selects the ‘execute’ button which runs DREM 2.0 on the input data and results in the dynamic network learned by DREM 2.0 (for example, the one displayed in Figure 3). DREM 2.0 supports downstream analysis using external databases (for example GO as shown in Figure 4) and software (for example, DECOD and STAMP, as shown in Figure 5, see also below).

**Table 1:** Statistics for protein-DNA datasets supplied with DREM 2.0

**Species**	**#TFs**	**#genes**	**Protein-DNA interactions**	**Type**	**Reference**
*S. cerevisiae*	205	6,230	22,167	ChIP-Chip, conservation	[34,35]
*E.coli*	124	1,763	3,520	curated + computational	[26]
*D. melanogaster*	77	12,504	158,558	ChIP-Chip,ChIP-Seq	[8]
*M. musculus*	336	16,641	468,319	computational prediction,supplement	[36]
*H. sapiens*	127	19,755	954,377	ChIP-Seq	[37]
*H. sapiens*	349	17,848	514,925	computational prediction	[36]
*A. thaliana*	68	8,132	11,354	diverse experimental evidence	[38]

Number of protein-DNA interactions for TFs and target genes for the six supplied species *H. sapiens*, *A. thaliana*, and *M. musculus*, *S. cerevisiae*, and *E.coli* since DREM 2.0. Higher-confidence subsets of these interactions are also provided for some species. More details can be found in Additional file 2.

### DREM 2.0 Analysis of asbestos induction

As a running example to illustrate the new features, we used the human protein-DNA data now available with DREM 2.0 to analyze an expression experiment studying the effects of asbestos on human lung adenocarcinoma cells (A549) [39] (Figure 3). Preprocessing and parameters for the analysis are described below. DREM 2.0 successfully predicts enrichment of TFs known to be relevant in asbestos exposure, *e.g.*, TFs from the FOS family [39], that are shown to be up-regulated at the 6 hour time point (blue IDs Figure 3).

### Parameters and datasets for the asbestos analysis

The time series data for asbestos treatment of human lung cancer cells [39] was downloaded from GEO (record: GSE6013). The dataset contains gene expression data measured with Affymetrix human gene expression arrays 1, 6, 24, 48 hours, and 7 days after asbestos exposure and a control time series without exposure. The array data was normalized with quantile normalization using RMAExpress (version 1.0.5) with default parameters [40].

*Lo*_*g*2_ ratios of exposed versus control were computed as input to DREM 2.0. The human binding predictions (top 100 threshold, see Additional file 2) were used as the regulatory dataset for DREM 2.0. For the DREM 2.0 analysis the following options were not set to default values: (i) genes in the time course were discarded if “Minimum Absolute Expression Change” was smaller than 0.5, (ii) “incorporate expression in regulator data” was activated for transcription factors with “Expression scaling weight” set to 1. For the annotation of split nodes (Figure 3) the “Path significance conditional on Split” enrichment *p*-value in the GUI was set to be ≤ 5·1^0−5^.

For the motif analysis DECOD [41] version 1.01 was downloaded and connected with DREM 2.0 using the GUI interface. 8512 human promoter sequences (-499,+100 bp relative to transcription start site) were downloaded from the EPD promoter database (from the website: Last update 11 Nov. 2009) [42]. DECOD was run to search for motifs of length 7 with the exact mode and STAMP [43] motif similarity search was conducted against TRANSFAC (version 11.3) using default parameters [44]. The reported motif (below) is the 3rd motif found by DECOD with a similarity *E*-value of 3.93e-12 returned by STAMP.

### Supporting additional species

DREM 2.0 utilizes time series expression data (from a specific condition, for example the asbestos data used in this paper) and static interaction data which is often condition-independent (for example, DNA binding motifs). The original version of DREM [25] only provided such static data for *S. cerevisiae*, which meant that users studying other species had to collect their own static data as well as the condition-specific time series data. Over the years we have included protein-DNA interaction data for *E. coli* and human, but several other species were still not supported, limiting DREM’s usage. We have now collected static data for a number of additional species (*M. musculus, D. melanogaster, A. thaliana*) and have added additional high throughput protein-DNA interaction datasets for human as well. With these additions DREM 2.0 now supports most of the well-studied organisms facilitating much wider use of the method. Table 1 lists the current species supported, the number of interactions we have for each species and where these interactions were obtained. More details regarding these datasets can be found in Additional file 2.

### Utilizing the expression levels of TFs

The original version of DREM did not use any information regarding the expression levels of the TFs predicted to regulate split nodes. The underlying reason for this was the fact that many TFs are post-transcriptionally regulated and relying on their expression to determine activity may lead to missing important TFs. In the new version, we still maintain the ability to identify TFs that are only post-transcriptionally regulated. However, we have added a new computational module that allows the method to utilize expression information for those TFs that are transcriptionally regulated. For each TF, its binding prior is elevated based on the TF’s expression level using a logistic function. Thus, active TFs have a stronger prior of being selected as regulators by DREM 2.0 (see Additional file 2). We have also changed the visualization in DREM 2.0 to highlight such factors. In Figure 3, which is a screenshot from DREM 2.0, active TFs are highlighted in blue and repressed TFs in red.

### Finding DNA motifs at split nodes with DECOD

During learning DREM assigns genes to paths in the network model and uses split nodes (light green nodes in Figure 3) to represent sets of genes that change their expression between consecutive time points. TFs are assigned to split nodes allowing DREM to infer their time of activation. When the protein-DNA interaction data is unable to explain some of the split nodes (i.e. no TF is assigned to that split), it could mean that the interaction data is incomplete. To still allow the identification of such TFs, we integrated with DREM 2.0 the discriminative motif finder DECOD [41]. The user can search for discriminative DNA motifs between DNA, *e.g.* promoter, sequences of genes assigned to diverging paths emerging out of any split node. The method uses two sets (genes going up and down from the split) to discriminatively search for motifs. The predicted DNA motifs can be matched against known motif databases using STAMP [43]. To highlight the utility of this new feature in DREM 2.0 we used it on the asbestos data described above. As can be seen, not all split nodes had been assigned in Figure 3. We have thus used the new DECOD feature to identify TFs for one of these splits (‘+’ sign in Figure 5). A database motif search with STAMP reveals a motif with significant similarity to HEB/TCF12. TCF12 was indeed missing among significant TFs in the split table (Figure 5, middle), perhaps because of incomplete data. However, a DNA inversion close to the *TCF12* gene was recently found in lung cancer patients [45] indicating that this protein may be playing a role in regulating gene response in the lung.

In order to test the ability of DECOD to recover TF binding motifs at DREM split nodes for the case where no TF-gene interaction data is available, we have conducted the following analysis. A DREM model using the asbestos expression data was built without using the TF-gene interaction data. Then, EPD promoter sequences for genes at the 6 hour split node where used for motif search with DECOD. We searched for motifs of length 6-8 and selected all those with significant matches in TRANSFAC (using the STAMP motif comparison tool). After grouping TFs from the same family, 10 of the 24 TFs identified in the original run of DREM for this split were found in the DECOD derived set (see Additional file 2 for details).

### Supporting continuous and dynamic binding data

The original version of DREM only supported three binding states (activator/ repressor/ no regulation) interaction data. DREM 2.0 now supports continuous binding values. These can be derived from *p*-values of ChIP-Seq calling procedures or from computational affinity predictions [46]. Thus, in the new version the same regulator may have a different binding value for each gene. The classifier weighs a target with a large binding value higher than targets with a lower binding value. A plausible way to turn ChIP binding *p*-values into DREM 2.0 binding values is to set b=−logp-value. These continuous binding values can then be passed to DREM 2.0.

In addition, DREM 2.0 also supports temporal binding data. While most interaction data is still static, dynamic binding data is becoming available. Recent studies have shown that TFs may alter their binding behavior depending on the time point [29,30] necessitating methods that can utilize such information when available. In its original implementation DREM could only use static protein-DNA interaction data when learning logistic regression classifiers for the transition probabilities in the IOHMM. We have now revised this allowing the learning algorithm to support dynamically changing protein-DNA interaction data (see Implementation). For each time point an independent data set can be passed to the logistic regression classifier. Since dynamic binding data is often only available for a (small) subset of TFs, DREM 2.0 supports a joint static-dynamic input format for protein-DNA interactions.

The ability to incorporate temporal binding data allows DREM to reduce false positive assignments by only assigning TFs that are active at that time point (based on the time points binding data). This in turn can both help identify co-regulators for which only computational predictions exists and also lead to the identification of different waves of transcriptional regulation, where the same TFs activate different sets of genes at different time points.

### Comparison to previous methods

**Figure 6:**
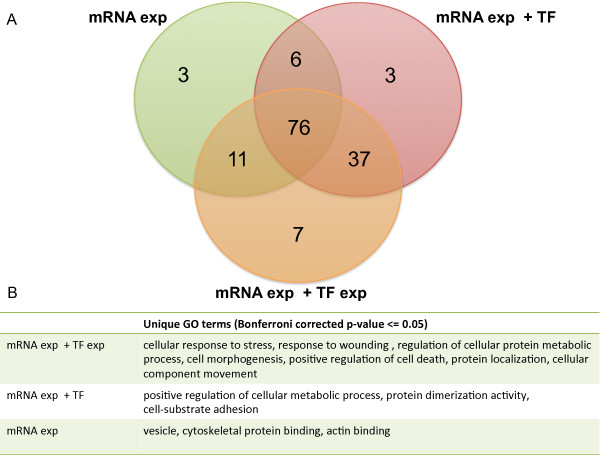
**Comparison of different approaches.** GO analysis of path enrichment in dynamic networks constructed by DREM 2.0 for the asbestos data set. The enrichment of GO terms for all paths, after Bonferroni multiple testing correction, is depicted. Three different learning scenarios are compared: construction without any TF input just using the mRNA expression data (mRNA exp), construction using protein-DNA predicted binding events (mRNA exp+TF), and construction using protein-DNA predicted binding events and the new TF-expression scaling method (mRNA exp+TF exp). **A**) Comparison of the enriched GO terms with corrected p-value below 0.05 for each method shown as a Venn diagram. **B**) Display of GO terms that are uniquely identified by each method. Leveraging the TF-expression level improves the GRN construction in addition to using the protein-DNA interaction data.

We used the asbestos data to compare some of the new features in DREM 2.0 to other methods and to the previous version of DREM. First, to compare DREM 2.0 to methods that only use one type of data (clustering the expression data) we ran DREM 2.0 without using the static protein-DNA interaction information. This is similar to several clustering methods that have been suggested for time series data [9,10]. To compare to the original version of DREM we also reran the asbestos data using TF-DNA interaction data but without using the TF expression information. As a performance metric we used the number of enriched GO terms, a common comparison strategy [11,47]. In Figure 6 the significant GO terms after multiple testing correction are compared for the three methods. Leveraging the TF-expression leads to the highest number of significant GO terms (Figure 6A) and the identification of additional relevant functions that are not identified by the other two variants, including the GO terms *cellular response to stress* and *positive regulation of cell death* (Figure 6B).

## Discussion and conclusions

While several methods can be used to reconstruct GRNs using time series expression data, most such methods either rely only on the expression data itself or result in static networks that do not consider the ordering of the time points. DREM provides not only an alternative to these methods but also a rich GUI and as such, has been used by several groups in multiple species.

Although here we used both treatment and control time series, DREM can also be used with only the treatment time series by taking the log fold change w.r.t. time point 0, see [25] for an example.

The new version eases the application to several species by directly supplying protein-DNA interaction data and incorporating de-novo discriminative motif discovery. In addition we have made other improvements including the ability to utilize and view the expression levels of the TFs and to use dynamic protein-DNA interaction data. Combined, we believe that these improvements will make DREM 2.0 a more widely used software package for the reconstruction of dynamic GRNs.

## Availability and requirements

· **Project name:** DREM

· **Project homepage:**www.sb.cs.cmu.edu/drem

· **Operating system(s):** Platform independent

· **Other requirements:** Java 1.5 or higher

· **License:** Free to academics/non-profit

· **Any restrictions to use by non-academics:** License needed

## Abbreviations

DREM, Dynamic Regulatory Events Miner; TF, Transcription factor; GRN, Gene regulatory network; DBN, Dynamic Bayesian network; ChIP, Chromatin immuno precipitation; IOHMM, Input-output hidden Markov model; GUI, Graphical user interface; GO, Gene Ontology; MGD, Mouse Genome Database; HGNC, HUGO Gene Nomenclature Committee; RNA-Seq, Next generation sequencing of messenger RNAs.

## Competing interests

The authors declare that they have no competing interests.

## Author’s contributions

MHS, WED, AG, SZ designed and implemented the new version. MHS, AG, SZ, JE performed the data collection and analysis. ZBJ supervised the work. MHS and ZBJ wrote the manuscript. All authors read and approved the final manuscript.

## Authors’ information

Marcel H. Schulz and William E. Devanny joint first authors.

## Funding

Work supported in part by NIH grant 1RO1 GM085022.

## Supplementary Material

Additional file 1 **DREM 2.0 Manual.** The Manual for using the DREM 2.0 software with details of all parameters and the different dialogs in the GUI. Click here for file

Additional file 2 **Supplementary Methods.** Additional description for DREM 2.0 for TF expression level scaling, data collection for the protein-DNA binding data sets and the analysis with DECOD on an unannotated split node. Click here for file
