# Supplementary material for: DREM 2.0: Improved reconstruction of dynamic regulatory networks from time-series expression data
Source: BMC Syst Biol. 2012 Aug 16;6:104. doi: 10.1186/1752-0509-6-104 (PMC3464930; doi:10.1186/1752-0509-6-104)
Supplement: Additional file 1 — DREM 2.0 Manual. The Manual for using the DREM 2.0 software with details of all parameters and the different dialogs in the GUI. [file 1752-0509-6-104-S1.pdf]

DREM

Dynamic Regulatory Events Miner (v2.0)

User Manual

Jason Ernst (jernst@cs.cmu.edu)
Anthony Gitter (agitter@cs.cmu.edu)
Marcel H. Schulz (maschulz@cs.cmu.edu)
William E. Devanny
Ziv Bar-Joseph
Machine Learning Department
School of Computer Science
Carnegie Mellon University

Contents

1 Introduction **1**

2 Preliminaries **1**

3 Input Interface **2**

3.1 Data Input . . . 3
3.1.1 Transcription Factor-gene Interactions File . . . 3
3.1.2 Time Specific Binding Data . . . 5
3.1.3 Expression Data File . . . 5
3.1.4 Saved Model File . . . 7
3.2 Gene Annotation Info . . . 7
3.3 Options . . . 9
3.3.1 Filtering Options . . . 9
3.3.2 Search Options . . . 11
3.3.3 Model Selection Options . . . 12
3.3.4 Gene Annotations Options . . . 14
3.3.5 GO Analysis Options . . . 16
3.3.6 DECOD Options . . . 17
3.3.7 Expression Scaling Options . . . 18
3.4 Search Progress Dialog . . . 18

4 DREM Main Output Interface **20**

4.1 Hide/Show Time Series . . . 26
4.2 Hide/Show Nodes . . . 27
4.3 Interface Options . . . 28
4.4 Key TFs Labels . . . 30
4.5 Select by TFs . . . 32
4.6 Select by GO . . . 34
4.7 Select by Gene Set . . . 36
4.8 Predict . . . 38
4.9 Gene Table . . . 40
4.9.1 TF-Summary Table . . . 41
4.10 GO Table . . . 43
4.11 Save Model . . . 45
4.12 Save Image . . . 45
4.13 Path Table . . . 45
4.14 Split Table . . . 47

A Defaults File Format **51**

B TF-gene Interaction Files **53**

C Gene Annotation Sources **54**

1 Introduction

Welcome to DREM!

DREM is an acronym for the Dynamic Regulatory Events Miner, a software program for modeling and analyzing the dynamics of transcriptional gene regulation. DREM takes as input time series gene expression data and input that associates transcription factors with the genes they regulate. This regulatory information could come from Chromatin Immunoprecipitation (ChIP)-chip experiments or transcription factor binding site motif information. The transcription-factor gene regulation input does not need to be associated with specific time points. DREM after executing a computational method described in [7] outputs an annotated dynamic regulatory map based on the data that can be interactively explored. The dynamic regulatory map highlights bifurcation events in the time series, that is places in the time series where sets of genes which previously had roughly similar expression level diverge. Often these bifurcation events can be explained by transcription factors selectively regulation a certain subset of genes. DREM annotates these events with transcription factors potentially responsible for them.

DREM is related to another time series expression analysis software the Short Time-series Expression Miner (STEM) [4]. While STEM focuses on identifying independent significant patterns in short time series data, DREM provides a global map of the gene regulation of the time series. The DREM method also incorporates in transcription factor-gene regulation information. DREM also is not limited to analyzing short time series data as STEM is. Some of the input file formats and options are the same in DREM as in STEM, in particular options related to gene filtering and the Gene Ontology analysis. For these aspects of DREM which are the same as in STEM, the relevant portions of the STEM manual have been incorporated into this manual.

2 Preliminaries

- To use DREM a version of Java 1.5 or later must be installed. If Java 1.5 or later is not currently installed, then it can be downloaded from **http://www.java.com**.
- To execute DREM in Windows with its default initialization options simply double click on the file **drem.cmd** in the **drem** directory. When starting DREM using the **drem.cmd** file, DREM will obtain the default settings from the **defaults.txt** file unless the **drem.cmd** file was modified.
- To execute DREM from a command line change to the **drem** directory type and then type:

  ```
  java -mx1024M -jar drem.jar
  ```

- DREM can be started with its initial settings specified in a default settings file. The format of a default setting file is specified in Appendix A. To have DREM load its initial settings from a default settings file from the command line, append '-d' followed by the name of the default settings file to the above command. For instance to have DREM start with the settings specified in the file **defaults.txt** use the command:

  ```
  java -mx1024M -jar drem.jar -d defaults.txt
  ```

- DREM can be run in batch mode in order to learn models without going through the graphical interface. Batch mode is useful for learning multiple DREM models in parallel or interacting with DREM through external scripts. In batch mode, the DREM settings are read from the file **settingsfile.txt**, which has the same format as the defaults file, and the model file **outmodelfile.txt** is automatically saved after the learning procedure terminates. The saved model file can then be loaded into DREM for later viewing. To run DREM in batch mode use the command:

```
java -mx1024M -jar drem.jar -b settingsfile.txt outmodelfile.txt
```

3 Input Interface

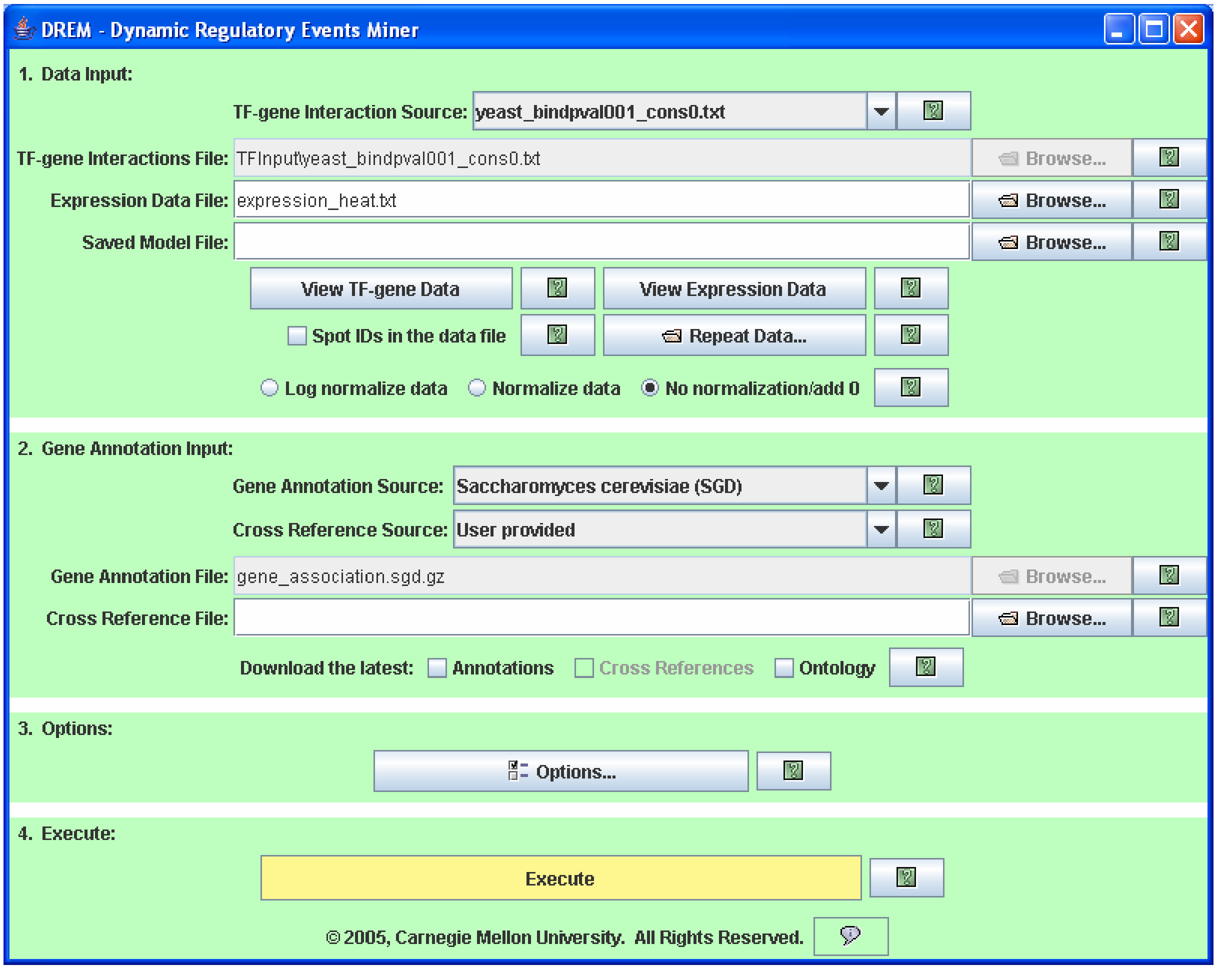

Figure 1: Above is an image of the main input interface of DREM. This is the first screen that appears when DREM is launched. From this screen a user specifies the input data, gene annotation information, and various execution options. Pressing the execute button at the bottom of the interface causes the DREM algorithm to execute.

The first window that appears after DREM is launched is the input interface (Figure 1). The interface is divided into four sections. In the top section a user specifies the file of transcription factor-gene regulation predictions,

the expression data files, normalization options for the expression data, and optionally a previously saved model. In the second section a user specifies the gene annotation information. In the third section a user specifies the various execution options. These three sections of the interface are described in more detail in the next three subsections. In the fourth section of the interface there is a button which when pressed causes the DREM to execute its algorithm to reconstruct a dynamic regulatory map based on the input data and specified options. DREM then displays the map in the output interface described in Section 4.

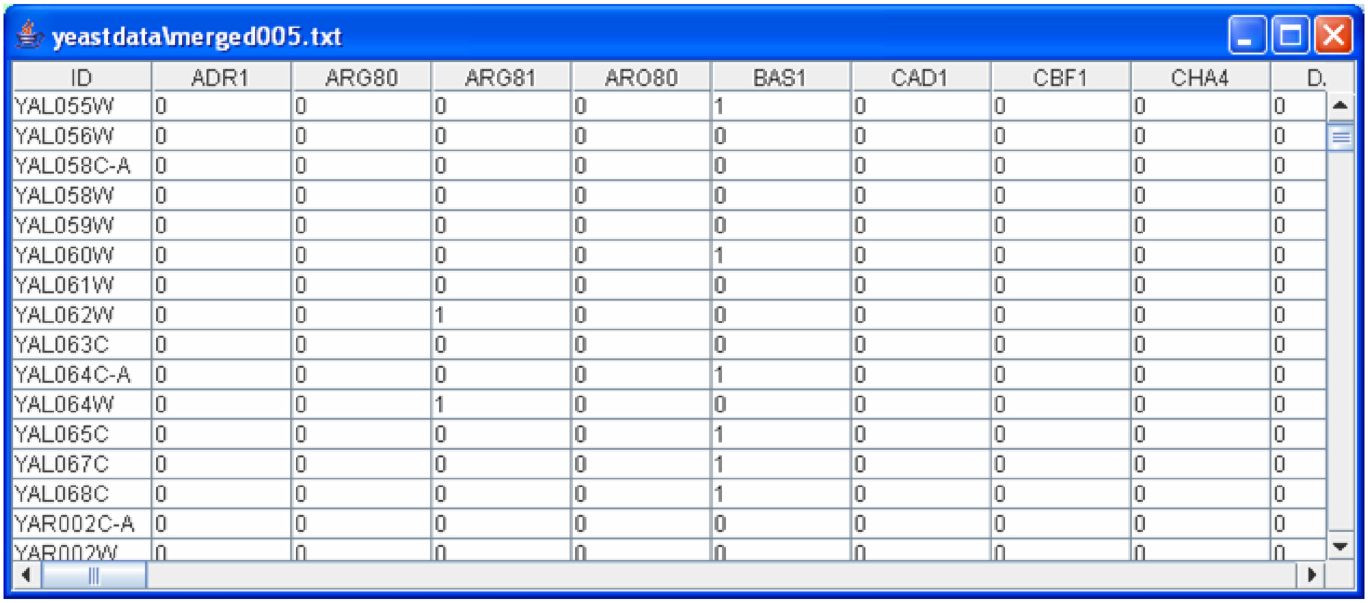

ID	ADR1	ARG80	ARG81	ARO80	BAS1	CAD1	CBF1	CHA4	D.
YAL055W	0	0	0	0	1	0	0	0	0
YAL056W	0	0	0	0	0	0	0	0	0
YAL058C-A	0	0	0	0	0	0	0	0	0
YAL058W	0	0	0	0	0	0	0	0	0
YAL059W	0	0	0	0	0	0	0	0	0
YAL060W	0	0	0	0	1	0	0	0	0
YAL061W	0	0	0	0	0	0	0	0	0
YAL062W	0	0	1	0	0	0	0	0	0
YAL063C	0	0	0	0	0	0	0	0	0
YAL064C-A	0	0	0	0	1	0	0	0	0
YAL064W	0	0	1	0	0	0	0	0	0
YAL065C	0	0	0	0	1	0	0	0	0
YAL067C	0	0	0	0	1	0	0	0	0
YAL068C	0	0	0	0	1	0	0	0	0
YAR002C-A	0	0	0	0	0	0	0	0	0
YAR002W	0	0	0	0	0	0	0	0	0

Figure 2: A sample TF-gene interaction input data file in the grid format displayed in a table after the button *View TF-gene Data* on the input interface was pressed.

3.1 Data Input

3.1.1 Transcription Factor-gene Interactions File

The first field in the data input section of the interface is the *TF-gene Interactions Source* field. Predictions of Transcription Factor (TF)-gene regulation interactions are an input to DREM. The source of these predictions can either be *User Provided* or one of the files that currently is present in the *TFInput* directory of the *drem* directory. The TF-gene Interaction files provided with DREM are described in Appendix B. If *User Provided* is selected then the *TF-gene Interactions File* field is editable and a user can select any file even if it does not currently reside in the *TFInput* directory. Otherwise the *TF-gene Interactions File* field displays the file specified under *TF-gene Interactions Source* and is not editable. The format of a TF-gene interaction file is a tab delimited file. The file can either be an ASCII text file or a GNU zip file of an ASCII text file. The file can be in one of two formats, a grid format or a three column format.

In the grid format the columns correspond to the transcription factors, and the rows correspond to the genes. The first column contains gene symbols. An entry in a column can have multiple names for the same gene delimited by either a comma (‘,’), semicolon (‘;’), or pipe (‘|’). The first row contains the gene symbol column

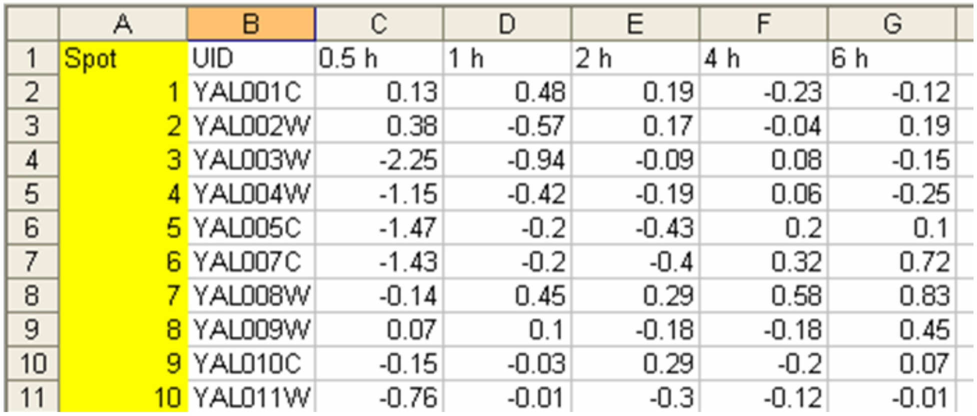

	A	B	C	D	E	F	G
1	Spot	UID	0.5 h	1 h	2 h	4 h	6 h
2	1	YAL001C	0.13	0.48	0.19	-0.23	-0.12
3	2	YAL002W	0.38	-0.57	0.17	-0.04	0.19
4	3	YAL003W	-2.25	-0.94	-0.09	0.08	-0.15
5	4	YAL004W	-1.15	-0.42	-0.19	0.06	-0.25
6	5	YAL005C	-1.47	-0.2	-0.43	0.2	0.1
7	6	YAL007C	-1.43	-0.2	-0.4	0.32	0.72
8	7	YAL008W	-0.14	0.45	0.29	0.58	0.83
9	8	YAL009W	0.07	0.1	-0.18	-0.18	0.45
10	9	YAL010C	-0.15	-0.03	0.29	-0.2	0.07
11	10	YAL011W	-0.76	-0.01	-0.3	-0.12	-0.01

Figure 3: Above is a sample input data file when viewed in Microsoft Excel. The first column, shown in yellow, contains spot IDs and is optional. If the column is included then the field *Spot IDs in the data file* on the input interface must be checked, otherwise the field must be unchecked and the first column contain gene symbols. The columns containing the time series of gene expression values come after the gene symbol column. The sample data in this figure and throughout the manual comes from [8].

header followed by the names of each transcription factor all delimited by tabs. As with genes multiple names for a transcription factor can be given if they are delimited by a comma (','), semicolon (';'), or pipe ('|'). An entry of 0 in the file corresponds to the prediction that there is no regulatory interaction between the transcription factor and the gene. An entry of 1 corresponds to the prediction that the transcription factor does regulate the gene. While not used in the provided files it also possible to differentiate between predicted activating and repression regulatory interactions by using a '1' for predicted activation interactions and '-1' for predicted repression interactions. Pressing the *View TF-gene Data* button allows a user to view the contents of the file specified in the *TF-gene Interaction* field, an example of such is shown in Figure 2.

In the three column format the first column contains the transcription factors, the second column the regulated gene, and the third column input value. The first row is a header row where the header of the first column must be 'TF' column, and the second column must have the header 'Gene'. The format for specifying multiple names for a gene or TF are the same as described above for the grid format. If a TF-gene pair is not present the input value is assumed to be 0. When there are a lot of TFs and genes with a sparse number of non-zero entries then the three column format can lead to significant savings in space.

TF	Gene	Input
BAS1	YAL055W	1
CBF1	YAL053W	1
CBF1	YAL054C	1

Table 1: Example of formatting for TF-gene interaction file in the three column format.

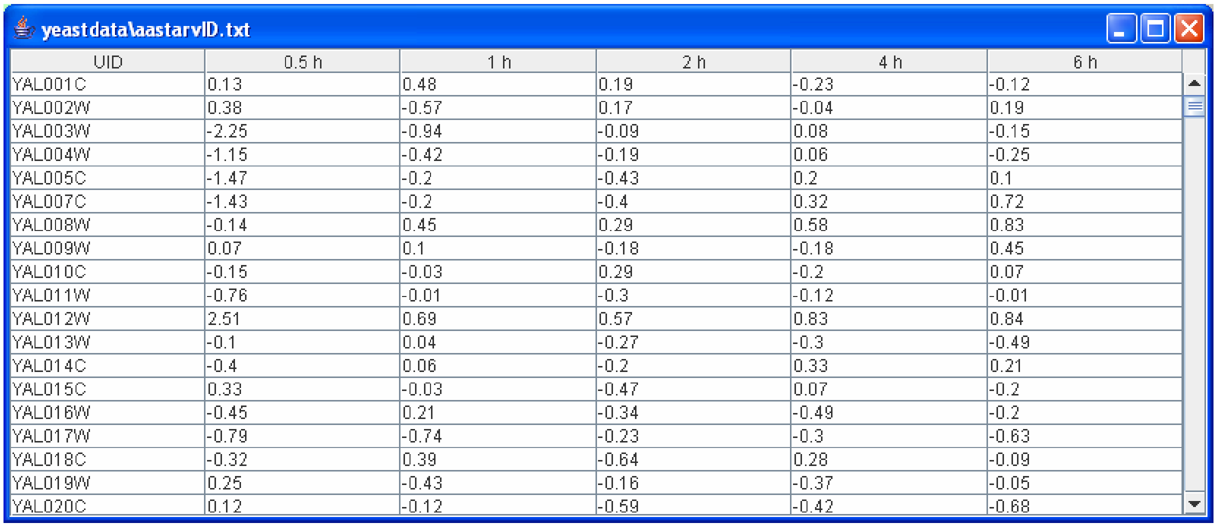
yeastdata\aastarvID.txt

UID	0.5 h	1 h	2 h	4 h	6 h
YAL001C	0.13	0.48	0.19	-0.23	-0.12
YAL002W	0.38	-0.57	0.17	-0.04	0.19
YAL003W	-2.25	-0.94	-0.09	0.08	-0.15
YAL004W	-1.15	-0.42	-0.19	0.06	-0.25
YAL005C	-1.47	-0.2	-0.43	0.2	0.1
YAL007C	-1.43	-0.2	-0.4	0.32	0.72
YAL008W	-0.14	0.45	0.29	0.58	0.83
YAL009W	0.07	0.1	-0.18	-0.18	0.45
YAL010C	-0.15	-0.03	0.29	-0.2	0.07
YAL011W	-0.76	-0.01	-0.3	-0.12	-0.01
YAL012W	2.51	0.69	0.57	0.83	0.84
YAL013W	-0.1	0.04	-0.27	-0.3	-0.49
YAL014C	-0.4	0.06	-0.2	0.33	0.21
YAL015C	0.33	-0.03	-0.47	0.07	-0.2
YAL016W	-0.45	0.21	-0.34	-0.49	-0.2
YAL017W	-0.79	-0.74	-0.23	-0.3	-0.63
YAL018C	-0.32	0.39	-0.64	0.28	-0.09
YAL019W	0.25	-0.43	-0.16	-0.37	-0.05
YAL020C	0.12	-0.12	-0.59	-0.42	-0.68

Figure 4: A sample input expression data file displayed in a table after the button *View Data File* on the input interface was pressed.

3.1.2 Time Specific Binding Data

A new feature is that DREM supports time-specific binding data, as could be derived by conducting ChIP-Chip/-Seq experiments for different time points. In order to recognize time-specific binding data the user has to provide the data in 4-column format which is an extension to the three-column format. A fourth column is added representing the timepoint for that binding value. The time is matched up with the headers from the expression data. If the zero timepoint is being added to the data set by DREM, the number "0" will be recognized as the zero-th timepoint.

TF	Gene	Input	Timepoint
BAS1	YAL055W	1	time1
CBF1	YAL053W	1	time2
CBF1	YAL054C	1	time3

Table 2: Example of formatting for TF-gene interaction file in the four column format for time specific binding events.

3.1.3 Expression Data File

The second field is the *Expression Data File* field. An expression data file consists of gene symbols, time series expression values, and optionally spot IDs. Spot IDs uniquely identify an entry in the data file, and if they are not included in the data file, then they will be automatically generated. While spot IDs must be unique, the same gene symbol may appear multiple times in the data file corresponding to the same gene appearing on multiple spots on the array. Expression values for the same gene will be averaged using the median before further analysis on the data is conducted.

A sample expression data file as it would appear in Microsoft Excel is shown in Figure 3. The first column, which appears in yellow, is optional, and if included contains spot IDs. If the data file includes the spot IDs

column, then the field *Spot IDs in the data file* on the input interface must be checked, otherwise the field must be unchecked. The next column, or the first column if spot IDs are not in the data file, contain gene symbols. If a gene symbol is not available then the field can be left empty or a '0' can be placed in it. Both the spot ID field and the gene symbol field may contain multiple entries delimited by a semicolon (';'), pipe ('|'), or comma (','). The sub-entries in the field are only relevant in the context of gene annotations described in the next section. The remaining columns contain the expression value at each time point ordered sequentially based on time. If an expression value is missing, then the field should be left empty.

The first row of the data file contains column headers. If it is desired that the x-axis be scaled proportional to the actual sampling rate, then each column header must contain the time at which the experiment was sampled in the same units. Each row below the column header corresponds to a spot on the microarray. Each column must be delimited by a tab. The tab-delimited input data file should be an ASCII text file or a GNU zip file of an ASCII text file. A tab-delimited text file can easily be generated in Microsoft Excel by choosing *Text(Tab delimited)* as the *Save as type* under the *Save As* menu. To view the contents of the data file from the interface press the button *View Expression Data* and then a table such as in Figure 4 will appear.

Before gene expression time series are analyzed by DREM, the time series must be transformed to start at 0. The transformation that is used to do this can be selected to be of one of three types: *Log normalize data*, *Normalize data*, or *No normalization/add 0*. Given a time series vector of gene expression values $(v_0, v_1, v_2, ..., v_n)$ the transformations are as follows:

- *Log normalize data* – transforms the vector to $(0, \log_2(\frac{v_1}{v_0}), \log_2(\frac{v_2}{v_0}), ..., \log_2(\frac{v_n}{v_0}))$
- *Normalize data* – transforms the vector to $(0, v_1 - v_0, v_2 - v_0, ..., v_n - v_0)$
- *No normalization/add 0* – transforms the vector to $(0, v_0, v_1, v_2, ..., v_n)$

It is recommended that after transformation a time series represent the log ratios of the gene expression levels versus the level at time point 0. Time point 0 usually corresponds to a control before the experimental conditions were applied. If the input data file contains raw expression values as from an Oligonucleotide array, then the *Log normalize data* option should be selected. If any values are 0 or negative and the *Log normalize data* option is selected, then these values will be treated as missing. If the input data file already represents the log ratio of a sample against a control as is often the case when the data is from a two channel cDNA array and an experiment was conducted at time point 0, then the *Normalize data* option should be selected. In this case after normalization the transformed values will represent the log change ratio versus time point 0. If the input data file already contains log ratio data against a control, but no time point 0 experiment was conducted, then the *No normalization/add 0* option should be selected. In this case the assumption is made that had a time point 0 experiment been conducted the expression level in both channels would have been equal.

Pressing the *Repeat Data* button brings up an interface as shown in Figure 5. The *Repeat Data* button on the main input interface is yellow if there is currently one or more repeat data files specified, otherwise it is gray. Repeat data files must have the same format as the original data file, including the same number of rows and columns. Repeat data values will be averaged with the values from the original data file using the median.

Repeat data can be selected to be from either *Different time periods* or *The same time period*. If the data is from *Different time periods* then data was collected over multiple distinct time series, but presumably at the same sampling rate. If the data is from *The same time period* then this implies multiple measurements were collected

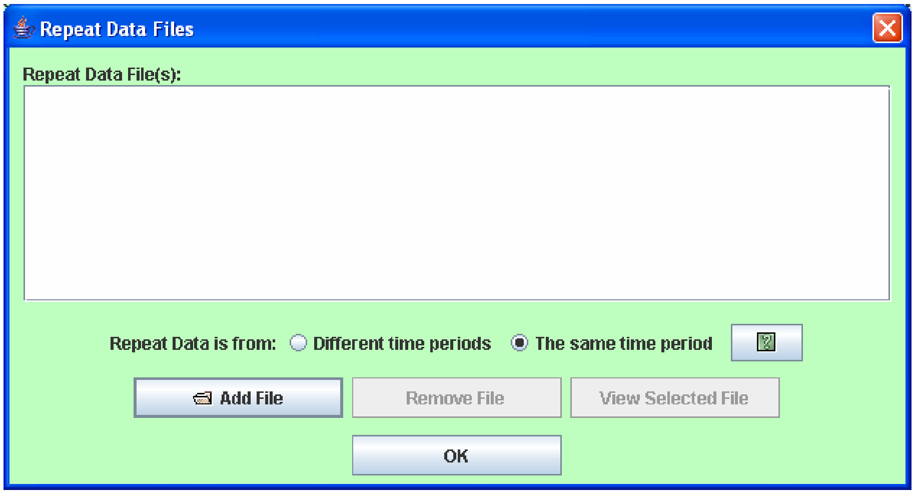

Figure 5: The above window is used to specify repeat data files. A user can add or remove repeat files with the *Add File* and *Remove File* buttons. A user also needs to specify whether the repeat data samples are from the same time period or different time periods as the original data. The contents of a repeat file can be viewed by selecting the repeat file and then pressing the *View Selected File* button.

at each time point during one time series. If the repeat data is selected to be from *The same time period*, then the file to which any two column of values for the same time point belong could be interchanged without effect. In contrast, if the repeat data is selected to be from *Different time periods* this is not the case. If the repeat data is from *Different time periods*, the repeat data will be averaged after normalization, while if the repeat data is from *The Same Time Period* the repeat data will be averaged before normalization. In the case the repeat data is from *Different time periods*, the repeat data can be used to filter genes with inconsistent expression patterns as explained in Section 3.3.1.

3.1.4 Saved Model File

The *Saved Model File* field allows a user to specify a file containing a saved model, thus saving time if the model has already been computed. A saved model file can also be used to initialize from where the search for a model starts. The option controlling how the saved model file is used is determined by the *Saved Model* option on the *Search Options* panel described in Section 3.3.2.

3.2 Gene Annotation Info

In the second section of the interface a user specifies the gene annotation information. Both gene symbols and spot IDs can be annotated as belonging to an official Gene Ontology (GO) category or a user defined category. If a gene is annotated as belonging to an official category in the Gene Ontology, then it will automatically also be annotated as belonging to any ancestor category in the ontology hierarchy. The first field in this section of the interface is the *Gene Annotation Source*. This field can be set to either *User provided*, *No annotations*, or one of 35 annotation data sets provided by Gene Ontology Consortium members. A full list of the 35 data sets can be found in Appendix C. More information about these annotation sets can be found at `http://www.`

	A	B
1	SEPW1	GO:0016491;GO:0000004;GO:0008372
2	PRPF8	GO:0008248;GO:0006397;GO:0005634;GO:0005682
3	PRPF4	GO:0008248;GO:0000398;GO:0008380;GO:0005681
4	JMJD2B	GO:0003677;GO:0006355
5	JMJD2A	GO:0003677;GO:0006355
6	AOX1	GO:0004031;GO:0004854;GO:0005489;GO:0016491;GO:0030151;GO:0006118;GO:0006800;GO:0006954
7	OBP2B	GO:0005215;GO:0005549;GO:0000004;GO:0006810;GO:0007608;GO:0007635;GO:0008372
8	OBP2A	GO:0005215;GO:0005549;GO:0000004;GO:0006810;GO:0007608;GO:0008372
9	PNLIP	GO:0004806;GO:0016787;GO:0006641;GO:0016042
10	STK6	GO:0004674;GO:0005524;GO:0016740;GO:0006468;GO:0007049;GO:0007067;GO:0005634;GO:0005819

Figure 6: Annotation file in a two column format. The first column contains gene symbols or spot IDs while the second column contains category IDs. Annotation files can also be in the official 15 column format.

`geneontology.org/GO.current.annotations.shtml`, and for the annotation sets provided by the European Bioinformatics Institute (EBI) also at `http://www.ebi.ac.uk/GOA/`. One of the 35 data sets is the EBI UniProt set. For a large number of organisms, subsets of this data set with annotations specific to the organism can be found `http://www.ebi.ac.uk/GOA/proteomes.html`. These subset data sets are not included in the list of 35 data sets. If one of the 35 data sets is selected, then the annotation file corresponding to the source will appear in the *Gene Annotation File* text box uneditable. If *User provided* is selected, then the *Gene Annotation File* text box will become editable, and a user can specify a gene annotation file. Selecting *No annotations* is equivalent to selecting *User Provided* and leaving the field empty.

A gene annotation file can be in one of two formats:

1. The gene annotation file can be in the official 15 column gene annotation file format described at `http://www.geneontology.org/GO.annotation.shtml#file`. All 35 of the data sets provided by Gene Ontology Consortium members are in this format. If the file is in this format any entry in the columns *DB_Object_ID* (Column 2), *DB_Object_Symbol* (Column 3), *DB_Object_Name* (Column 10), or *DB_Object_Synonym* (Column 11) will be annotated as belonging to the GO category specified in Column 5 of the row. If the entry in the *DB_Object_Symbol* contains an underscore ('_'), then the portion of the entry before the underscore will also be annotated as belonging to the GO category since under some naming conventions the portion after the underscore is a symbol for the database that is not specific to the gene. The *DB_Object_Synonym* column may have multiple symbols delimited by either a semicolon (';'), comma (','), or a pipe ('|') symbol and all will be annotated as belonging to the GO category in Column 5. Note that the exact content of the *DB_Object_ID*, *DB_Object_Symbol*, *DB_Object_Name*, and *DB_Object_Synonym* varies between annotation source, consult the README files available at `http://www.geneontology.org/GO.current.annotations.shtml` to find out more information about the content of these fields for a specific annotation source.

2. The alternative format for an annotation file is two columns delimited by a tab as illustrated in Figure 6. The first column contains gene symbols or spot IDs and the second column contains category IDs. The entries in each column are delimited by a semicolon (';'), comma (','), or a pipe ('|') symbol. If the same gene symbol or spot ID appears on multiple rows, then the union of all its annotations is used.

Matches between gene symbols in the data file and the annotation file is not case sensitive. Gene annotation files can either be in an ASCII text format or a GNU zip file of an ASCII text file.

Below the *Gene Annotation Source* field, is the *Cross Reference Source* field which controls the entry in the *Cross Reference File* field. Cross references are useful in the case that the naming convention used for genes in the data file is different than what is used in the gene annotation file. A cross reference file establishes that two or more symbols refer to the same gene. Note that the cross references is only used to map between gene symbols, and not spot IDs and gene symbols. The *Cross Reference Source* field gives the option to select either *User Provided*, *No cross references*, or cross references for *Arabidopsis*, *Chicken*, *Cow*, *Human*, *Mouse*, *Rat*, or *Zebrafish* provided by the European Bioinformatics Institute (EBI). If *User Provided* is selected for the cross reference file field, then the *Cross Reference File* field becomes editable, and a user can specify a cross reference file. Any gene symbols listed on the same line in the cross reference file will be considered equivalent. The symbols on a line can be delimited by either a tab, semicolon (';'), comma (','), or a pipe ('|'). As with gene annotations files a cross reference file can either be in an ASCII text file or GNU zip version of an ASCII text file.

At the bottom of the gene annotation section of the interface is the phrase *Download the latest* and then three checkboxes, *Annotations*, *Cross References*, and *Ontology.* If the *Annotations* box is checked, then the file listed in the *Gene Annotation File* box will be downloaded from `ftp://ftp.geneontology.org/go/gene-associations/` unless it is an EBI data source in which case it will be downloaded from `ftp://ftp.ebi.ac.uk/pub/databases/GO/goa/`. If the *Cross References* box is checked, then the file listed in the *Cross Reference File* box will be downloaded from `ftp://ftp.ebi.ac.uk/pub/databases/GO/goa/`. If the *Ontology* field is checked, then the file **gene_ontology.obo** will be downloaded from `http://www.geneontology.org/ontology/gene_ontology.obo`. If the annotation, cross reference, or ontology file is required for use, and not present in the **drem** directory, then the corresponding field will be checked and there will not be an option to uncheck the field forcing download of the file(s). If the *Gene Annotation Source* is set to *User Provided* then there will not be an option to download the gene annotation file, and likewise for the cross reference source field and cross reference file. Upon pressing the execute button, the files corresponding to the checked fields will be downloaded.

3.3 Options

The options can be accessed by pressing the *Options* button on the main input interface. These options are divided into five panels, *Filtering* (Figure 7), *Search Options* (Figure 8), *Model Selection Options* (Figure 9), *Gene Annotations* (Figure 10), and *GO Analysis* (Figure 11), and are discussed in the next subsections.

3.3.1 Filtering Options

Through the parameters on the Filtering panel shown in Figure 7 a user can adjust the criteria DREM uses to filter genes. If a gene is filtered, then it will be excluded from further analysis. Genes can be filtered if they do not show a sufficient response to experimental conditions (*Minimum Absolute Expression Change*), there are too many missing values (*Maximum Number of Missing Values*), or the gene expression pattern over repeats is too inconsistent (*Minimum Correlation between Repeats*). A gene can also be filtered if it does appear in the transcription factor-gene interaction input file. If the *Log normalize data* or *Normalize data* options are selected, a gene will automatically be filtered if its expression value at the first time point is missing. A user can also filter genes by criteria not implemented in DREM, in which case a *Pre-filtered Gene File* should be specified if it is desired that these genes are included in the base set for a GO enrichment analysis. Below is a more detailed description of the parameters on the filtering panel:

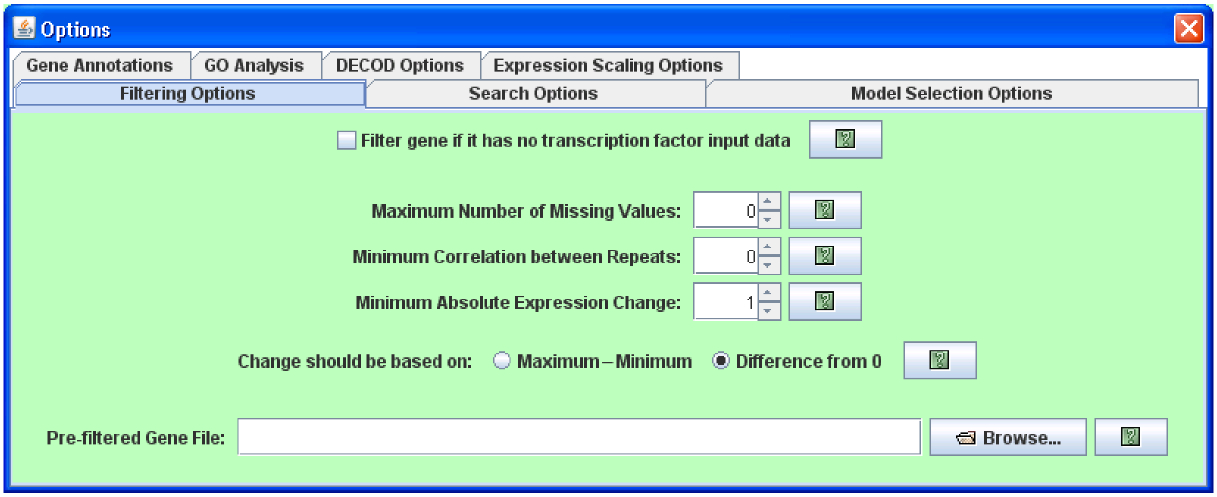

Figure 7: The above panel is used to specify gene filtering options.

- *Filter gene if it has no transcription factor input data* – If this box is checked then genes are filtered if they are not included in the TF-gene interaction file. If this box is unchecked then genes not included in the TF-gene regulation input, are not filtered and are assumed to have a '0' for every entry of the TF-gene regulation predictions.

- *Maximum Number of Missing Values* – A gene will be filtered if the number of missing values in its time series exceeds this parameter.

- *Minimum Correlation between Repeats* – This parameter controls filtering of genes which do not display a consistent temporal pattern across repeat experiments and only applies if there is repeat data selected to be from *Different time periods*. If there is a single repeat file, a gene will be filtered if its correlation between the original data set and the repeat set is below this parameter. If multiple repeats are available, then the gene will be filtered if the mean of all its pairwise correlations between experiments is below this parameter. This parameter is the only place where correlation is used in DREM, and allows the same filtering options as provided in the STEM software [4].

- *Minimum Absolute Expression Change* – After transformation (Log normalize data, Normalize data, or No Normalization/add 0) if the absolute value of the gene's largest change is below this threshold, then the gene will be filtered. How change is defined depends on whether the *Change should be based on* parameter is set to *Maximum−Minimum* or *Difference from 0* (see below).

- *Change should be based on* – The *Change should be based on* parameter defines how change is defined in the context of gene filter. If *Maximum−Minimum* option is selected a gene will be filtered if the maximum absolute difference between the values of any two time points, not necessarily consecutive, after transformation is less than the value of the *Minimum Absolute Expression Change* parameter. If *Difference from 0* is selected a gene will be filtered if the absolute expression change from time point 0 at all time points is less than the value of the *Minimum Absolute Expression Change* parameter.

Formally suppose $(0, v_1, v_2, ..., v_n)$ is the expression level of a gene after transformation and let C be the value of the *Minimum Absolute Expression Change*. If the *Maximum−Minimum* option is selected a gene will be filtered if $max(0, v_1, v_2, ...v_n) - min(0, v_1, v_2, ..., v_n) < C$. If the *Minimum Absolute Expression Change* option is selected the gene will be filtered if $max(0, |v_1|, |v_2|, ..., |v_n|) < C$.

- *Pre-filtered Gene File* – This file is optional. If included, any genes listed in the file will be considered part of the initial base set of genes during a Gene Ontology (GO) enrichment analysis in addition to any genes included in the expression data file. Using this file thus allows one to pre-filter genes from the data by a criteria not implemented in DREM by excluding them from the expression data file, but still include the filtered genes as part of the base set of genes during a GO enrichment analysis. This file does not affect the DREM model or the set of genes in the expression data file and is only relevant to the GO enrichment analysis. If genes appear in both *Pre-filtered Gene File* and the expression data file, then the gene will only be added to the base set once. The format of this file is the same as a data file, except including the time series expression values is optional and if included they will be ignored. As with the expression data file if the field *Spot IDs in the data file* is checked, then the first column will contain spot IDs and the second column will contain gene symbols, otherwise the first column will contain gene symbols.

3.3.2 Search Options

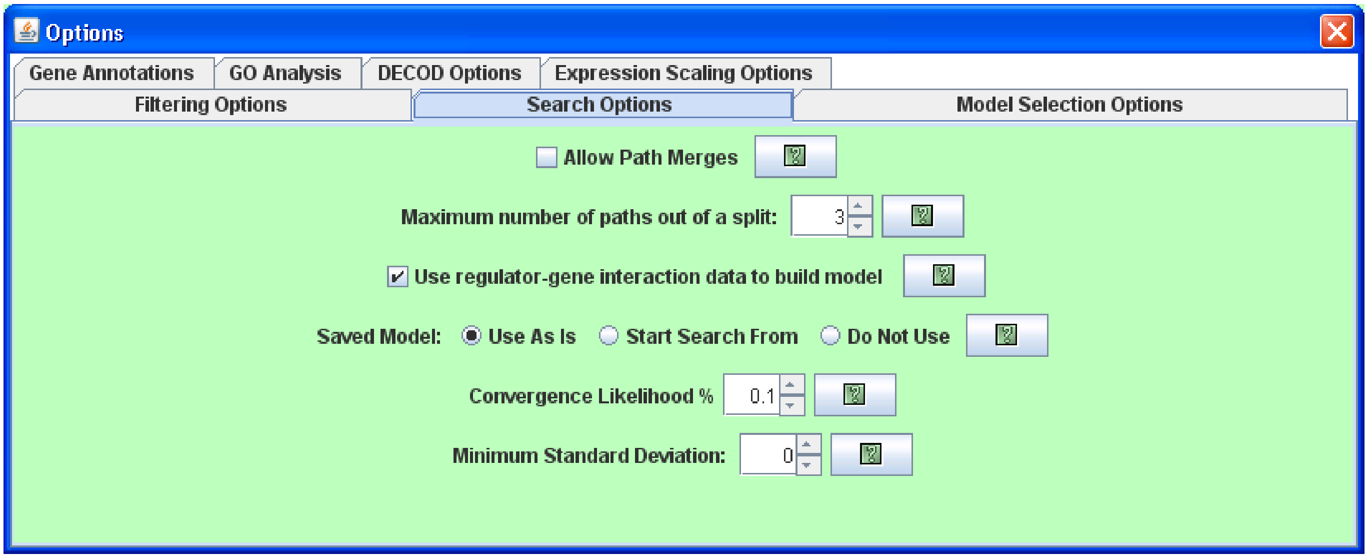

Figure 8: The above panel is used to specify the search options.

The panel used to adjust search options appears in Figure 8 and are discussed below. Model selection options are discussed in the next subsection.

- *Allow Path Merges* – If this field is checked then DREM will consider merging paths that were previously involved in the same split. If this field is not checked then prior merges will not explicitly be modeled to reconverge and the resulting map will always be a tree. Even if the field is checked DREM does not consider re-splitting a path after it is modeled to merge once.

- *Maximum number of paths out of a split* – This parameter controls the maximum number of paths allowed out of a split node. If splits greater than 3 are needed, then it is also worth considering adding time point(s) by interpolation where there are large changes.

- *Use transcription factor-gene interaction data to build model* – If this box is checked then the transcription factor-gene interaction data is used jointly with the time series data to infer the model and then assign genes to paths of the model. If this box is unchecked then the time series data alone is used to infer a model, and the transcription factor-gene interaction predictions are only used in a post-processing step that scores TFs with splits and paths based on the gene assignments. Using the TF-gene interaction data to infer the model gives a more biologically coherent model. When using the TF-gene information only as a post-processing step, the TF-gene scores can be interpreted directly as p-values, which is not the case when the box is checked. Also learning a model is faster when not using the TF-gene interaction data.

- *Saved Model* – This option is only relevant if a file is specified under *Saved Model File.* If the parameter is set to *Use As Is* the model in the *Saved Model File* is opened exactly as is. If the parameter is set to *Start Search From* DREM, and the model does not have any merged paths, DREM will start its search from the model saved in *Saved Model File.* If the parameter is set to *Do Not Use* then DREM will ignore what is specified in the *Saved Model File* field and start a new search.

- *Convergence Likelihood %* – This parameter controls the percentage likelihood gain required to continue searching for better parameters for the model. Increasing this parameter can lead to a faster running time, decreasing it may lead to better values of the parameters.

- *Minimum Standard Deviation* – (new in version 1.0.9b) This parameter controls the minimum standard deviation on the Gaussian distributions. Increasing this parameter is recommended if applying DREM to RNA-seq data to avoid potential overfitting of low variance in expression due to small discrete counts.

3.3.3 Model Selection Options

The *Model Selection Options* panel as shown in Figure 9 contains parameters used to evaluate and select the model DREM presents. Two different frameworks can be used either *Penalized Likelihood* or *Train-Test.* Under the *Penalized Likelihood* option all the genes are used to both train the parameters of the model during search and select the model. A regularization parameter, *Penalized Likelihood Node Penalty*, is the penalty subtracted for each state to prevent overfitting. Model selection under the *Train-Test* option of DREM works in two phases. In the first phase, the main search phase, DREM deletes paths that improve the score and adds paths while the *Train-Test Main search score* improves beyond the threshold specified below. A subset of genes are used to train the parameters of the model, and the log likelihood of the remaining genes are used to score the model. The *Train-Test Random Seed* parameter influences the random partitioning of genes into a training and test set. In the second phase the genes in the training and test set are randomly partitioned again and then DREM tries to delete paths, delays splits, and if path merges are allowed then merge paths sharing a prior split. In this second phase to avoid overfitting the data, simpler models that result in worse scores can still be accepted as long as the resulting scores is within a threshold specified by the parameters below. The parameters are discussed in more detail below. Note that the *Penalized Likelihood Node Penalty* parameter is only active when the *Penalized*

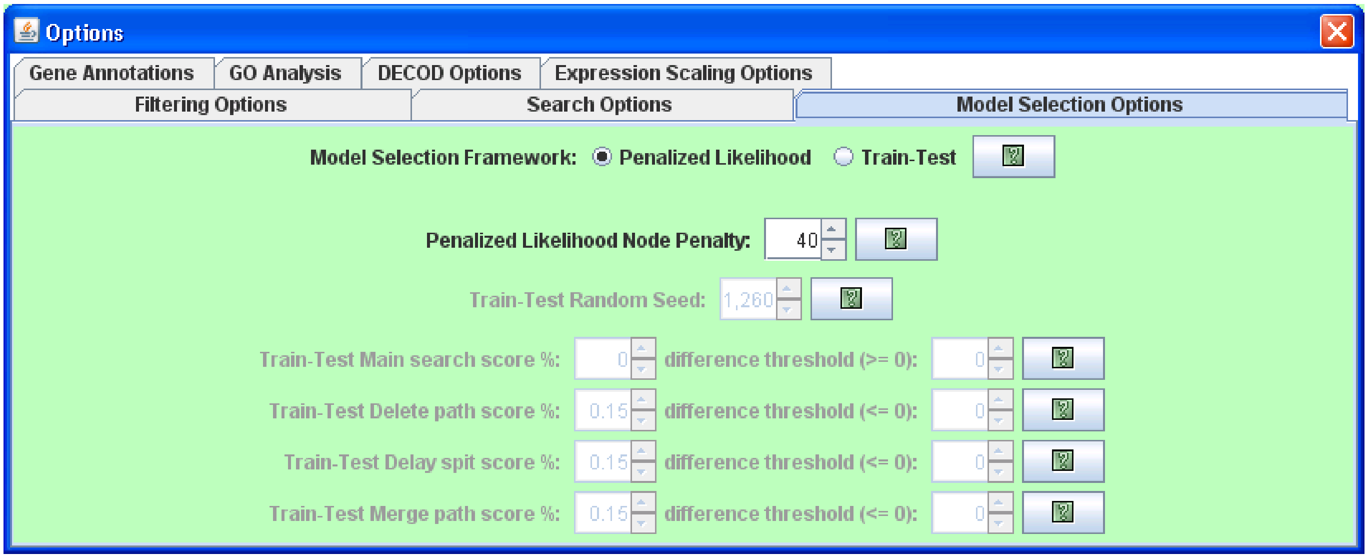

Figure 9: The above panel is used to specify additional search options.

Likelihood option is selected, and the nine parameters below that are only active when the *Train-test* option is selected.

- *Model Selection Framework* – Two frameworks, *Penalized Likelihood* and *Train-Test* for model selection are available.

- *Penalized Likelihood Node Penalty* – This parameter is only active if the *Penalized Likelihood* option is selected under the model selection framework, in which case it is the penalty for each node (state) in the final model. If L is the log likelihood based on all the genes, λ the value of this parameter, and N_{nodes} is the number of nodes in the model then DREM attempts to find a model which optimizes

$$L - \lambda \times N_{nodes}$$

  Increasing the parameter would cause more nodes, while decreasing it will cause fewer.

- *Train-Test Random Seed* – This parameter is the random seed used by DREM for randomly partitioning the data set into a training set and test set. Changing the value of this parameter can result in different maps, though the major features of the maps will usually remain consistent.

- *Train-Test Main search score (% and difference threshold)* – These two parameters determine the minimum score improvement on the test set needed for DREM to continue its search after adding a path during the main search phase of the algorithm. Let S_{new} be the score of the model after adding a path, S_{old} is the score of the model from the previous iteration, ϵ_{main} the % parameter, and D_{main} the difference threshold parameter. It is required that D_{main} be greater than or equal to 0. For DREM to continue searching after adding a path the equation

$$S_{new} - \epsilon_{main} \times |S_{new}| - S_{old} > D_{main}$$

must be satisfied. Note that if D_main, is set to 0, then the requirement becomes simply that the score improvement percentage exceed ϵ_{main} where the percentage is based on the score of the new model. If ϵ_{main} is set to 0, then the requirement becomes simply that the new model score must exceed the old model score by D_{main}. Increasing these parameters can lead to the search ending sooner, but potentially returning a model that is not as good.

- *Train-Test Delete path score (% and difference threshold)* – These parameters controls the removal of weakly supported paths during the second phase of the DREM algorithm. Let S_{new} be the score of the model after deleting a path, S_{old} the score of the model without the path deleted, ϵ_{delete} the % parameter, and D_{delete} the difference threshold parameter. It is required that D_{delete} be less than or equal to 0. For DREM to continue searching after adding a path the equation

$$S_{new} + \epsilon_{delete} \times |S_{new}| - S_{old} > D_{delete}$$

  must be satisfied. Note that if D_{delete} is set to 0, this requirement becomes simply that the score difference between the old and new model exceed the ϵ_{delete} where the percentage is based on the score of the new model. If ϵ_{delete} is set to 0, then the difference between the new model score and the old model score must exceed the value of D_{delete}. Increasing the percentage parameter or decreasing the difference threshold parameter will lead to more paths being deleted.

- *Train-Test Delay path score (% and difference threshold)* – These parameters controls the score change threshold to delaying splits during the second phase of the DREM algorithm. These parameters work analogously to the Delete path parameters described above.

- *Train-Test Merge path score (% and difference threshold)* – These parameters control merging paths from a common split during the final phase of the DREM algorithm if path merging is allowed. These parameters work analogously to the Delete path parameters described above.

3.3.4 Gene Annotations Options

On the fourth panel, shown in Figure 10, a user may specify options related to gene annotations. The first three options allow one to filter annotations when the annotation file is in the official 15 column format. The last field, the *Category ID mapping file*, is useful in the case in which genes are annotated as belonging to a category outside the Gene Ontology. The options on this panel are as follows:

- *Only include annotations of type* {*Biological Process, Molecular Function, Cellular Component*} – These three checkboxes allow one to filter annotations that are not of the types checked. These three checkboxes only apply if the annotations are in the official 15 column GO format, in which case the annotation type is determined by the entry in the *Aspect* field (Column 9). An entry of P in the *Aspect* field means the annotation is of type *Biological Process*, an entry of F means the annotation is of type *Molecular Function*, and an entry of C means the annotation is of type *Cellular Component*.

- *Only include annotations with these taxon IDs* – Some annotation files contain annotations for multiple organism, and it might be desirable to use only annotations for certain organisms. To use only annotations for certain organisms enter the taxon IDs for the desired organisms delimited by either commas (‘,’),

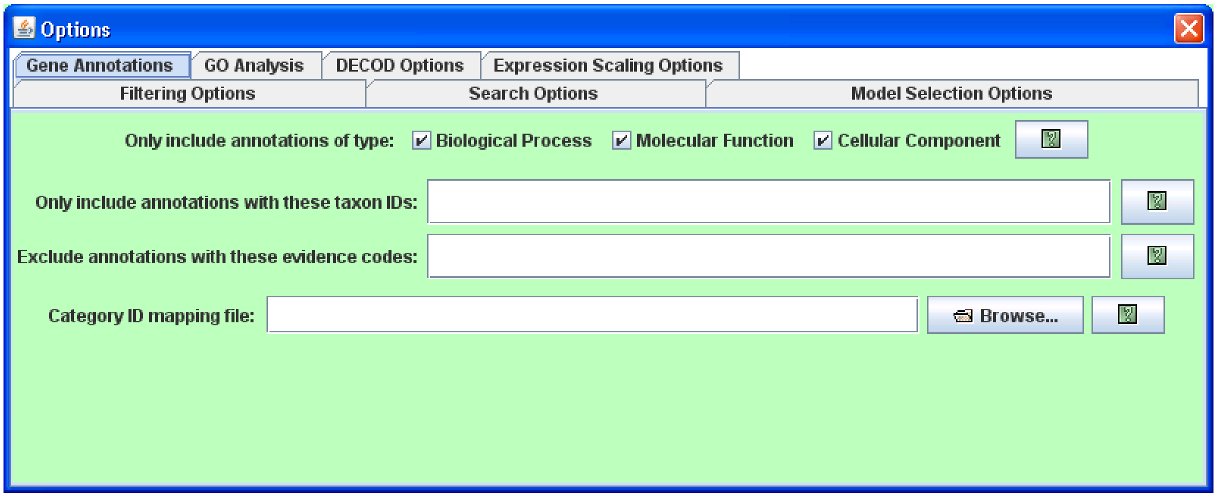

Figure 10: The above panel is used to specify options related to gene annotations.

semicolons (';'), or pipes ('|'). If this field is left empty, then any organism is assumed to be acceptable. More information about taxonomy codes and a search function to find the taxon code for an organism can be found at `http://www.ncbi.nlm.nih.gov/Taxonomy/`. Note that this parameter only applies when the annotations are in the official 15 column format. The taxonomy ID in the annotation file is in column 13 of the file, and the taxon IDs entered in this parameter field must match the entry in column 13 or match after prepending the string 'taxon:' to the ID. For example to use only annotations for a *Homo sapien* the string *9606* can be used.

- *Exclude annotations with these evidence codes* – This field takes a list of unacceptable evidence codes for gene annotations delimited by either a comma (','), semicolon (';'), or pipe ('|'). If this field is left empty, then all evidence codes are assumed to be acceptable. Evidence code symbols are *IEA, IC, IDA, IEP, IGI, IMP, IPI, ISS, RCA, NAS, ND, TAS, and NR*. Information about GO evidence codes can be found at `http://www.geneontology.org/GO.evidence.codes.shtml`. Note that this field only applies if the gene annotations are in the official 15 column GO annotation format. The evidence code is the entry in column 7. For example to exclude the annotations that were inferred from electronic annotation or a non-traceable author statement the field should contain *IEA;NAS*.

- *Category ID mapping file* – This file, which is optional, specifies a mapping between gene category IDs and category names for categories which are not official Gene Ontology categories. The mapping between IDs and names for official GO categories are defined in the file **gene_ontology.obo**. If a category ID appears in the gene annotation file, but does not correspond to an official Gene Ontology category and is not defined in a *Category ID mapping file*, then the category ID is used in place of the category name. A category ID mapping file has two columns delimited by a tab. The first column contains category IDs and the second column contains category names. Each line defines a mapping between one category ID and names. Below is a short sample file:

```
ID_A     CategoryNameA
```

```
ID_B     CategoryNameB
ID_C     CategoryNameC
```

3.3.5 GO Analysis Options

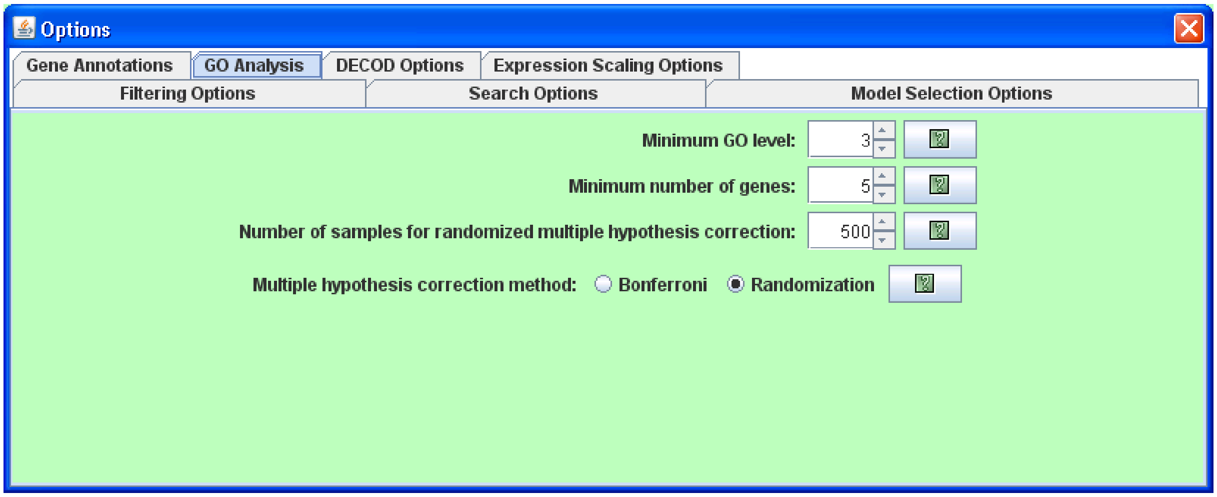

Figure 11: The above panel is used to specify options for the Gene Ontology enrichment analysis.

The next options panel, shown in Figure 11, controls options related to Gene Ontology (GO) enrichment analysis. Note that categories that appear in a gene annotation file even if not part of the official Gene Ontology, are also included in a GO analysis. The parameters included on this panel are as follows:

- *Minimum GO level* – Any GO category whose level in the GO hierarchy is below this parameter will not be included in the GO analysis. The categories Biological Process, Molecular Function, and Cellular Component are defined to be at level 1 in the hierarchy. The level of any other term is the length of the longest path to one of these three GO terms in terms of the number of categories on the path. This parameter thus allows one to exclude the most general GO categories.

- *Minimum number of genes* – For a category to be listed in a gene enrichment analysis table, described in Section 4.10, the number of genes in the set being analyzed that also belong to the category must be greater than or equal to this parameter.

- *Number of samples for randomized multiple hypothesis correction* – This parameter specifies the number of random samples that should be made when computing multiple hypothesis corrected enrichment p-values by a randomization test. A randomization test is used when *Randomization* is selected next to the *Multiple hypothesis correction method for actual sized based enrichment* label. GO enrichment computations are based on the actual size of the set, there are no expected size enrichment calculations as in the STEM software [4]. Increasing this parameter will lead to more accurate corrected p-values for the randomization test, but will also lead to longer execution time to compute the values.

- *Multiple hypothesis correction method for actual sized based enrichment* – This parameter controls the correction method for actual size based GO enrichment. The parameter value can either be *Bonferroni*

or *Randomization*. If *Bonferroni* is selected then a Bonferroni correction is applied where the uncorrected p-value is divided by the number of categories meeting the *Minimum GO level* and *Minimum number of genes* constraints. If *Randomization* is selected the corrected p-value is computed based on a randomization test where random samples of the same size of the set being analyzed is drawn. The number of samples is specified by the parameter *Number of samples for multiple hypothesis correction*. The corrected p-value for a p-value, r, is the proportion of random samples for which there is enrichment for any GO category with a p-value less than r. A Bonferroni correction is faster, but a randomization test generally leads to lower p-values.

3.3.6 DECOD Options

The new options tab for running the discriminative DNA motif finder DECOD [11] is shown in Fig. 12. The button(s) to run DECOD at a split node is only visible if the path to the DECOD executable is set, see Section 4.14.

- *Gene to Fasta Format File* – A fasta file with DNA sequences. The header of the file should contain the gene id used in the expression data. The next example shows the format for two DNA sequences for the genes with the IDs MRPL24 and TCF12:

```
>MRPL24
ATCGTTCGATCAGTCGCCATAAT
>TCF12
ATCGACACTACTACTCTCTCTAC
```

- *Path to DECOD Executable* – Use the Browse button to put the path to the DECOD.jar file that will be used by DREM to start the motif search at a split node (see Section 4.14).

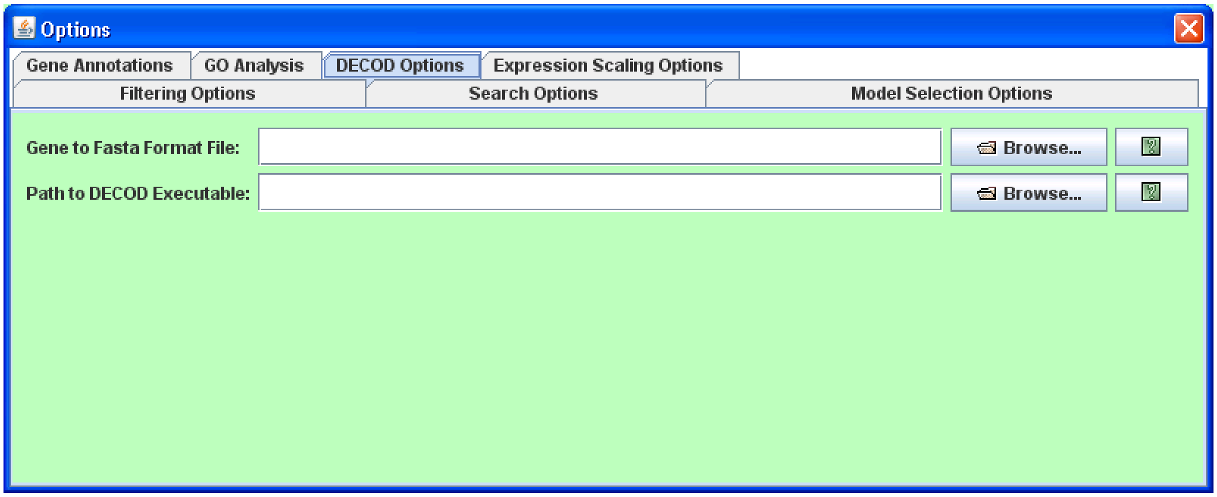

Figure 12: The above panel is used to specify options for DECOD.

3.3.7 Expression Scaling Options

The next options tab shown in Fig. 13 enables the feature to use the TF expression level in the model learning for DREM. The idea is that TFs that are over or under expressed might have an increased or decreased effect on gene regulation, respectively.

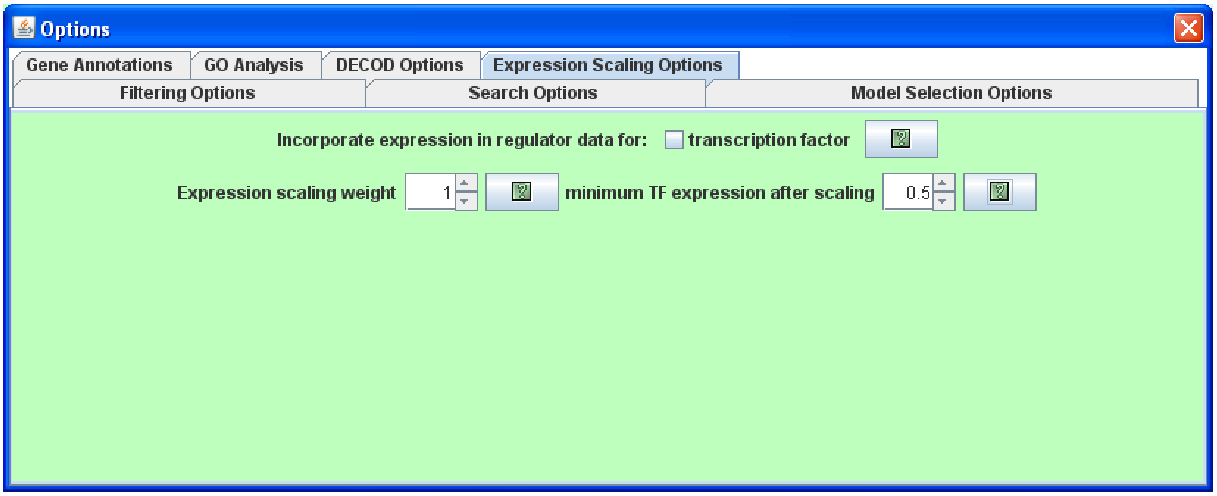

Figure 13: The above panel is used to specify options for regulator expression scaling.

- *Incorporate expression for regulator data* – This checkbox enables the use of the TF expression levels for learning DREM models.
- *Expression scaling weight* – The weight for the logistic function that can be used to adjust the steepness of the function. The default is 1. Values smaller than 1 decrease the effect of the scaling, values close to one approach a step-function.
- *minimum TF expression after scaling* – The minimum absolute value obtained after using the logistic function. If this value is set to 0 TFs that do not change their expression level between time points, or are not expressed will not be used for learning. Default value is 0.5.

3.4 Search Progress Dialog

In Figure 14 is an image of a search progress dialog window. A search progress window appears after pressing the *Execute* button on the DREM input interface, and remains displayed until the output interface appears. There are two buttons in this window. The buttons are the *Display Current Model* and *End Search Button*. Pressing the *Display Current Model* displays the current best map DREM has found so far, but does not end the search. Pressing the *End Search Button* forces DREM to end the main phase of its search. DREM then proceeds to the second phase of its search where it considers deleting paths, delaying paths, and optionally merging paths, but does not consider adding paths anymore.

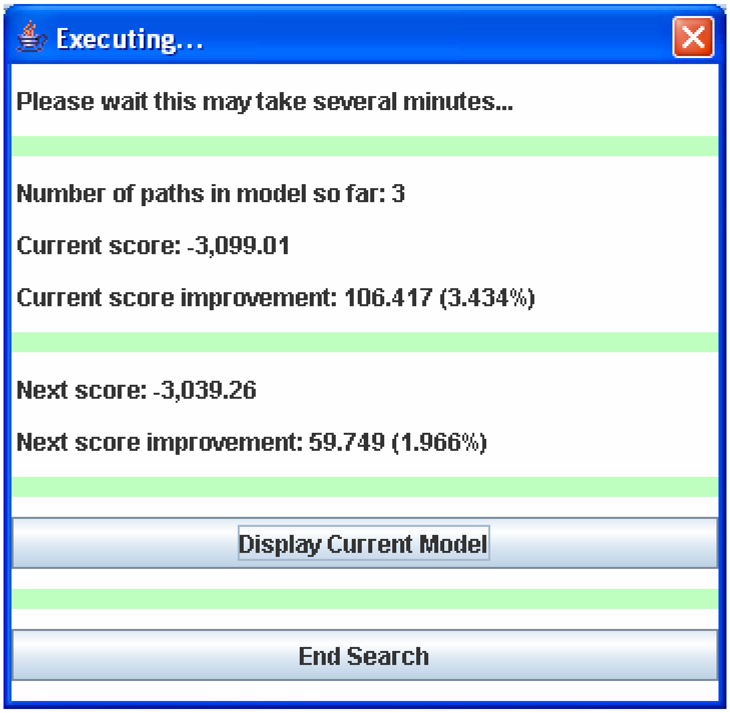

Figure 14: Example of a search progress window.

4 DREM Main Output Interface

After the DREM algorithm executes, the main output window appears. The main window displays the time series of all the genes that were not filtered overlayed with a DREM map. An example of such a window is shown in Figure 15. The DREM map features the major paths and splits in the time series data. Genes are assigned to paths through the model. The paths and splits are annotated with associated transcription factors (see Section 4.4). Each node is associated with a Gaussian distribution. The Gaussian distribution associated with the node determines its y-axis location on the map. The area of a node is proportional to the Gaussian's standard deviation. A relatively small node implies the expression of the genes going through that node will be tightly centered around the node. A relatively large node indicates genes assigned to the path through that node will not necessarily pass closely through the node. Green nodes represent split nodes, these are nodes for which multiple paths exit the node.

Left clicking on an edge displays only genes assigned to a path going through that edge. For instance Figure 16 shows the interface after clicking on the blue edge of Figure 15. Left clicking on a green split node displays all genes passing through the split node. The genes will be colored based on the edge color of the path to which they were assigned out of the split node. Right clicking on an edge or a node which is not a split node brings up a path table as described in Section 4.13. Right clicking on a split node brings up a Split Table as described in Section 4.14. Holding the mouse over a specific gene expression plot displays the name of the gene.

The main interface is zoomable by holding down the right mouse button and moving the mouse (see Figure 18). The interface window can be panned by holding down the left mouse button and moving the mouse. The ability to zoom and pan is powered by the Piccolo software [1]. Zooming scales both axes equally, however to rescale just one axis, the option is available under the *Interface Options* menu described in Section 4.3.

The significant regulator annotations can be moved by left clicking and dragging the text box. After moving an annotation text box, a line will be drawn from the upper left corner of the text box to the path or split at which the regulators are significant (see Figure 19).

Along the bottom of the interface are 12 larger buttons, *Hide/Show Time Series*, *Hide/Show Nodes*, *Interface Options*, *Select by TF*, *Select by GO*, *Select by Gene Set*, *Key TF Labels*, *Predict*, *Gene Table*, *GO Table*, *Save Model*, and *Save Image*. The purpose of each button will be discussed in the next subsections. There are also to smaller buttons. The help button and a disk button. The disk button saves the parameters used to generate the viewed model and some of the interface options.

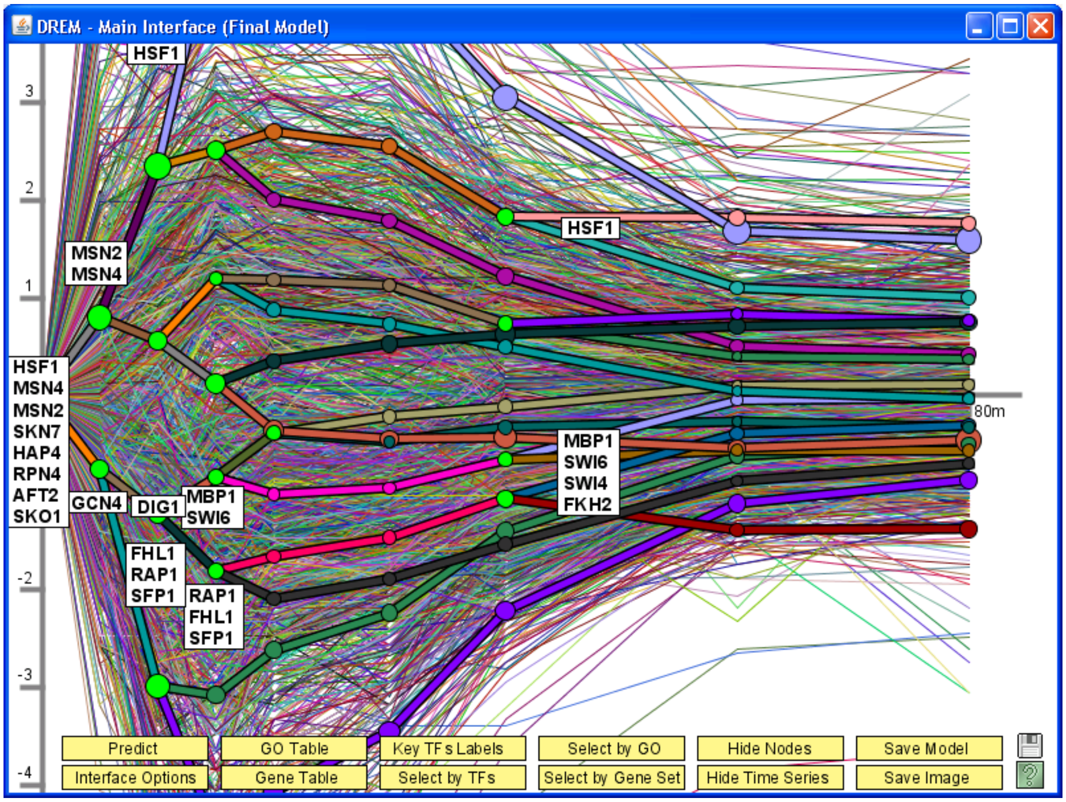

Figure 15: An example of a main output interface window of DREM. The interface has a map overlayed on top of the time series expression profiles. The area of a node is proportional to the standard deviation of the distribution of genes associated with it. Green nodes represent split nodes and have more than one path associated with them. Left clicking along the nodes or edges of the map shows the set of genes assigned to that path. Right clicking on a node or edge brings up more information about the node or edge. Along the bottom are buttons with various options.

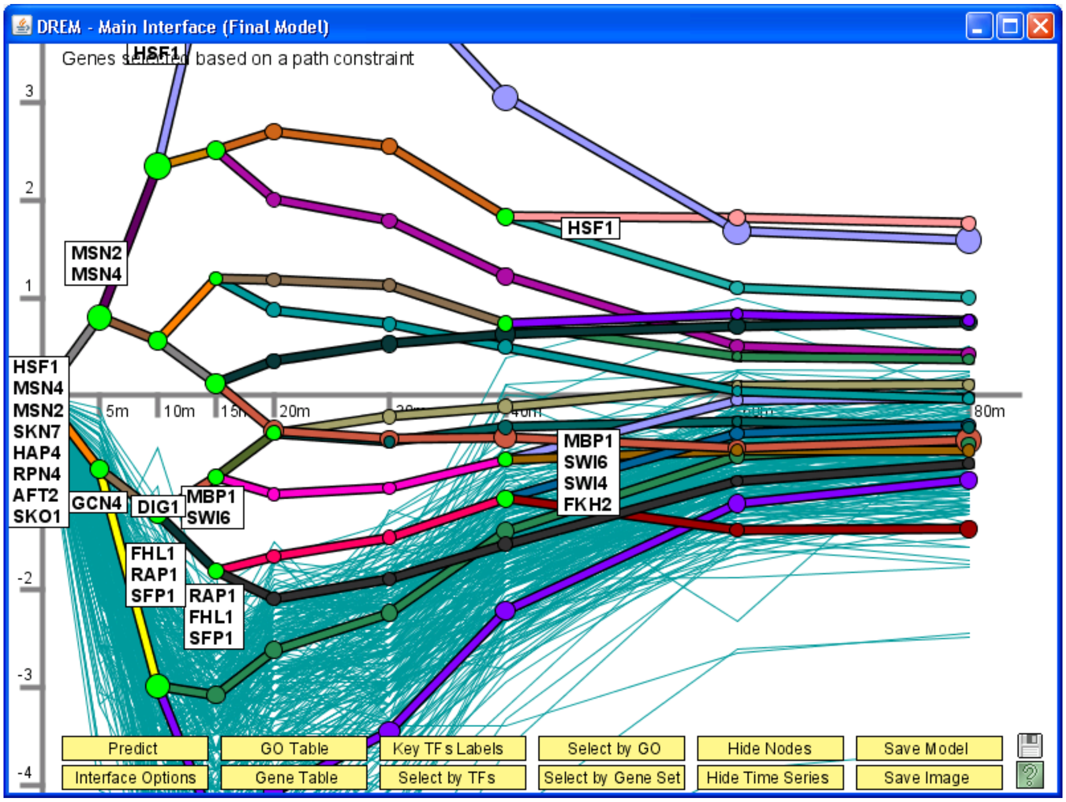

Figure 16: The main interface window of DREM from Figure 15 after clicking on one of the path edges, the edge that appears yellow. Only genes assigned to a path going through this edge appear. If *Automatically Adjust* under *Interface Options* is selected for gene colors, then the genes will have the same color as the edge clicked on.

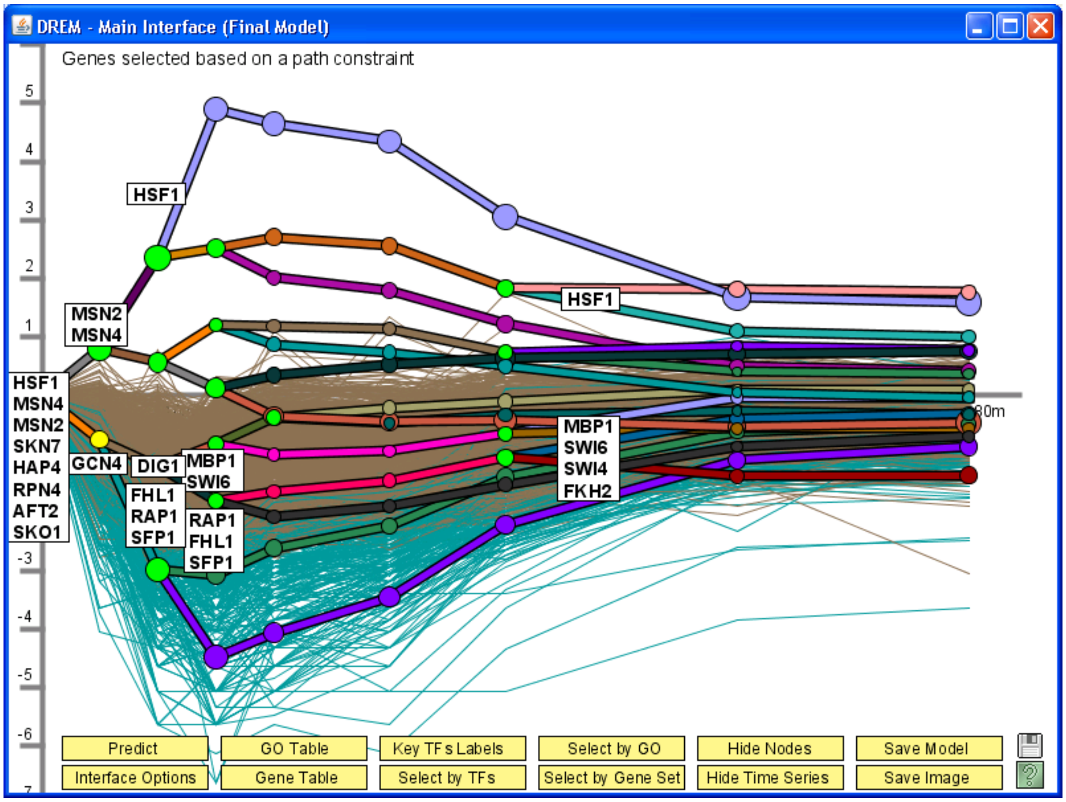

Figure 17: The main interface window of DREM from Figure 15 after clicking on one of the nodes, the node that now appears yellow. Only genes assigned to a path going through the node appear. The genes are colored based on whether there were assigned to the higher or lower path out of the node.

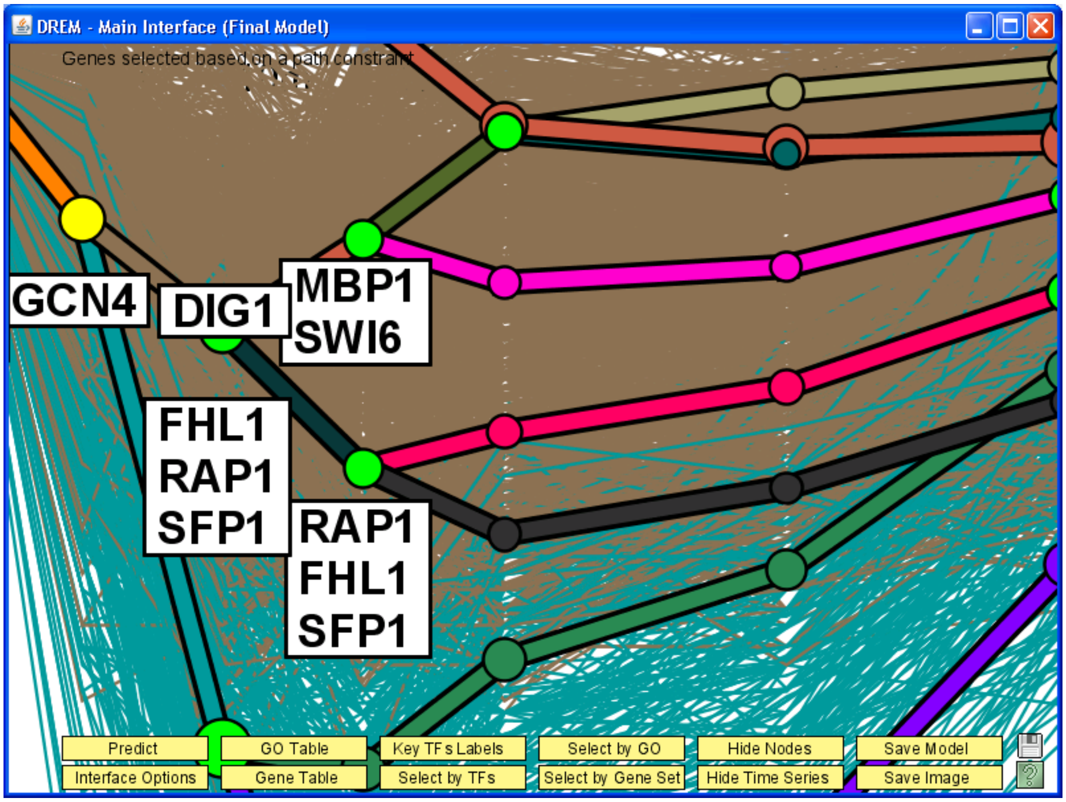

Figure 18: As this image shows, one can zoom and pan on the DREM main interface window. To zoom hold the right mouse button down and move the mouse. To pan hold the left mouse button down and move the mouse. Zooming can also be done through the *Interface Options* menu.

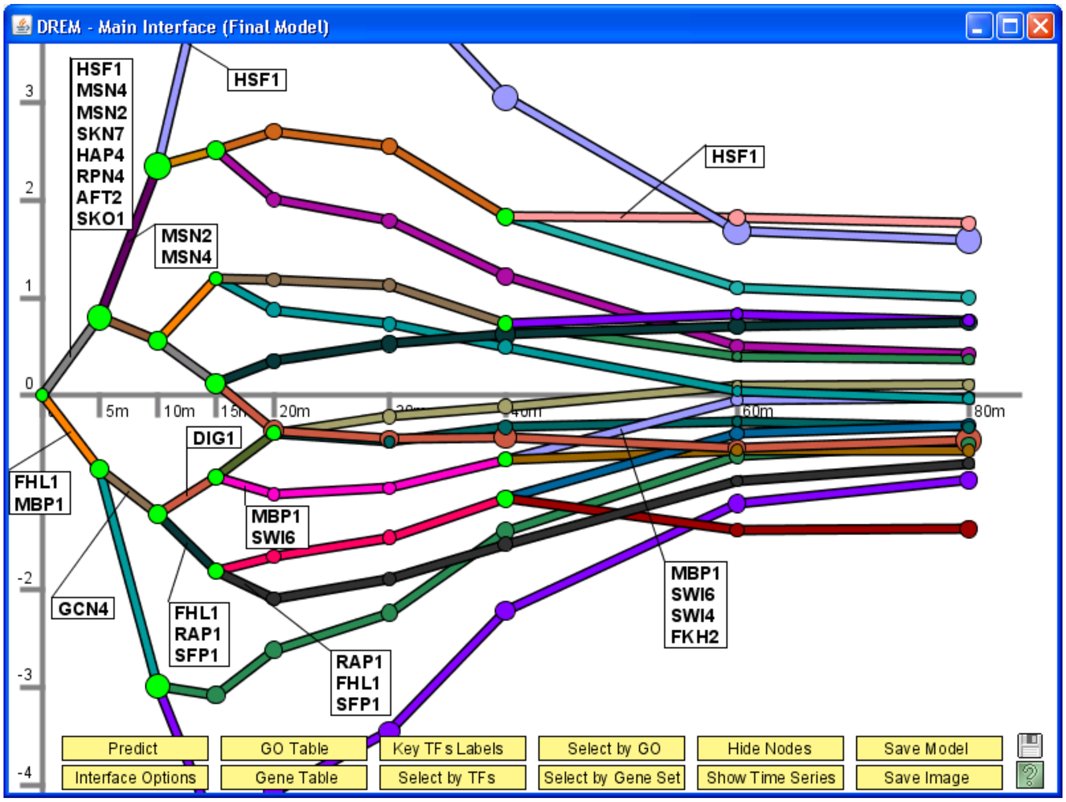

Figure 19: The significant regulator annotations can be moved when they overlap one another or obscure the paths. To move an annotation, left click and drag the text box.

4.1 Hide/Show Time Series

Along the bottom of the interface when the main interface window first appears is a button labeled *Hide Time Series*. When pressing the *Hide Time Series* button the time series plots of all the genes are hidden. After pressing the *Hide Time Series* button, it now reads *Show Time Series* (see Figure 20 for an example). Pressing the *Show Time Series* button reverts DREM back to its previous state with the time series plots showing.

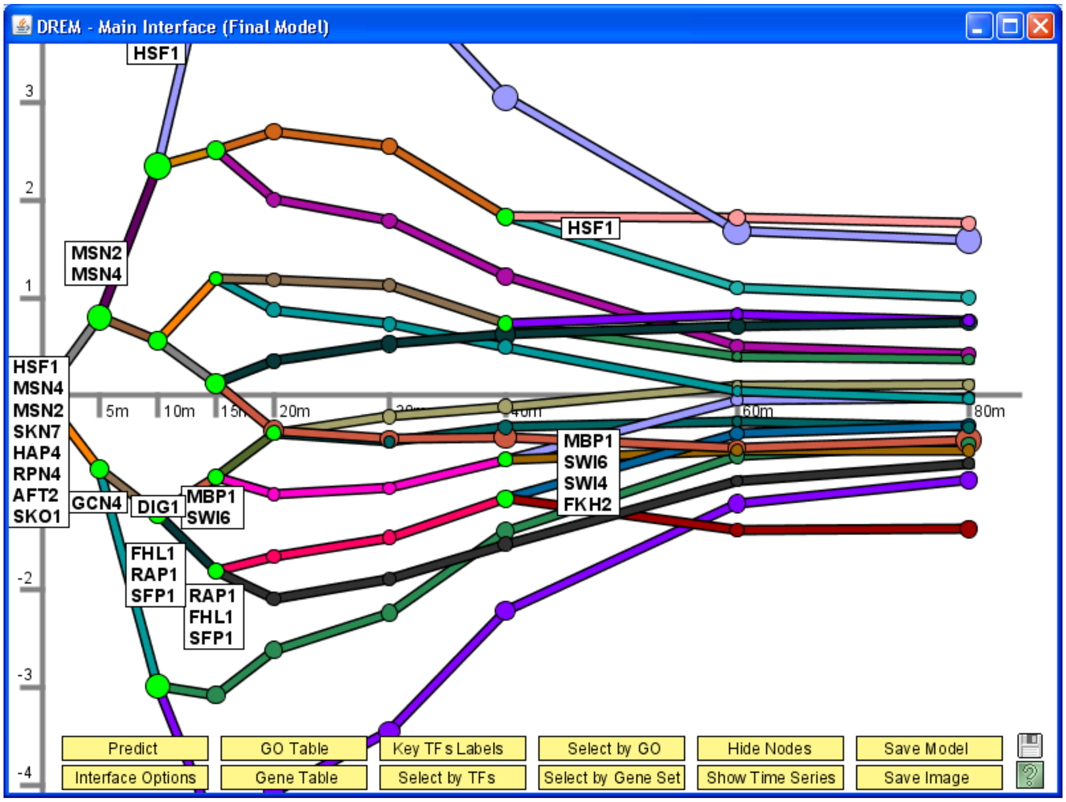

Figure 20: The main interface window of DREM from Figure 15 after pressing the *Hide Time Series* on the main interface window button.

4.2 Hide/Show Nodes

Along the bottom of the interface when the main interface window first appears is a button labeled *Hide Nodes*. When pressing the *Hide Nodes* button the edges and nodes of the dynamic regulatory map are hidden. If the option *Hide All Labels When Hiding Nodes* is selected under the interface options 4.3 then the labels will also be hidden along with the nodes. After pressing the *Hide Nodes* button, it now reads *Show Nodes* (see Figure 21 for an example). Pressing the *Show Nodes* button reverts DREM back to its previous state.

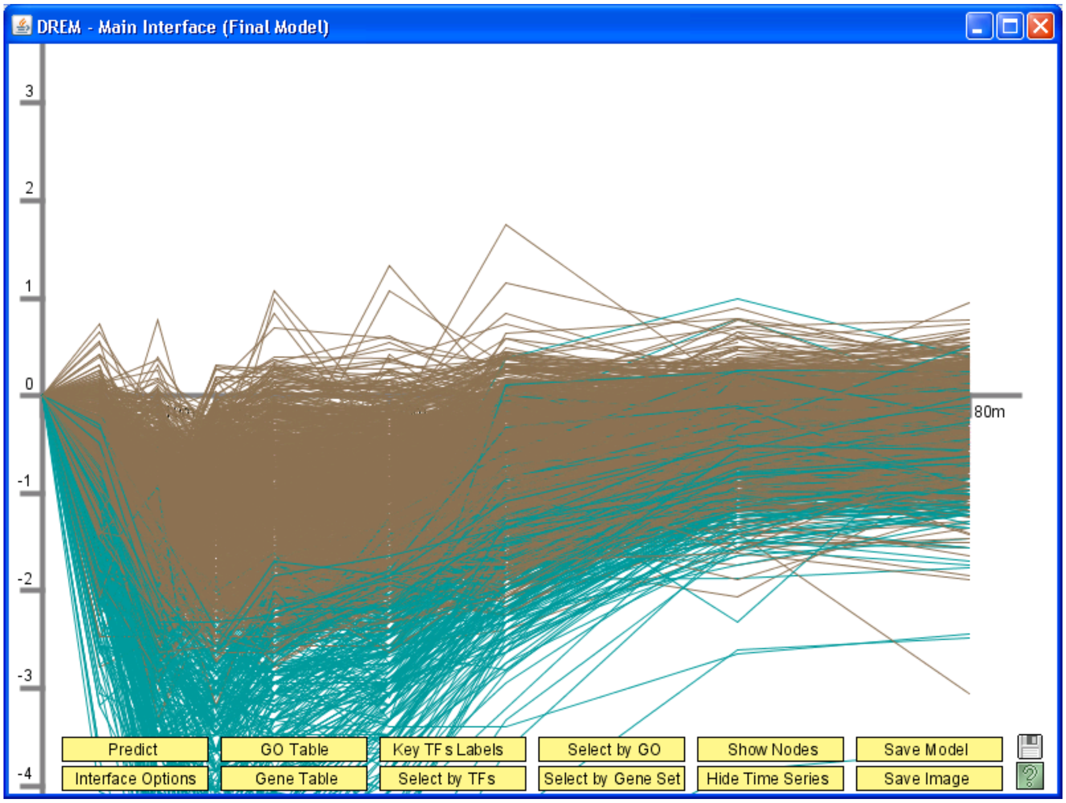

Figure 21: Screenshot of the interface window of Figure 17 after pressing the *Hide Nodes* button.

4.3 Interface Options

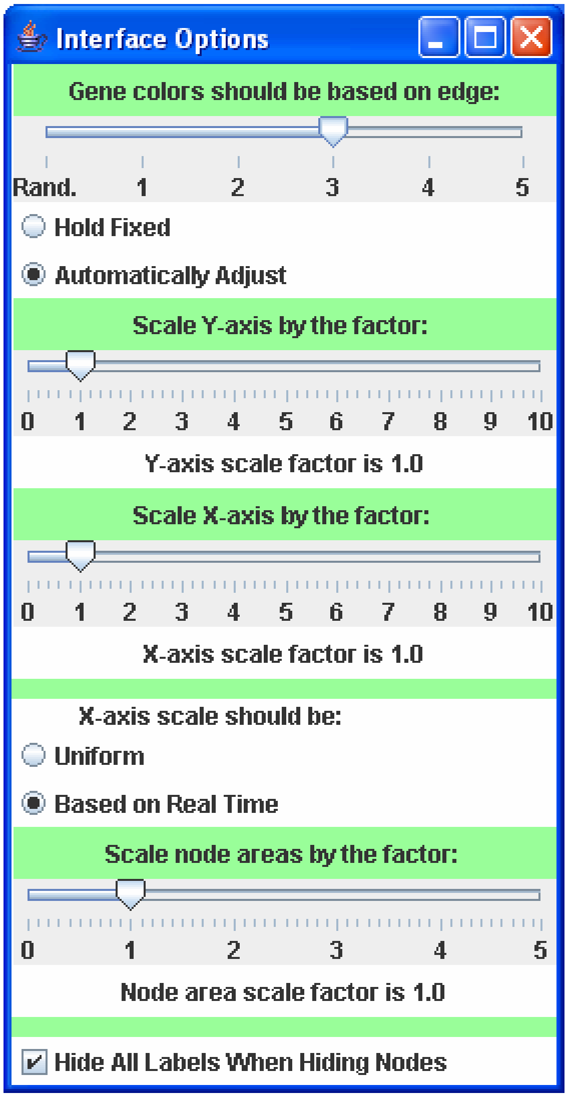

Figure 22: The dialog window to change interface options related to the main output interface window.

Figure 22 shows the menu of options that appears when pressing the button *Interface Options*. The first option is *Gene colors should be based on edge* determines the color of the time series on the main interface. By default all time series have random colors. If this parameter is set to 1, then the time series of a gene will have the same color as the edge between time point 0 and the next time point of the path on the DREM map to which the gene was assigned. In general if the parameter is set to i a time series has the same color as the ith edge of the path to which it is assigned in the DREM map. The next option determines whether DREM should *Hold Fixed* the *Gene colors should be based on edge* parameter value or *Automatically Adjust* it based on the edge or a node of the DREM map a user clicked. If *Automatically Adjust* is selected the value of the parameter will be set to correspond to the node or edge the user clicked on.

The next two options, *Scale Y-axis by the factor* and *Scale X-axis by the factor*, allow one to adjust the y-scale and x-scale of the main window. The default scale for the x and y-axes are multiplied proportional to the value of this parameter.

The *X-axis scale should be* option can either be set to *Uniform* in which case each time point is uniformly spaced on the screen independent of the real sampling rate or it can be *Based on Real Time* in which case the spacing of time points is based proportional to the sampling rate.

The *Scale node areas by the factor* slider allows a user to scale the area of the nodes on the main interface proportional to the value of this parameter. Each individual node will continue to have an area proportional to the standard deviation of the distribution of genes associated with it.

The final option *Hide All Labels When Hiding Nodes* determines if the labels are also hidden when a user presses the *Hide Nodes* button on the main interface. If the box is not checked then just the nodes and edges will be hidden, but not the labels.

4.4 Key TFs Labels

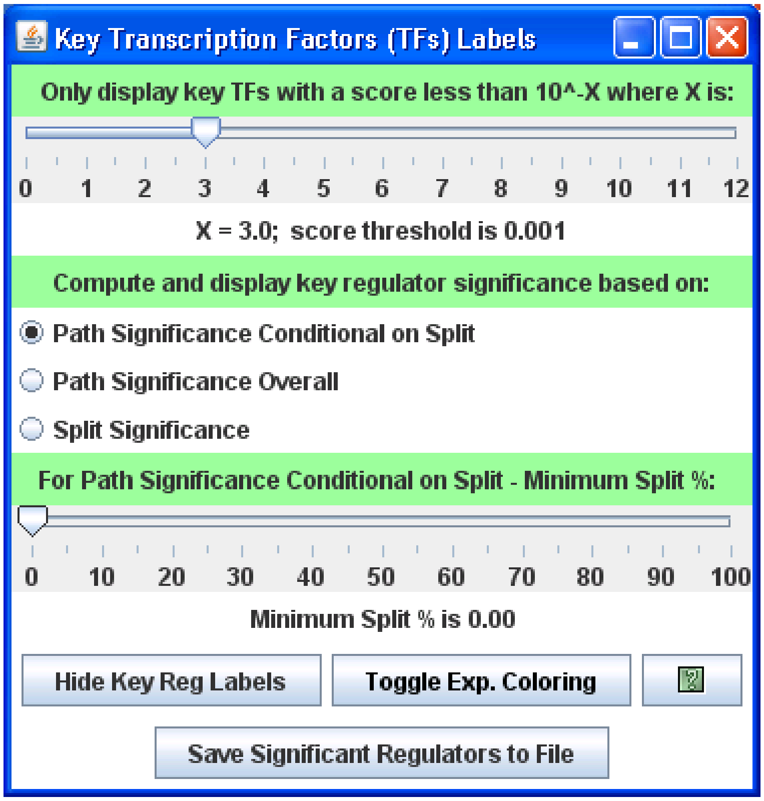

Figure 23: The window that appears after pressing the *Key TFs Labels* button.

The above dialog box, which appears after pressing the *Key TF Labels* button controls the transcription factors labels that appear on the map. The top slider determines the score threshold for a transcription factor label to appear on the map. The slider is based on a negative log base 10 scale, for instance if the slider is on 3, then only scores below 10^{-3} will appear on the map. A lower score for a transcription factor means the more strongly the transcription factor is associated with the path or split. Within a box transcription factors are ordered based on their association with the path or split. Scores can be defined in one of three ways:

- *Path Significance Conditional on Split* - computes using the hypergeometric distribution the score of seeing as many genes annotated to be regulated by the transcription factor that were seen, based on how many genes were annotated by the transcription factor going into the split. The transcription factor box of labels appears after the split and to the immediate left of the next node on its path after the split. If both '-1' and '1' values are included in the input file then '1' TFs annotations are considered separately from '-1' annotations.
- *Path Significance Overall* - computes using the hypergeometric distribution the score of seeing as many genes annotated to be regulated by the transcription factor that were seen based on the total number of genes regulated by the transcription factor in the original data file. In this case the transcription factor box

of labels appears to the immediate left of each node on its path. If both ‘-1’ and ‘1’ values are included in the input file then ‘1’ TFs annotations are considered separately from ‘-1’ annotations.

- *Split Significance* - computes a single score for the significance of a transcription factor at a particular split without differentiating its influence between higher and lower paths and the influence of ’1’ and ‘-1’ inputs. If the prediction file only contains 0’s and 1’s then using *Path Significance Conditional on Split* will likely be preferable. For a two-way split, the difference of the average value of the inputs transcription factor on each path is computed. The split score is based on the probability that a random configuration would lead to a greater absolute difference. For a multi-way split the score becomes the minimum based on all one versus all other paths comparisons.

There is a second slider at the bottom which can be used to be further filter which input labels appear on the map if the option *Path Significance Conditional on Split* is selected. This slider also requires that a certain minimum percentage of genes regulated by the transcription factor going into the split are also regulated by the transcription factor on the path out of the split. In some case it may be desirable to use a less strict threshold on the score threshold and to raise this threshold. Along the bottom of the window are two buttons *Hide Key TF Labels* and *Change Labels Colors*. Pressing the *Hide Key Reg Labels* causes the labels to be hidden. The button then reads *Show Key Reg Labels* and pressing it again will causes the labels to reappear. Pressing the *Change Labels Color* button brings up a dialog window to change the color of the transcription factor labels. The current color of the labels is the same as the text of the button. If Expression scaling is used for the model learning, section 3.3.7, an additional button *Toggle Exp. Coloring* can be used to activate and deactivate that Significant Regulators are shown *blue* or *red* if they are *over or under expressed*, respectively. The button *Save Significant Regulators to File* allows to save the Regulator names of all significant regulators at the currently selected threshold and save them to a file. This is a quick method to use this type of data in a post processing step.

4.5 Select by TFs

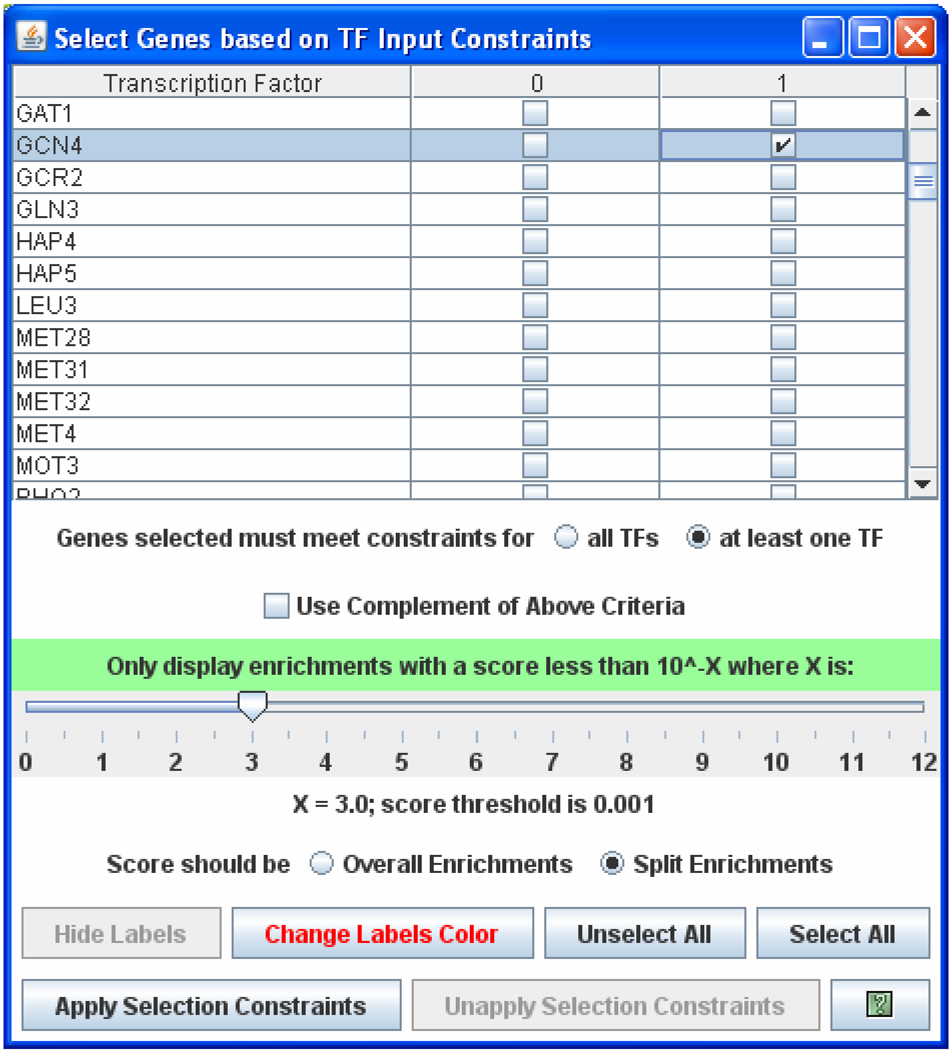

Figure 24: The dialog box that appears when the *Select by TFs* button is pressed on the main interface. This window allows one to select a subset of genes based on being regulated by a certain transcription factor or combination of transcription factors. The above selection will display only genes predicted to be regulated by GCN4.

Figure 24 shows the dialog box when a user presses the *Select by TFs* on the main window of the DREM interface. This dialog box allows a user to view a subset of genes based on being regulated by a common transcription factor (TF) or combination of TFs. For each TF from the *TF-gene Interactions File*, there is a checkbox for the values of '0' and '1'. If '-1' values are also present in the *TF-gene Interactions File*, then there are also checkboxes for this value. If the option *Genes selected must meet constraints for* is set to *all TFs*, then only genes which have TF-gene interaction values matching a checked box value for all TFs will be selected. In this case at least one value must be specified for every TF otherwise it is not possible to have a match. If the option is set to *at least one TF*, then any gene with a predicted TF-gene regulation interaction that matches a checked box for at least one TF will be selected. If the option *Use Complement of Above Criteria* is selected the

complement of the set of genes described by the above criteria will be selected. To actually apply changes made to the checkboxes the button *Apply Selection Constraints* must be pressed. Pressing the button *Unapply Selection Constraints* removes selection constraints based on TF-gene regulation interactions. To have all the checkboxes selected press the button *Select All*, and to have no checkboxes selected press the button *Unselect All*.

In addition to selecting genes, when the *Apply Selection Constraints* button is pressed labels appear when the score for any set of genes is less than the score threshold determined by the setting of the slider under *Only display enrichments with a score less than* 10^{-X} *where X is.* The score can be based on *Split Enrichments* or *Overall Enrichments* for genes regulated by the selected TF regulation constraints. Split enrichments are computed based on the hypergeometric distribution where the base set of genes are all genes going into the prior split on the path. The base set of genes for *Overall Enrichments* is all genes included in the expression data file or the *Pre-filtered Gene File*. Overall enrichments are currently only supported when selecting by a single TF. Labels appear to the immediate right of the first node on the path out of the split. The label contains the number of genes and then the score separated by a semi-colon. To hide labels press the *Hide Labels* button. When the labels are hidden the button now reads *Show Labels*, and pressing it reverts the labels to being shown again. The color of labels can be changed through the *Change Labels Color* button. The color of the TF labels will match that of the color of the text of this *Change Labels Color* button.

4.6 Select by GO

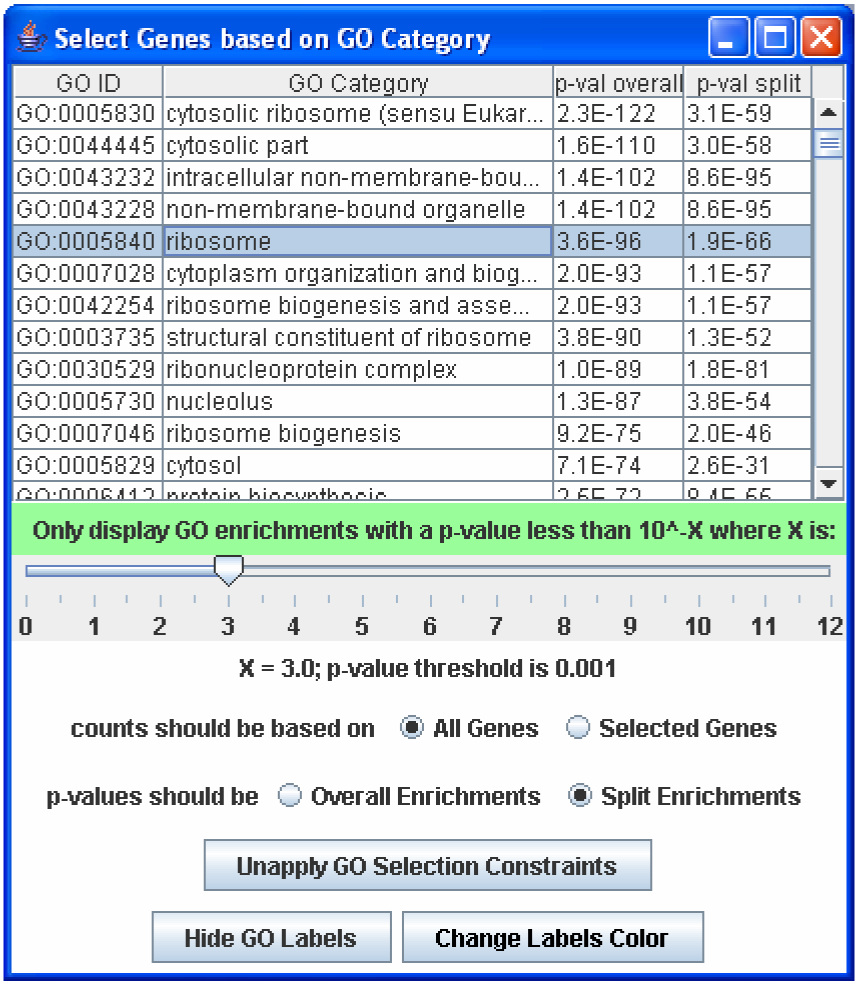

Figure 25: The window that appears after pressing the *Select by GO* button.

After pressing the button *Select by GO* on the main interface, a window such as in Figure 25 appears. The window allows one to reduce the set of genes currently displayed on the main interface to those that also belong to a certain GO category (see Figure 26). The GO category is selected by clicking on a row of the table. To change the GO category one simply needs to click on a different row of the table. To no longer select genes by any GO category press the *Unapply GO Selection Constraints* button. When genes are selected by a GO category, significant p-values appear on the map to the immediate right of nodes on the map. The threshold for significant p-values is defined based on the value on the slider. Let X be the value of the slider then 10^{-X} is the p-value threshold. The *counts should be based on* can be set to *All Genes* or *Selected Genes*. Under the *All Genes* options the counts and enrichments calculations consider all genes going through the path. Under the *Selected Genes* option counts and enrichments calculations consider only the set of genes going through the path and meeting the other selection constraints (Selection by TF and Gene Set). There is also the option *p-values should be*, which can be *Overall Enrichments* or *Split Enrichments*. Overall enrichments compute p-value where the base set of

genes is all genes in the expression data file or the *Pre-filtered Gene File*. Split enrichments are based on just the genes assigned to the prior split. Pressing the *Hide Labels* button hides these labels on the map. To change the colors of these labels press the *Change Labels Color* button. The color of the text of this button will match the color of the GO labels on the map.

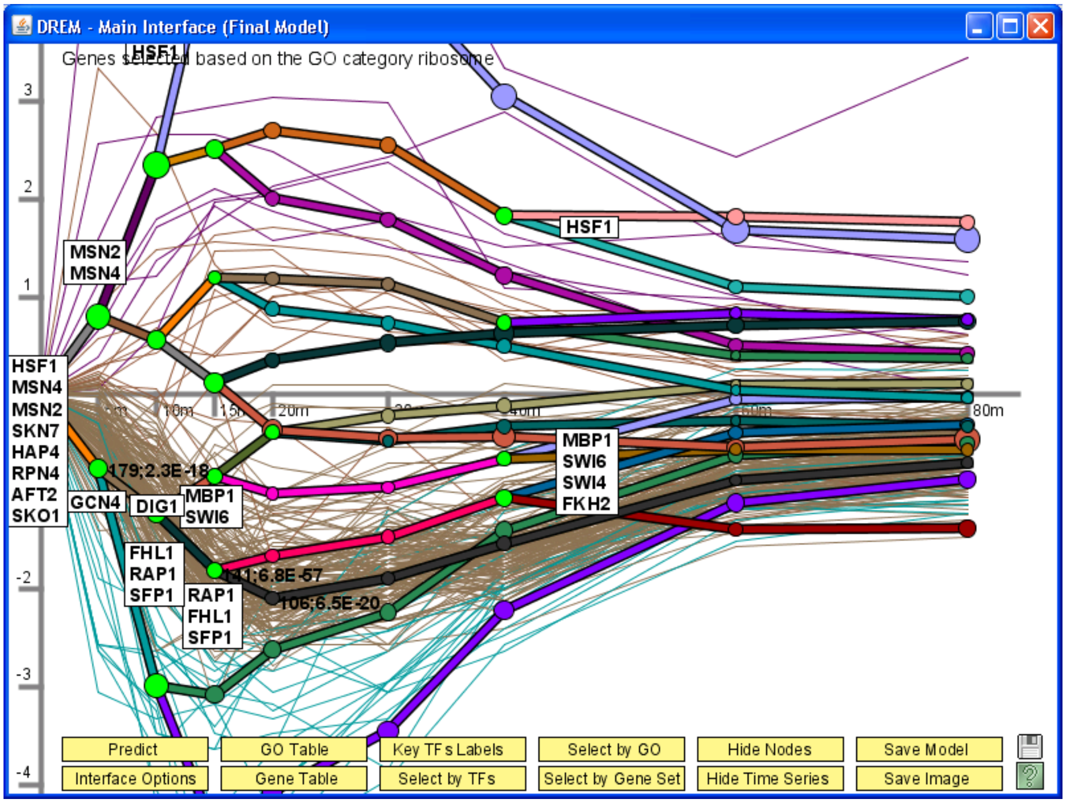

Figure 26: The window that appears after pressing the *Select by GO* button and selecting the ribosome category. Only ribosome genes are display. Labels appear where the significant enrichment for ribosome genes, in this case, computed based on split enrichments.

4.7 Select by Gene Set

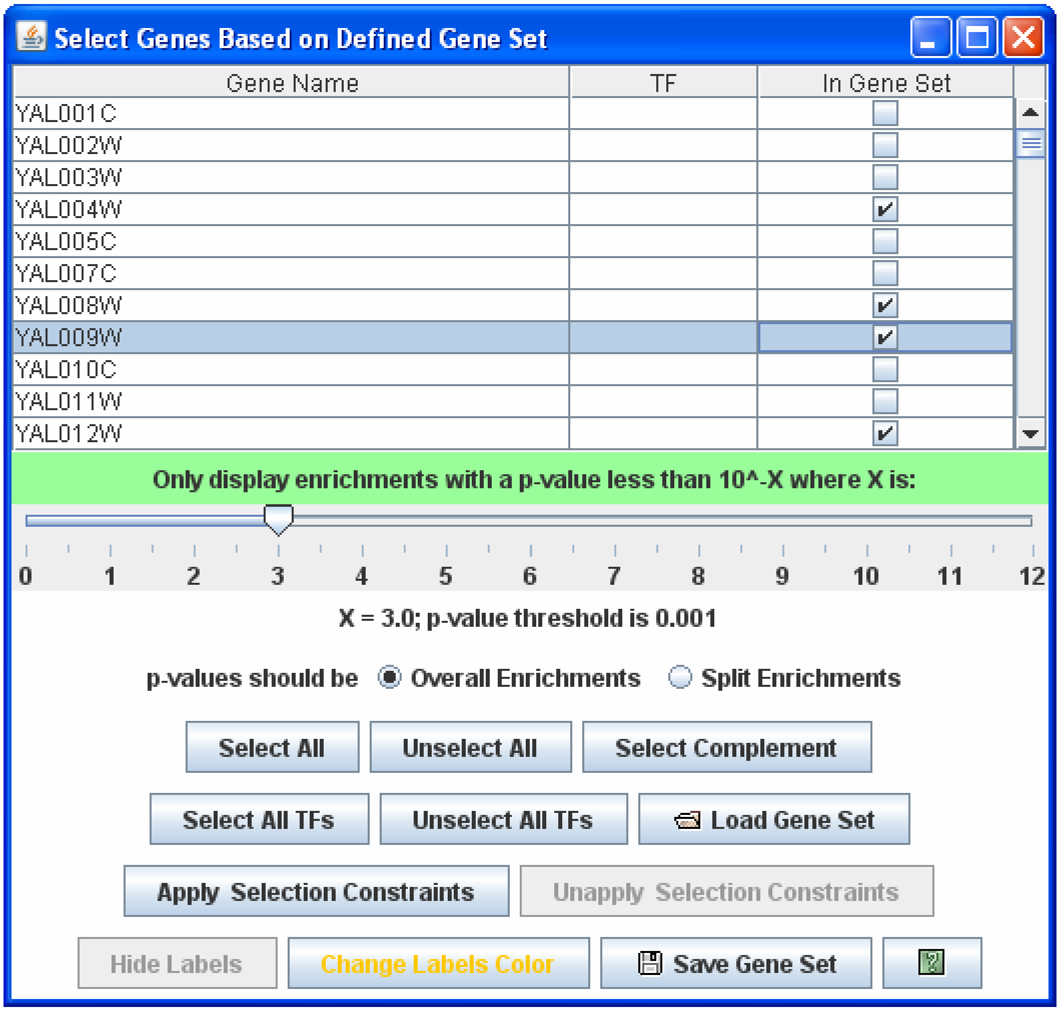

Figure 27: The dialog box appears when one presses the *Select by Gene Set* button on the main interface. This window allows a user to define a subset of genes to be selected.

The above dialog allows a user to select a subset of genes based on the gene names. In order to select a subset one must select the corresponding boxes of the desired genes, and then press the *Apply Selection Constraints* button. Pressing the button *Unapply Selection Constraints* removes the filter based on the gene set but does not clear the checkboxes. When a gene set is selected labels for paths enriched for the gene set at a p-value determined by the slider appear. P-values can either be *Split enrichments* which uses the genes going into the prior split as the base set for the enrichment calculation, or *Overall Enrichments* which uses all the genes on the microarray as a base set.

Below are a description of the additional buttons on this window:

- *Select All* – checks all the gene boxes
- *Unselect All* – unchecks all the gene boxes

- *Select Complement* – checks all currently unchecked boxes and unchecks all currently checked boxes
- *Select All TFs* – checks all the genes which also appear in a column header of the TF-gene interaction file
- *Unselect All TFs* – unchecks all the genes which also appear in a column header of the TF-gene interaction file
- *Apply Selection Constraints* – selects on the main interface only those genes meeting the selection constraints
- *Unapply Selection Constraints* – removes any selection requirement from the last time the apply selection constraints button was pressed
- *Change Label Colors* – pressing the button opens a dialog window to change color of gene set p-value significance labels. The current color of the significance labels are the same of the text of the button.
- *Hide Labels* – hides the p-value significance labels
- *Load Gene Set* – option to select the genes listed in a file
- *Save Gene Set* – option to export to a file the list of genes currently checked

4.8 Predict

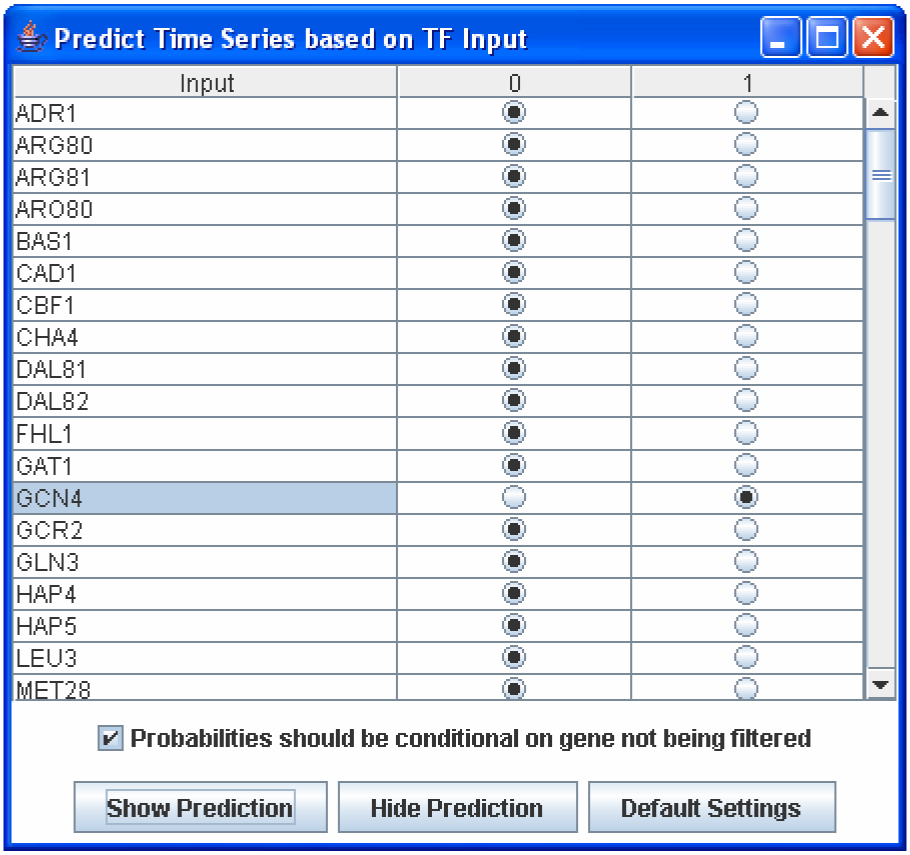

Figure 28: The window that appears after pressing the *Predict* button.

DREM allows one to view for any set of transcription factor-gene regulation interaction inputs, the probability under the model of being in each state. Figure 28 shows a dialog box in which the user is selecting to see the prediction probabilities for the input that a gene is regulated by Gcn4. After pressing the button *Show Prediction*, the probabilities appear on the main interface (see Figure 29). The predictions then appear in the node of the states. Pressing the *Hide Prediction* button hides the predictions labels. Pressing the *Default Settings* button sets all input value for each transcription to '0'. If the options *Probabilities should be conditional on gene not being filtered*, then the probabilities are computed conditional on the gene not being filtered. If the box is unchecked then all probabilities are multiplied against the probability of a gene with the selected inputs not being filtered. This probability of a gene not being filtered for a given set of inputs is determined using a Naive Bayes classifier.

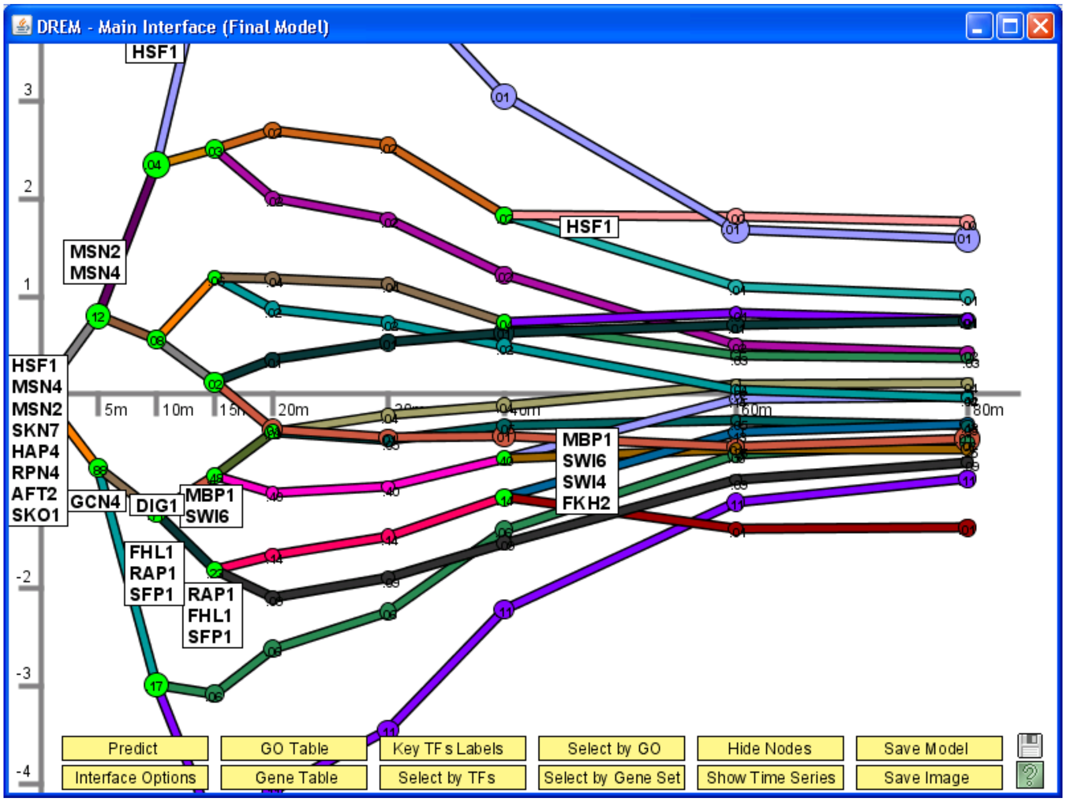

Figure 29: Map with prediction probabilities in the nodes.

4.9 Gene Table

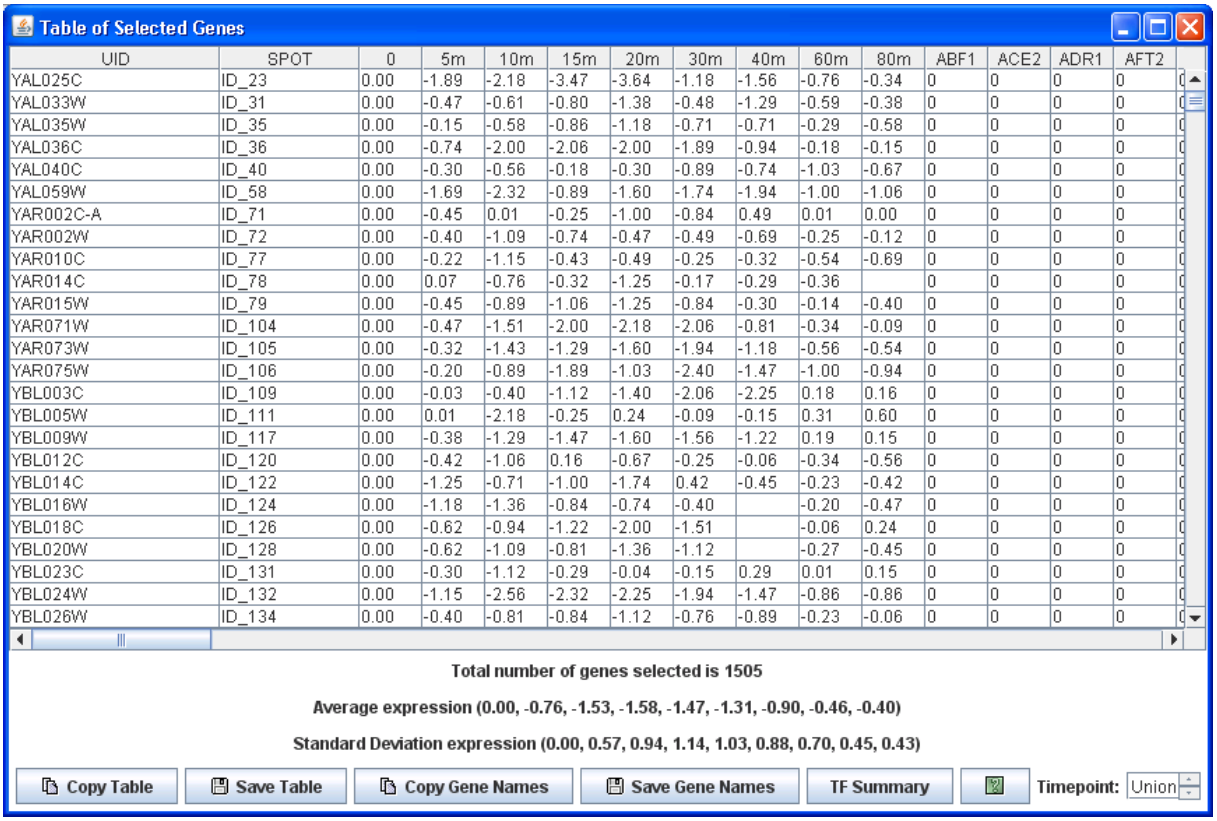

UID	SPOT	0	5m	10m	15m	20m	30m	40m	60m	80m	ABF1	ACE2	ADR1	AFT2
YAL025C	ID_23	0.00	-1.89	-2.18	-3.47	-3.64	-1.18	-1.56	-0.76	-0.34	0	0	0	0
YAL033W	ID_31	0.00	-0.47	-0.61	-0.80	-1.38	-0.48	-1.29	-0.59	-0.38	0	0	0	0
YAL035W	ID_35	0.00	-0.15	-0.58	-0.86	-1.18	-0.71	-0.71	-0.29	-0.58	0	0	0	0
YAL036C	ID_36	0.00	-0.74	-2.00	-2.06	-2.00	-1.89	-0.94	-0.18	-0.15	0	0	0	0
YAL040C	ID_40	0.00	-0.30	-0.56	-0.18	-0.30	-0.89	-0.74	-1.03	-0.67	0	0	0	0
YAL059W	ID_58	0.00	-1.69	-2.32	-0.89	-1.60	-1.74	-1.94	-1.00	-1.06	0	0	0	0
YAR002C-A	ID_71	0.00	-0.45	0.01	-0.25	-1.00	-0.84	0.49	0.01	0.00	0	0	0	0
YAR002W	ID_72	0.00	-0.40	-1.09	-0.74	-0.47	-0.49	-0.69	-0.25	-0.12	0	0	0	0
YAR010C	ID_77	0.00	-0.22	-1.15	-0.43	-0.49	-0.25	-0.32	-0.54	-0.69	0	0	0	0
YAR014C	ID_78	0.00	0.07	-0.76	-0.32	-1.25	-0.17	-0.29	-0.36		0	0	0	0
YAR015W	ID_79	0.00	-0.45	-0.89	-1.06	-1.25	-0.84	-0.30	-0.14	-0.40	0	0	0	0
YAR071W	ID_104	0.00	-0.47	-1.51	-2.00	-2.18	-2.06	-0.81	-0.34	-0.09	0	0	0	0
YAR073W	ID_105	0.00	-0.32	-1.43	-1.29	-1.60	-1.94	-1.18	-0.56	-0.54	0	0	0	0
YAR075W	ID_106	0.00	-0.20	-0.89	-1.89	-1.03	-2.40	-1.47	-1.00	-0.94	0	0	0	0
YBL003C	ID_109	0.00	-0.03	-0.40	-1.12	-1.40	-2.06	-2.25	0.18	0.16	0	0	0	0
YBL005W	ID_111	0.00	0.01	-2.18	-0.25	0.24	-0.09	-0.15	0.31	0.60	0	0	0	0
YBL009W	ID_117	0.00	-0.38	-1.29	-1.47	-1.60	-1.56	-1.22	0.19	0.15	0	0	0	0
YBL012C	ID_120	0.00	-0.42	-1.06	0.16	-0.67	-0.25	-0.06	-0.34	-0.56	0	0	0	0
YBL014C	ID_122	0.00	-1.25	-0.71	-1.00	-1.74	0.42	-0.45	-0.23	-0.42	0	0	0	0
YBL016W	ID_124	0.00	-1.18	-1.36	-0.84	-0.74	-0.40		-0.20	-0.47	0	0	0	0
YBL018C	ID_126	0.00	-0.62	-0.94	-1.22	-2.00	-1.51		-0.06	0.24	0	0	0	0
YBL020W	ID_128	0.00	-0.62	-1.09	-0.81	-1.36	-1.12		-0.27	-0.45	0	0	0	0
YBL023C	ID_131	0.00	-0.30	-1.12	-0.29	-0.04	-0.15	0.29	0.01	0.15	0	0	0	0
YBL024W	ID_132	0.00	-1.15	-2.56	-2.32	-2.25	-1.94	-1.47	-0.86	-0.86	0	0	0	0
YBL026W	ID_134	0.00	-0.40	-0.81	-0.84	-1.12	-0.76	-0.89	-0.23	-0.06	0	0	0	0

Figure 30: An example of a gene table in DREM. The table shows all genes that currently are selected.

Pressing the *Gene Table* button displays a table which has a row corresponding to every gene that is currently selected on the main output window. The table includes the gene's expression values after transformation. On the bottom of the table are the average and standard deviation of the expression values at each time point. An example of such a table is shown in Figure 30.

The columns of the table are as follows:

- *Gene Symbol* – This column contains the gene symbols. The name for this column is read from the header in the data file.
- *Spot ID* – An entry in this column contains a list of spot IDs of spots which contain the gene of the row. The entries are delimited by a ';'. The header for this column is read from the data file if the spot IDs are included in the data file.
- *Time Point columns* – The time series of gene expression levels for the gene after any selected transformation (*Log normalize data*, *Normalize data*, or *No normalization/add 0*). The header for these columns are read from the data file.
- *TF-gene columns* – These columns contain the transcription factor-gene regulation interaction inputs

This table as all tables in DREM, can be sorted by any column. Click once on a column header to sort the table in ascending order by that column's values. Click twice on the column header to sort the table in descending order, and a third time to return the table to its original order. To cycle through the sorting options in the opposite order hold down the *Shift* button when clicking. To do a compound sort on multiple columns hold down the *Ctrl* button when clicking. Also as with all tables in DREM a user can save the contents of the table by pressing the *Save Table* button. As with any gene table in DREM, a user can also just save the list of gene names using the *Save Gene Names* button. The button *Copy Table* copies the content of the table to the clipboard, while the button *Copy Gene Names* copies the gene names to the clipboard. Clicking on the button *TF Summary* displays a summary of the Transcription Factor gene interaction for the given table described below.

4.9.1 TF-Summary Table

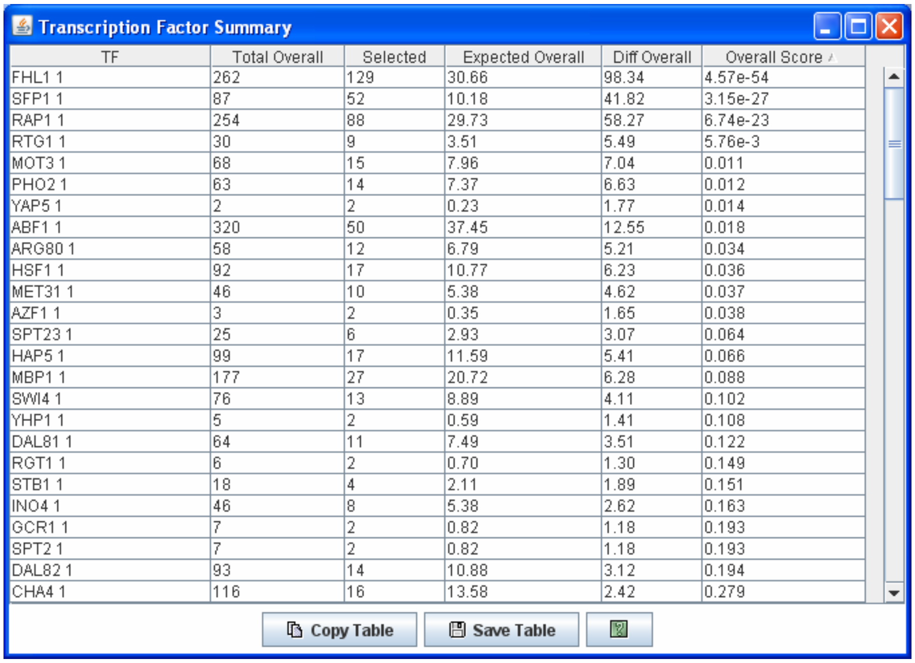

TF	Total Overall	Selected	Expected Overall	Diff Overall	Overall Score
FHL1 1	262	129	30.66	98.34	4.57e-54
SFP1 1	87	52	10.18	41.82	3.15e-27
RAP1 1	254	88	29.73	58.27	6.74e-23
RTG1 1	30	9	3.51	5.49	5.76e-3
MOT3 1	68	15	7.96	7.04	0.011
PHO2 1	63	14	7.37	6.63	0.012
YAP5 1	2	2	0.23	1.77	0.014
ABF1 1	320	50	37.45	12.55	0.018
ARG80 1	58	12	6.79	5.21	0.034
HSF1 1	92	17	10.77	6.23	0.036
MET31 1	46	10	5.38	4.62	0.037
AZF1 1	3	2	0.35	1.65	0.038
SPT23 1	25	6	2.93	3.07	0.064
HAP5 1	99	17	11.59	5.41	0.066
MBP1 1	177	27	20.72	6.28	0.088
SWI4 1	76	13	8.89	4.11	0.102
YHP1 1	5	2	0.59	1.41	0.108
DAL81 1	64	11	7.49	3.51	0.122
RGT1 1	6	2	0.70	1.30	0.149
STB1 1	18	4	2.11	1.89	0.151
INO4 1	46	8	5.38	2.62	0.163
GCR1 1	7	2	0.82	1.18	0.193
SPT2 1	7	2	0.82	1.18	0.193
DAL82 1	93	14	10.88	3.12	0.194
CHA4 1	116	16	13.58	2.42	0.279

Figure 31: Table showing aggregate information about the TF-gene regulation interactions among genes in the table.

A TF-summary table provides aggregate TF-gene interaction information for the Gene Table. The table has six columns. The columns are as follows:

- *TF* – The name of the transcription factor and the value of the annotation for the TF. Only non-zero ('1' or '-1') annotations are included.
- *Total Overall* – The number of interactions for the transcription factor of the specified value in the *TF* column among genes in the file.
- *Selected* – The number of interactions of the transcription factor of the specified value in the *TF* column among genes that were in the Gene Table.

- *Expected Overall* – The expected number of interactions of that value for a random set of genes the same size as in the Gene Table. This is the number of genes in the table times the value in *Total Overall* divided by the total number of genes in the expression data.
- *Diff Overall* – The difference between *Selected* and *Expected Overall*.
- *Overall Score* – The hypergeometric distribution probability of seeing a greater value than *Selected*. Note if the TF data was used to learn the model it does not represent a true p-value, but lower values still mean a more significant association.

4.10 GO Table

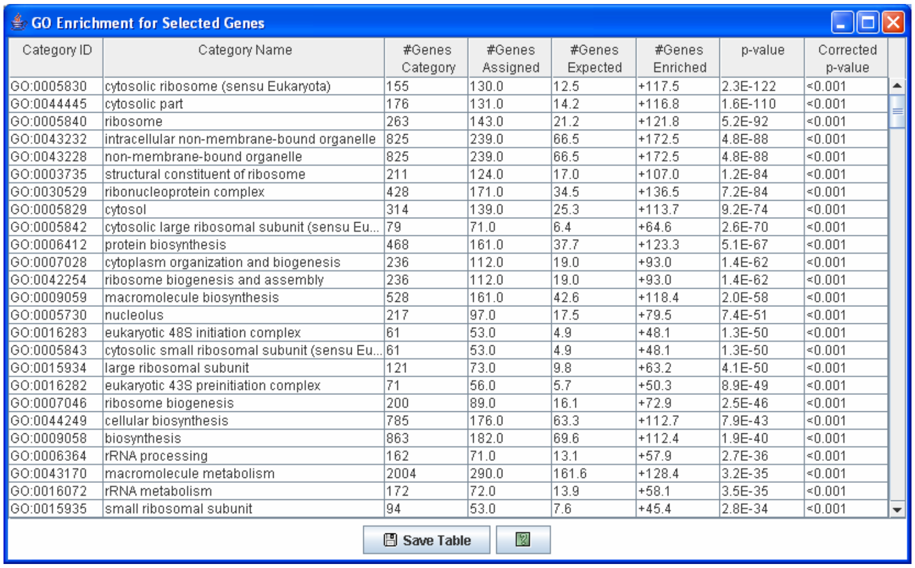

Category ID	Category Name	#Genes Category	#Genes Assigned	#Genes Expected	#Genes Enriched	p-value	Corrected p-value
GO:0005830	cytosolic ribosome (sensu Eukaryota)	155	130.0	12.5	+117.5	2.3E-122	<0.001
GO:0044445	cytosolic part	176	131.0	14.2	+116.8	1.6E-110	<0.001
GO:0005840	ribosome	263	143.0	21.2	+121.8	5.2E-92	<0.001
GO:0043232	intracellular non-membrane-bound organelle	825	239.0	66.5	+172.5	4.8E-88	<0.001
GO:0043228	non-membrane-bound organelle	825	239.0	66.5	+172.5	4.8E-88	<0.001
GO:0003735	structural constituent of ribosome	211	124.0	17.0	+107.0	1.2E-84	<0.001
GO:0030529	ribonucleoprotein complex	428	171.0	34.5	+136.5	7.2E-84	<0.001
GO:0005829	cytosol	314	139.0	25.3	+113.7	9.2E-74	<0.001
GO:0005842	cytosolic large ribosomal subunit (sensu Eu...	79	71.0	6.4	+64.6	2.6E-70	<0.001
GO:0006412	protein biosynthesis	468	161.0	37.7	+123.3	5.1E-67	<0.001
GO:0007028	cytoplasm organization and biogenesis	236	112.0	19.0	+93.0	1.4E-62	<0.001
GO:0042254	ribosome biogenesis and assembly	236	112.0	19.0	+93.0	1.4E-62	<0.001
GO:0009059	macromolecule biosynthesis	528	161.0	42.6	+118.4	2.0E-58	<0.001
GO:0005730	nucleolus	217	97.0	17.5	+79.5	7.4E-51	<0.001
GO:0016283	eukaryotic 48S initiation complex	61	53.0	4.9	+48.1	1.3E-50	<0.001
GO:0005843	cytosolic small ribosomal subunit (sensu Eu...	61	53.0	4.9	+48.1	1.3E-50	<0.001
GO:0015934	large ribosomal subunit	121	73.0	9.8	+63.2	4.1E-50	<0.001
GO:0016282	eukaryotic 43S preinitiation complex	71	56.0	5.7	+50.3	8.9E-49	<0.001
GO:0007046	ribosome biogenesis	200	89.0	16.1	+72.9	2.5E-46	<0.001
GO:0044249	cellular biosynthesis	785	176.0	63.3	+112.7	7.9E-43	<0.001
GO:0009058	biosynthesis	863	182.0	69.6	+112.4	1.9E-40	<0.001
GO:0006364	rRNA processing	162	71.0	13.1	+57.9	2.7E-36	<0.001
GO:0043170	macromolecule metabolism	2004	290.0	161.6	+128.4	3.2E-35	<0.001
GO:0016072	rRNA metabolism	172	72.0	13.9	+58.1	3.5E-35	<0.001
GO:0015935	small ribosomal subunit	94	53.0	7.6	+45.4	2.8E-34	<0.001

Figure 32: A gene enrichment analysis table. Clicking on a row of the table brings up a gene table that includes only the genes annotated as belonging to the category of the row that are also in the set being analyzed.

From the window with details about a model profile a user has the option to display a table that includes gene enrichment for Gene Ontology (GO) categories along with any other categories that may appear in an annotation file. Figure 32 shows an example of such a table. For a category to appear in the table, the number of genes in the set of genes being analyzed that belong to the category must be greater than or equal to the value of the *Minimum number of genes* parameter on the *GO Analysis* panel under *Advanced Options*. For official GO categories the level of the category must be greater than or equal to the value of the *Minimum GO level* parameter also on the *GO Analysis* panel under *Advanced Options*.

The columns of a gene enrichment table are as follows:

- *Category ID* – The ID for the category.
- *Category Name* – The name for the category.
- *# Genes Category* – The number of genes on the entire microarray that were annotated as belonging to the category.
- *# Genes Assigned* – The number of genes annotated as belonging to the category that are part of the set of genes being analyzed.
- *# Genes Expected* – The number of genes annotated as belonging to the category that were expected to be part of the set being analyzed. This value will depend on whether an actual size or expected size profile enrichment analysis is being conducted.
- *# Genes Enriched* – The difference between *# Genes Assigned* and *# Genes Expected*

- *p-value* – The uncorrected p-value of seeing this many or more genes from this category assigned to the set of genes being analyzed. Suppose there are a total of N genes on the microarray, m of the these genes are in the category of interest, v of the genes belong to the category of interest and were also assigned to the set being analyzed, and the number of gene's assigned to the profile is s_a, then the p-value of seeing v or more genes belonging to both the category of interest and assigned to the set of interest can be computed as:

$$\sum_{i=v}^{\min(m,s_a)} \frac{\binom{m}{i}\binom{N-m}{s_a-i}}{\binom{N}{s_a}}$$

- *Corrected p-value* – The p-value corrected for testing a large number of GO categories. If the enrichment is based on a set's actual size and *Randomization* is selected as the value for *Multiple hypothesis correction method for actual size based enrichment* the corrected p-value is computed based on a randomization test. If the enrichment is computed based on a set's expected size or *Bonferroni* is selected as the value for *Multiple hypothesis correction method for actual size based enrichment*, then the corrected p-value is computed based on a Bonferroni correction. See section 3.3.5 for a discussion on these two methods for correcting GO enrichment p-values.

- *Fold* (new in 1.3.7) fold enrichment that is the number of genes assigned divided by expected

A gene enrichment table can be sorted by any column in ascending or descending order by clicking on the column header. The contents of the table can also be saved to a text file using the *Save Table* button. Clicking on a row of the gene enrichment table will display a gene table that only includes genes that belong to category of the row and also the set being analyzed. For example if a user clicked on the ribosome row, a table such as that in Figure 33 will appear which contains only genes that are in the set being analyzed and were also annotated as being ribosome genes. Pressing the button *Select by this GO Category* selects the subset of genes of this GO table on the main interface and *Unapply GO Selection Constrains* removes the selection constraint.

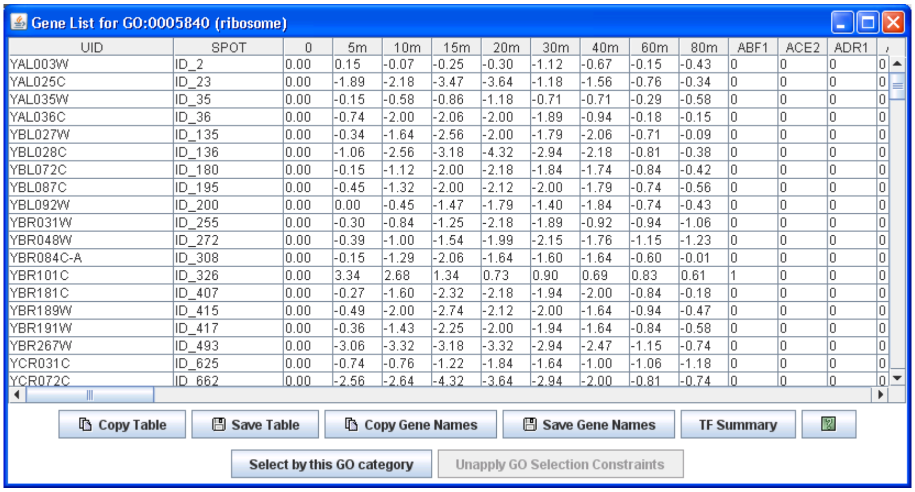
Gene List for GO:0005840 (ribosome)

UID	SPOT	0	5m	10m	15m	20m	30m	40m	60m	80m	ABF1	ACE2	ADR1	
YAL003W	ID_2	0.00	0.15	-0.07	-0.25	-0.30	-1.12	-0.67	-0.15	-0.43	0	0	0	0
YAL025C	ID_23	0.00	-1.89	-2.18	-3.47	-3.64	-1.18	-1.56	-0.76	-0.34	0	0	0	0
YAL035W	ID_35	0.00	-0.15	-0.58	-0.86	-1.18	-0.71	-0.71	-0.29	-0.58	0	0	0	0
YAL036C	ID_36	0.00	-0.74	-2.00	-2.06	-2.00	-1.89	-0.94	-0.18	-0.15	0	0	0	0
YBL027W	ID_135	0.00	-0.34	-1.64	-2.56	-2.00	-1.79	-2.06	-0.71	-0.09	0	0	0	0
YBL028C	ID_136	0.00	-1.06	-2.56	-3.18	-4.32	-2.94	-2.18	-0.81	-0.38	0	0	0	0
YBL072C	ID_180	0.00	-0.15	-1.12	-2.00	-2.18	-1.84	-1.74	-0.84	-0.42	0	0	0	0
YBL087C	ID_195	0.00	-0.45	-1.32	-2.00	-2.12	-2.00	-1.79	-0.74	-0.56	0	0	0	0
YBL092W	ID_200	0.00	0.00	-0.45	-1.47	-1.79	-1.40	-1.84	-0.74	-0.43	0	0	0	0
YBR031W	ID_255	0.00	-0.30	-0.84	-1.25	-2.18	-1.89	-0.92	-0.94	-1.06	0	0	0	0
YBR048W	ID_272	0.00	-0.39	-1.00	-1.54	-1.99	-2.15	-1.76	-1.15	-1.23	0	0	0	0
YBR084C-A	ID_308	0.00	-0.15	-1.29	-2.06	-1.64	-1.60	-1.64	-0.60	-0.01	0	0	0	0
YBR101C	ID_326	0.00	3.34	2.68	1.34	0.73	0.90	0.69	0.83	0.61	1	0	0	0
YBR181C	ID_407	0.00	-0.27	-1.60	-2.32	-2.18	-1.94	-2.00	-0.84	-0.18	0	0	0	0
YBR189W	ID_415	0.00	-0.49	-2.00	-2.74	-2.12	-2.00	-1.64	-0.94	-0.47	0	0	0	0
YBR191W	ID_417	0.00	-0.36	-1.43	-2.25	-2.00	-1.94	-1.64	-0.84	-0.58	0	0	0	0
YBR267W	ID_493	0.00	-3.06	-3.32	-3.18	-3.32	-2.94	-2.47	-1.15	-0.74	0	0	0	0
YCR031C	ID_625	0.00	-0.74	-0.76	-1.22	-1.84	-1.64	-1.00	-1.06	-1.18	0	0	0	0
YCR072C	ID_662	0.00	-2.56	-2.64	-4.32	-3.64	-2.94	-2.00	-0.81	-0.74	0	0	0	0

Copy Table Save Table Copy Gene Names Save Gene Names TF Summary

Select by this GO category Unapply GO Selection Constraints

Figure 33: A table that appears after clicking on a row in the gene enrichment table. The table only includes genes that were in the gene that were also annotated as being ribosome genes.

4.11 Save Model

Pressing the *Save Model* button opens a dialog window from which the current model can be saved into a text file. A saved model can then later be used my DREM through the *Saved Model File* field on the input to the DREM interface.

4.12 Save Image

Pressing the *Save Image* button opens a dialog box in which the main window can be saved to an image file. Note that an image can also be saved directly by using the print screen, and may be preferable. Version 1.0.9b added the ability to save the image in svg format using the Batik toolkit.

4.13 Path Table

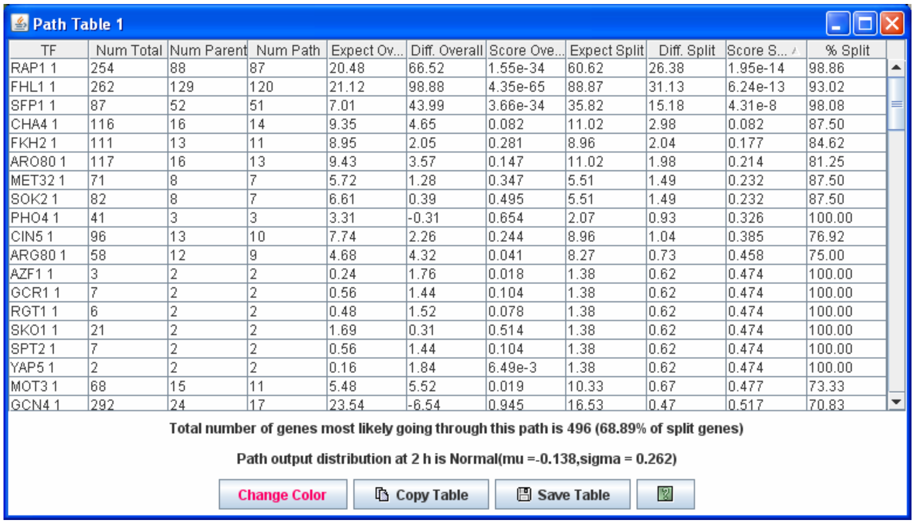
Path Table 1

TF	Num Total	Num Parent	Num Path	Expect Ov...	Diff. Overall	Score Ove...	Expect Split	Diff. Split	Score S...	% Split
RAP1 1	254	88	87	20.48	66.52	1.55e-34	60.62	26.38	1.95e-14	98.86
FHL1 1	262	129	120	21.12	98.88	4.35e-65	88.87	31.13	6.24e-13	93.02
SFP1 1	87	52	51	7.01	43.99	3.66e-34	35.82	15.18	4.31e-8	98.08
CHA4 1	116	16	14	9.35	4.65	0.082	11.02	2.98	0.082	87.50
FKH2 1	111	13	11	8.95	2.05	0.281	8.96	2.04	0.177	84.62
ARO80 1	117	16	13	9.43	3.57	0.147	11.02	1.98	0.214	81.25
MET32 1	71	8	7	5.72	1.28	0.347	5.51	1.49	0.232	87.50
SOK2 1	82	8	7	6.61	0.39	0.495	5.51	1.49	0.232	87.50
PHO4 1	41	3	3	3.31	-0.31	0.654	2.07	0.93	0.326	100.00
CIN5 1	96	13	10	7.74	2.26	0.244	8.96	1.04	0.385	76.92
ARG80 1	58	12	9	4.68	4.32	0.041	8.27	0.73	0.458	75.00
AZF1 1	3	2	2	0.24	1.76	0.018	1.38	0.62	0.474	100.00
GCR1 1	7	2	2	0.56	1.44	0.104	1.38	0.62	0.474	100.00
RGT1 1	6	2	2	0.48	1.52	0.078	1.38	0.62	0.474	100.00
SKO1 1	21	2	2	1.69	0.31	0.514	1.38	0.62	0.474	100.00
SPT2 1	7	2	2	0.56	1.44	0.104	1.38	0.62	0.474	100.00
YAP5 1	2	2	2	0.16	1.84	6.49e-3	1.38	0.62	0.474	100.00
MOT3 1	68	15	11	5.48	5.52	0.019	10.33	0.67	0.477	73.33
GCN4 1	292	24	17	23.54	-6.54	0.945	16.53	0.47	0.517	70.83

Total number of genes most likely going through this path is 496 (68.89% of split genes)

Path output distribution at 2 h is Normal(mu =-0.138,sigma = 0.262)

Change Color | Copy Table | Save Table

Figure 34: A path table with aggregate information about the regulation of genes along a path

A path table such as in Figure 34 appears when right clicking on an edge of the table. If the TF labels are based on *Path Significance Conditional on Split* or *Path Significance Overall* also right clicking on a TF labels box can bring up the table. By pressing the *Change Color* button one can change the color of the edge and genes going through the edge. The columns of the table are described below. Columns with '(Split Only)' next to them only appear when selecting edges immediately out of a split.

- *TF* – The name of the transcription factor and the value of the annotation for the TF. Only non-zero ('1' or '-1') input values are included.
- *Num Total* – The total number of genes in the expression data regulated by the transcription factor with the same input value.
- *Num Parent* (Splits only) – The number of genes going through the node immediately preceding this one on the path regulated by the transcription factor with the same input value.

- *Num Path* – The number of genes regulated by the transcription factor with the input value assigned to the path.
- *Expected Overall* – The expected number of genes assigned to the path regulated by the transcription factor with the input using all the genes in the expression data as the base set. This is computed as *Num Total* times *Num Path* divided by the number of genes in the expression data.
- *Diff. Overall* – The difference between *Num Path* and *Expected Overall*
- *Score Overall* – The hypergeometric distribution probability of seeing a greater value than *Num Path* using all genes in the expression data as the base set. Note if the TF data was used to learn the model it does not represent a true p-value, but lower values still mean a more significant association.
- *Expected Split* (Splits only) – The expected number of genes assigned to the path regulated by the transcription factor with the input value using the number of genes assigned to the parent as the base set. This is computed as *Num Total* times *Num Path* divided by *Num Parent*.
- *Diff. Split* (Splits only) – The difference between *Num Path* and *Expected Split*
- *Score Split* (Splits only) – The hypergeometric distribution probability of seeing a greater value than *Num Path* using only the genes assigned to the parent split as the base set. Note if the TF data was used to learn the model it does not represent a true p-value, but lower values still mean a more significant association.
- *Split %* (Splits only) – The percentage *Num Path* is out of *Num Parent*.

4.14 Split Table

A split table such as in Figure 35 appears when right clicking on a split node. A split node has more than one path through the node, and is green in the map. Also right clicking on a TF labels box when TF significance is determined by *Split Significance* can bring up a split table. Figure 35 is an example of a split table for a two way split with only 0-1 inputs. The fields are as follows:

- *TF* – The name of the transcription factor
- *Coeff* – The coefficient for the transcription factor in the logistic regression classifier. A positive value for the coefficient implies in the binary case that under the model a gene with a positive input value for this TF will be more likely to transition to the node with the higher mean.
- *Low 0* – The number of genes assigned to the lower path and having a '0' input for the TF.
- *Low 1* – The number of genes assigned to the lower path and having a '1' input for the TF.
- *High 0* – The number of genes assigned to the higher path and having a '0' input for the TF.
- *High 1* – The number of genes assigned to the higher path and having a '1' input for the TF.
- *Avg. Low* – The avg input value for the TF among genes assigned to the low path
- *Avg. High* – The avg input value for the TF among genes assigned to the higher path
- *Diff* – The difference between *Avg. Low* and *Avg. High*
- *Score* – The probability of having a greater absolute value of *Diff* of difference for a random assignment of the genes going through the split while holding fixed the number of genes assigned to higher and lower paths.

If '-1' inputs were included in the data file, then there would also be columns for the '-1' input values. For higher order splits, there is a table for each path out of the split. Each table makes a comparison between the genes assigned to its path with those assign to any other path out of the split.

If the path to the DECOD executable was specified (see Section 3.3.6), the *Run DECOD* button is shown in the split table. If it is a binary split node with two outgoing paths there will be two buttons *Run DECOD high* and *Run DECOD low.* High or low denotes the path from which the gene sequences are used as positive sequences in the discriminative motif search. Clicking the *Run DECOD high* button for example, will start DECOD using sequences assigned to genes in the higher path as positive sequences and use the sequences of the genes in the lower path as negatives. At higher order splits (≥ 3 paths out of a split), the currently selected tab will be used to divide genes into those on the selected path versus the genes on all other paths out of the split and therefore there is only one *Run DECOD* button.

Clicking on the *GO Split Table* displays gene enrichment analysis tables for the sets of genes for each path out of the split such as in Figure 36. The base set of genes is the set of genes going into the split. In contrast, when pressing the *GO Table* button on the main interface the base set of genes is all genes in the expression data.

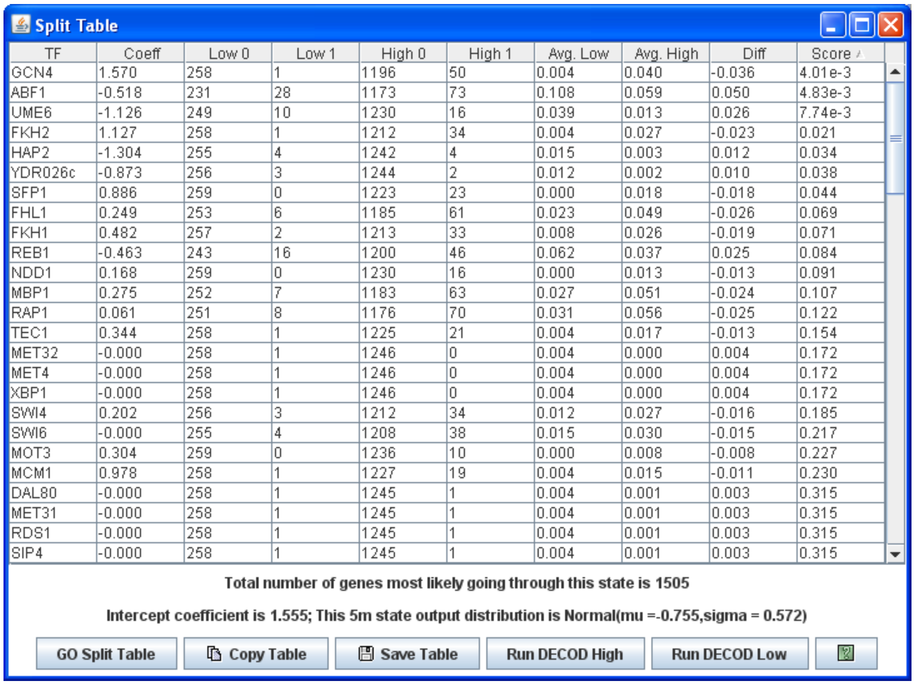

TF	Coeff	Low 0	Low 1	High 0	High 1	Avg. Low	Avg. High	Diff	Score
GCN4	1.570	258	1	1196	50	0.004	0.040	-0.036	4.01e-3
ABF1	-0.518	231	28	1173	73	0.108	0.059	0.050	4.83e-3
UME6	-1.126	249	10	1230	16	0.039	0.013	0.026	7.74e-3
FKH2	1.127	258	1	1212	34	0.004	0.027	-0.023	0.021
HAP2	-1.304	255	4	1242	4	0.015	0.003	0.012	0.034
YDR026c	-0.873	256	3	1244	2	0.012	0.002	0.010	0.038
SFP1	0.886	259	0	1223	23	0.000	0.018	-0.018	0.044
FHL1	0.249	253	6	1185	61	0.023	0.049	-0.026	0.069
FKH1	0.482	257	2	1213	33	0.008	0.026	-0.019	0.071
REB1	-0.463	243	16	1200	46	0.062	0.037	0.025	0.084
NDD1	0.168	259	0	1230	16	0.000	0.013	-0.013	0.091
MBP1	0.275	252	7	1183	63	0.027	0.051	-0.024	0.107
RAP1	0.061	251	8	1176	70	0.031	0.056	-0.025	0.122
TEC1	0.344	258	1	1225	21	0.004	0.017	-0.013	0.154
MET32	-0.000	258	1	1246	0	0.004	0.000	0.004	0.172
MET4	-0.000	258	1	1246	0	0.004	0.000	0.004	0.172
XBP1	-0.000	258	1	1246	0	0.004	0.000	0.004	0.172
SWI4	0.202	256	3	1212	34	0.012	0.027	-0.016	0.185
SWI6	-0.000	255	4	1208	38	0.015	0.030	-0.015	0.217
MOT3	0.304	259	0	1236	10	0.000	0.008	-0.008	0.227
MCM1	0.978	258	1	1227	19	0.004	0.015	-0.011	0.230
DAL80	-0.000	258	1	1245	1	0.004	0.001	0.003	0.315
MET31	-0.000	258	1	1245	1	0.004	0.001	0.003	0.315
RDS1	-0.000	258	1	1245	1	0.004	0.001	0.003	0.315
SIP4	-0.000	258	1	1245	1	0.004	0.001	0.003	0.315

Figure 35: An example of a table that appears when right clicking a split node

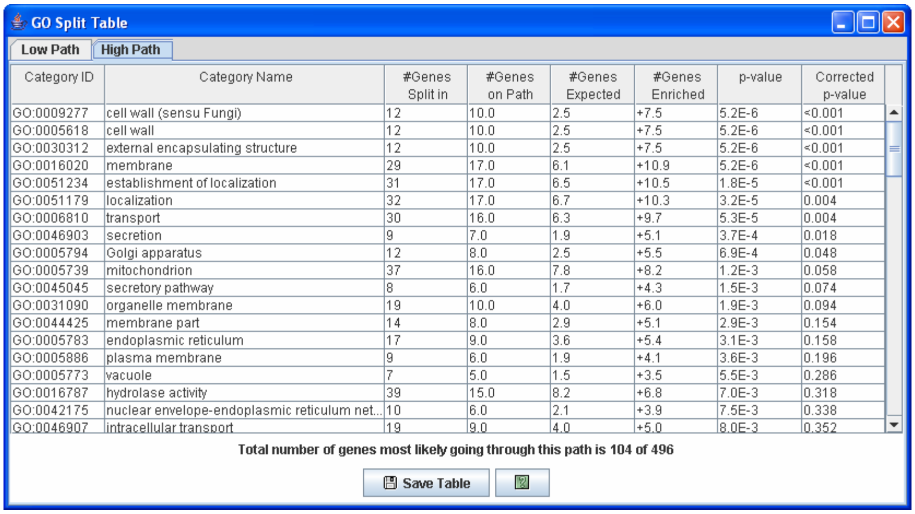

Category ID	Category Name	#Genes Split in	#Genes on Path	#Genes Expected	#Genes Enriched	p-value	Corrected p-value
GO:0009277	cell wall (sensu Fungi)	12	10.0	2.5	+7.5	5.2E-6	<0.001
GO:0005618	cell wall	12	10.0	2.5	+7.5	5.2E-6	<0.001
GO:0030312	external encapsulating structure	12	10.0	2.5	+7.5	5.2E-6	<0.001
GO:0016020	membrane	29	17.0	6.1	+10.9	5.2E-6	<0.001
GO:0051234	establishment of localization	31	17.0	6.5	+10.5	1.8E-5	<0.001
GO:0051179	localization	32	17.0	6.7	+10.3	3.2E-5	0.004
GO:0006810	transport	30	16.0	6.3	+9.7	5.3E-5	0.004
GO:0046903	secretion	9	7.0	1.9	+5.1	3.7E-4	0.018
GO:0005794	Golgi apparatus	12	8.0	2.5	+5.5	6.9E-4	0.048
GO:0005739	mitochondrion	37	16.0	7.8	+8.2	1.2E-3	0.058
GO:0045045	secretory pathway	8	6.0	1.7	+4.3	1.5E-3	0.074
GO:0031090	organelle membrane	19	10.0	4.0	+6.0	1.9E-3	0.094
GO:0044425	membrane part	14	8.0	2.9	+5.1	2.9E-3	0.154
GO:0005783	endoplasmic reticulum	17	9.0	3.6	+5.4	3.1E-3	0.158
GO:0005886	plasma membrane	9	6.0	1.9	+4.1	3.6E-3	0.196
GO:0005773	vacuole	7	5.0	1.5	+3.5	5.5E-3	0.286
GO:0016787	hydrolase activity	39	15.0	8.2	+6.8	7.0E-3	0.318
GO:0042175	nuclear envelope-endoplasmic reticulum net...	10	6.0	2.1	+3.9	7.5E-3	0.338
GO:0046907	intracellular transport	19	9.0	4.0	+5.0	8.0E-3	0.352

Figure 36: A GO table associated with a split. The enrichments are computed conditional on the set of genes going into the split.

References

[1] Bederson B. B., Grosjean J., and Meyer J. Toolkit Design for Interactive Structured Graphics. *IEEE Transactions on Software Engineering.* 30:535-546, 2004.

[2] ENCODE Project Consortium et al. Identification and analysis of functional elements in 1% of the human genome by the ENCODE pilot project. *Nature.* 447:799-816, 2007.

[3] modENCODE Consortium et al. Identification of functional elements and regulatory circuits by Drosophila modENCODE. *Science.* 330:1787–1797, 2010

[4] Ernst J. and Bar-Joseph Z. STEM: a tool for the analysis of short time series gene expression data. *BMC Bioinformatics.* 7:191, 2006.

[5] Ernst J., Beg Q.K., Kay K.A., Balázsi G., Z.N. Oltvai Z.N., Bar-Joseph Z. A Semi-Supervised Method for Predicting Transcription Factor-Gene Interactions in Escherichia coli. *PLoS Computational Biology.* 4:e1000044, 2008.

[6] Ernst J., Plasterer H.L., Simon I., Bar-Joseph Z. Integrating multiple evidence sources to predict transcription factor binding in the human genome. *Genome Research.* 20:526-536, 2010.

[7] Ernst J., Vainas O., Harbison C.T., Simon I., Bar-Joseph Z. Reconstructing dynamic regulatory maps. Nature-EMBO *Molecular Systems Biology.* 3:74, 2007.

[8] Gasch A.P., Spellman P.T., Kao C.M., Carmel-Harel O., Eisen M.B., Storz G., Botstein D., Brown P.O. Genomic expression programs in the response of yeast cells to environmental changes. *Mol. Biol. Cell.* 11, 4241-4257, 2000.

[9] The Gene Ontology Consortium. Gene Ontology: tool for the unification of biology. *Nature Genet.* 25: 25-29, 2000.

[10] Harbison CT, Gordon DB, Lee TI, Rinaldi NJ, Macisaac KD, Danford TW, Hannett NM, Tagne JB, Reynolds DB, Yoo J, Jennings EG, Zeitlinger J, Pokholok DK, Kellis M, Rolfe PA, Takusagawa KT, Lander ES, Gifford DK, Fraenkel E, Young RA Transcriptional regulatory code of a eukaryotic genome. *Nature.* 431:99-104, 2004.

[11] Huggins P., Zhong S., Shiff I., Beckerman R., Laptenko O., Prives C., Schulz M.H., Simon I., Bar-Joseph Z. DECOD: fast and accurate discriminative DNA motif finding. *Bioinformatics.* 27:2361-2367, 2011.

[12] Keseler IM, Collado-Vides J, Gama-Castro S, Ingraham J, Paley S, et al. EcoCyc: A comprehensive database resource for Escherichia coli. *Nucleic Acids Res.* 33: D334-337, 2005

[13] MacIsaac KD, Wang T, Gordon DB, Gifford DK, Stormo GD, Fraenkel E. An improved map of conserved regulatory sites for Saccharomyces cerevisiae. *BMC Bioinformatics.* 7:113, 2006.

[14] Subramanian A, Tamayo P, Mootha VK, Mukherjee S, Ebert BL, Gillette MA, Paulovich A, Pomeroy SL, Golub TR, Lander ES, Mesirov JP. Gene set enrichment analysis: a knowledge-based approach for interpreting genome-wide expression profiles. *PNAS.* 102:15545-15550, 2005.

[15] Yilmaz A, Mejia-Guerra M.K., Kurz K., Liang X., Welch L., Grotewold E. AGRIS: the Arabidopsis Gene Regulatory Information Server, an update. *Nucleic Acids Research.* 39:D1118-D1122, 2010.

A Defaults File Format

As mentioned in the preliminary section the default settings for DREM can be specified in a file and used through the *-d* on the command line. Below is a sample file. The parameters names are on the left side and a tab separates them from their value. Lines which begin with a # are comments and are ignored.

```
#Main Input:
TF-gene_Interaction_Source User Provided
TF-gene_Interactions_File input_heat.txt
Expression_Data_File expression_heat.txt
Saved_Model_File model_heat
Gene_Annotation_Source Saccharomyces cerevisiae (SGD)
Gene_Annotation_File
Cross_Reference_Source User Provided
Cross_Reference_File
Normalize_Data[Log normalize data,Normalize data,No normalization/add 0] No normalization/add 0
Spot_IDs_in_the_data_file true

#Repeat Data:
Repeat_Data_Files(comma delimited list)
Repeat_Data_is_from[Different time periods,The same time period] The same time period

#Filtering:
Filter_Gene_If_It_Has_No_Static_Input_Data false
Maximum_Number_of_Missing_Values 1
Minimum_Correlation_between_Repeats 0
Minimum_Absolute_Log_Ratio_Expression 1
Change_should_be_based_on[Maximum-Minimum,Difference From 0] Difference From 0
Pre-filtered_Gene_File

#Search Options
Allow_Path_Merges false
Maximum_number_of_paths_out_of_split 2
Use_transcription_factor-gene_interaction_data_to_build true
Saved_Model[Use As Is/Start Search From/Do Not Use] Use As Is
Convergence_Likelihood_\% 0.01

#Model Selection Options
Model_Selection_Framework[Penalized Likelihood,Train-Test] Penalized Likelihood
Penalized_Likelihood_Node_Penalty 40
Random_Seed 1260
```

```
Main_search_score_% 0
Main_search_difference_threshold 0
Delete_path_score_% 0.15
Delete_path_difference_threshold 0
Delay_split_score_% 0.15
Delay_split_difference_threshold 0
Merge_path_score_% 0.15
Merge_path_difference_threshold 0


#Gene Annotations:
Include_Biological_Process true
Include_Molecular_Function true
Include_Cellular_Process true
Only_include_annotations_with_these_evidence_codes
Only_include_annotations_with_these_taxon_IDs
Category_ID_file

#GO Analysis
Minimum_GO_level 3
Minimum_number_of_genes 5
Number_of_samples_for_randomized_multiple_hypothesis_correction 500
Multiple_hypothesis_correction_method_enrichment[Bonferroni,Randomization] Randomization

#Interface
X-axis_Scale_Factor 1
Y-axis_Scale_Factor 2
X-axis_scale[Uniform,Based on Real Time] Based on Real Time
Key_Input_X_p-val_10^-X    3
Minimum_Split_Percent 0
Scale_Node_Areas_By_The_Factor 1
Key_Input_Significance_Based_On[Path Significance Conditional on Split,
Path Significance Overall,Split Significance] Path Significance Conditional on Split
```

B TF-gene Interaction Files

Here we list the contents of the transcription factor gene interaction files included with the DREM download.

TF-gene Interaction File	Criteria for Predicted Regulation
arabidopsis_agris.txt.gz	TF-gene interactions from AtRegNet at The Arabidopsis Gene Regulatory Information Server [15]
ecoli_curated.txt	TF-gene interactions supported with curated direct experimental evidence in EcoCyc version 11.5 [12]
ecoli_predictionextended.txt	TF-gene interactions supported with curated direct experimental evidence in EcoCyc version 11.5 [12] or predicted in [5]
fly_encode.txt.gz	TF-gene interactions from a physical network by the modENCODE consortium [3]
human_encode.txt.gz	TF binding peaks within 10kb upstream or downstream of gene transcription start sites from ENCODE [2]
human_predicted_100.txt.gz	Predicted TF-gene binding interactions from [6] using the top 100 genes per PWM
human_predicted_1000.txt.gz	Predicted TF-gene binding interactions from [6] using the top 1000 genes per PWM
mouse_predicted.txt.gz	Orthology-based translation of predicted human TF-gene binding interactions from [6]
yeast_anycond005.txt.gz	Gene was bound by TF in at least one condition at a <0.005 p-value in [10]
yeast_anycond001.txt.gz	Gene was bound by TF in at least one condition at a <0.005 p-value in [10]
yeast_bindpval001_cons2.txt.gz	Regulatory Code of [13] requiring binding at a <0.001 p-value and motif conservation in at least two other yeast species
yeast_bindpval001_cons1.txt.gz	Regulatory Code of [13] requiring binding at a <0.001 p-value and motif conservation in at least one other yeast species
yeast_bindpval001_cons0.txt.gz	Regulatory Code of [13] requiring binding at a <0.001 p-value and motif presence but no conservation requirement
yeast_bindpval005_cons2.txt.gz	Regulatory Code of [13] requiring binding at a <0.005 p-value and motif conservation in at least two other yeast species
yeast_bindpval005_cons1.txt.gz	Regulatory Code of [13] requiring binding at a <0.005 p-value and motif conservation in at least one other yeast species
yeast_bindpval005_cons0.txt.gz	Regulatory Code of [13] requiring binding at a <0.005 p-value and motif presence but no conservation requirement
yeast_nobinding_cons2.txt.gz	Regulatory Code of [13] no binding requirement; motif conservation in at least two other yeast species
yeast_nobinding_cons1.txt.gz	Regulatory Code of [13] no binding requirement; motif conservation in at least one other yeast species
yeast_ypd005.txt.gz	Gene was bound by TF in YPD media at a 0.005 p-value in [10]
yeast_ypd001.txt.gz	Gene was bound by TF in YPD media at a 0.001 p-value in [10]

C Gene Annotation Sources

The table below lists all gene annotation data sets that can be selected under *Gene Annotation Source*. More information about these annotation data sets can be found here `http://www.geneontology.org/GO.current.annotations.shtml` and for the EBI annotations here `http://www.ebi.ac.uk/GOA/`. Subsets of the UniProt annotations for a large number of organisms provided by the European Bioninformatics Institute (EBI) can be found here `http://www.ebi.ac.uk/GOA/proteomes.html`, and can be used through the *User Provided* option under the *Gene Annotation Source*.

**Annotation Set**	**Source**
Anaplasma phagocytophilum HZ	J. Craig Venter Institute (JCVI)
Agrobacterium tumefaciensstr. C58	PAMGO
Arabidopsis	European Bioinformatics Institute (EBI)
Arabidopsis thaliana	The Arabidopsis Information Resource (TAIR/TIGR)
Aspergillus nidulans	AspGD
Bacillus anthracis Ames	JCVI
Caenorhabditis elegans	WormBase
Campylobacter jejuni RM1221	JCVI
Candida albicans	Candida Genome Database (CGD)
Carboxydothermus hydrogenoformans Z-2901	JCVI
Chicken	European Bioinformatics Institute (EBI)
Colwellia psychrerythraea 34H	JCVI
Cow	European Bioinformatics Institute (EBI)
Coxiella burnetii RSA 493	JCVI
Danio rerio	The Zebrafish Information Network (ZFIN)
Dehalococcoides ethenogenes 195	JCVI
Dictyostelium discoideum	DictyBase
Dickeya dadantii	PAMGO
Drosophila melanogaster	FlyBase
Ehrlichia chaffeensis Arkansas	JCVI
Escherichia coli	EcoCyc & EcoliHub
Geobacter sulfurreducens PCA	JCVI
Human	European Bioinformatics Institute (EBI)
Hyphomonas neptunium ATCC 15444	JCVI
Leishmania major	Sanger GeneDB
Listeria monocytogenes 4b F2365	JCVI
Magnaporthe grisea	PAMGO
Methylococcus capsulatus Bath	JCVI
Mouse	European Bioinformatics Institute (EBI)
Mus musculus	Mouse Genome Informatics (MGI)
Neorickettsia sennetsu Miyayama	JCVI
OOmycetes	(PAMGO)
Oryza sativa	Gramene
PDB	European Bioinformatics Institute (EBI)
Plasmodium falciparum	Sanger GeneDB
Pseudomonas aeruginosa PA01	PseduoCap
Pseudomonas fluorescens Pf-5	TIGR
Pseudomonas syringae DC3000	JCVI
Pseudomonas syringae pv. phaseolicola 1448A	TIGR
Rat	European Bioinformatics Institute (EBI)
Rattus norvegicus	Rat Genome Database (RGD)
Reactome	CSHL&EBI
Saccharomyces cerevisiae	Saccharomyces Genome Database (SGD)
Schizosaccharomyces pombe	Sanger GeneDB
Shewanella oneidensis MR-1	JCVI
Silicibacter pomeroyi DSS-3	JCVI
Solanaceae	SGN
Trypanosoma brucei	Sanger GeneDB
UniProt	European Bioinformatics Institute (EBI)
UniProt no IEA	European Bioinformatics Institute (EBI)
Vibrio cholerae	JCVI
Zebrafish	European Bioinformatics Institute (EBI)
