# Supplementary material for: DREM 2.0: Improved reconstruction of dynamic regulatory networks from time-series expression data
Source: BMC Syst Biol. 2012 Aug 16;6:104. doi: 10.1186/1752-0509-6-104 (PMC3464930; doi:10.1186/1752-0509-6-104)
Supplement: Additional file 2 — Supplementary Methods. Additional description for DREM 2.0 for TF expression level scaling, data collection for the protein-DNA binding data sets and the analysis with DECOD on an unannotated split node. [file 1752-0509-6-104-S2.pdf]

SUPPLEMENTARY METHODS AND RESULTS FOR DREM 2.0: IMPROVED RECONSTRUCTION OF DYNAMIC REGULATORY NETWORKS FROM TIME-SERIES EXPRESSION DATA

MARCEL H. SCHULZ [1,†,*] WILLIAM E. DEVANNY [2,†] ANTHONY GITTER [3] SHAN ZHONG [1]
JASON ERNST [4], ZIV BAR-JOSEPH [1,2,*]

[1]Ray and Stephanie Lane Center for Computational Biology, [2]Machine Learning Department,
[3]Computer Science Department, Carnegie Mellon University, 5000 Forbes Avenue Pittsburgh, PA 15213 USA
[4]Department of Biological Chemistry, University of California Los Angeles, Los Angeles, CA, 90095
†joint first authors
∗ corresponding authors

Contents

1. A logistic function-based method for using TF gene expression levels 1
2. Collecting protein-DNA interaction data sets 3
3. Assessment of TF binding prediction at split nodes with DECOD 4
References 11

1. A logistic function-based method for using TF gene expression levels

In DREM 2.0 it is possible to utilize the expression level of a transcription factor (TF) to influence the learning of the classifier in the input-output hidden Markov model (IOHMM). The idea is that TFs that are over- or under-expressed between time points should have a higher influence.

The expression ratio x of a TF between two time points is incorporated by using a modified version of the *logistic* function

$$f_w(x) = \frac{1}{1 + e^{-x \cdot w}} . \tag{1}$$

A shifted version of the logistic function, $f^*_w(x)$, reports 0 if no change in the expression ratio x is observed. Also instead of [0,1], the function outputs values in the range [-1,1] to allow for negative influence in the case of under expression.

$$f^*_w(x) = sign(x) \cdot \left(\frac{2}{1 + e^{-x \cdot w}} - 1 \right) , \tag{2}$$

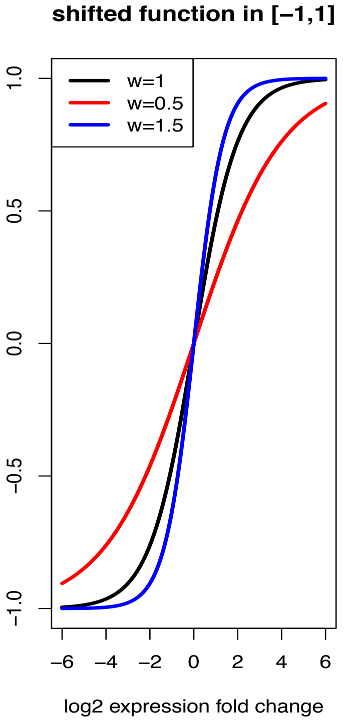

FIGURE 1. Illustration of the shifted version of the logistic function used by DREM 2.0 for scaling expression levels used in classifier learning. Output of the shifted logistic function in Eq. (2) for different weight w.

where *sign(x)* denotes the sign of the real-valued expression ratio. We call w in Eq. (2) the *expression scaling weight*, which controls the steepness of the function, see Fig. 2. TFs can be efficient at low expression levels or activated post-transcriptionally, therefore the user can define a minimum threshold, *minExpTF*. *select(x)* denotes the minimum of both according to the following formula:

$$select(x) = \begin{cases} f_w^*(x), & \text{if } abs(f_w^*(x)) \geq \text{ minExpTF}, \\ \text{sign(x)} \cdot \text{minExpTF}, & \text{else} \end{cases}, \tag{3}$$

where $abs(z)$ denotes the absolute value of a real valued number z.

The function *select(x)* is used in the following way. In the previous version of DREM a classifier could use pairwise binding information for a TF t to a gene g, denoted $\mathcal{B}_g$, with $\mathcal{B}_g \in \{-1, 1, 0\}$ representing a repressive, an activatory or no regulatory role for TF t on gene g, respectively. With expression scaling activated in DREM 2.0 we define the quantity

$\mathcal{B}'_g$:

$$\mathcal{B}'_g = select(x_t) \cdot \mathcal{B}_g\,, \tag{4}$$

where x_t denotes the gene expression ratio of the TF t and $\mathcal{B}'_g \in [-1, 1]$. For the TF t we replace all values $\mathcal{B}_g$ with $\mathcal{B}'_g$ before learning the IOHMM. This corresponds to scaling the binding value of TF t on gene g according to the logistic function $f^*_w(x_t)$.

2. Collecting protein-DNA interaction data sets

For *D. Melanogaster* we used the physical network data from the modENCODE consortium [6]. 158,558 predicted protein-DNA interactions were formatted for DREM 3-column format (the format is explained in Additional file 2).

We extracted 11,355 static protein-DNA interactions for *A. thaliana* from the AtRegNet database [7]. All protein-DNA interactions were formatted for DREM 3-column format.

Human ChIP-Seq dataset from ENCODE [2] was downloaded from the "Txn Factor ChIP" track in UCSC Genome Browser at `http://hgdownload-test.cse.ucsc.edu/goldenPath/hg19/encodeDCC/wgEncodeRegTfbsClustered/` on Oct 7, 2011. The track contains aggregated binding peaks that were computed using a uniform pipeline for 148 human transcription factors across diverse cell lines. We mapped the TF names to standard human gene names using the "Target Link" column of the ENCODE control vocabulary table at `http://genome.ucsc.edu/cgi-bin/hgEncodeVocab?ra=encode/cv.ra&type=Antibody`. In addition, ChIP-Seq peaks of the same TF in different cell lines were merged. In this way, 126 unique TFs were obtained, including some general TFs like POL2. For each human gene, we looked at the upstream 10kb and downstream 10kb window flanking the transcription start site. All TFs that have binding peaks within that window were considered to regulate that gene. In this way, 954,378 static protein-DNA interactions were obtained for human. Please consult the ENCODE data release policy (`http://genome.ucsc.edu/ENCODE/terms.html`) if these interactions are used.

Ranked human PWM-gene predictions were obtained from Ernst *et al* [3]. Each PWM was mapped to the set of corresponding TFs using TRANSFAC [5] and JASPAR [9], and each TF name was mapped to the Entrez Gene id of the gene that encodes it. Genes and TFs that could not be mapped to an Entrez Gene id were removed. This translation yielded 348 unique Entrez Gene ids from the 512 original PWMs. A protein-DNA interaction was written if any of the rows for the gene were in the top 100 predictions in any of the PWM columns matching the TF. This threshold resulted in a total of 59,578 interactions. An expanded set of 514,925 interactions was also generated by relaxing the threshold to the top 1000 predictions per PWM. In both cases interactions were formatted for DREM 3-column format.

Predicted mouse protein-DNA binding interactions were derived from the set of human predictions above that used the top 1000 threshold. Human Entrez Gene ids were translated to orthologous mouse Entrez Gene ids using the Mouse Genome Database (MGD) [1] to map identifiers and the HUGO Gene Nomenclature Committee (HGNC) database [8] for any genes MGD could not map. HGNC associated some human ids with dozens of orthologous mouse ids, thus any human id that mapped to more than 5 mouse ids was

discarded. If a human gene mapped to 2-5 mouse genes, its TF associations were transferred to all of the matching mouse genes. The translated predictions contain 468,319 protein-DNA binding interactions and were formatted for DREM 3-column format.

3. Assessment of TF binding prediction at split nodes with DECOD

In order to test the capabilities of running DECOD on DREM splits, when no TF-gene interaction data is available for a species, we conducted the following experiment. We ran DREM using only the Asbestos human data from the paper. That gave a model that was learnt without TF-gene interaction data. Using the main split at the 6 hour time point, we first computed the list of high scoring TFs using the human TF-gene interaction data set used in the paper as an annotation source (not for learning). That resulted in 36 TFs (enrichment p-value $\leq$ 9E-03) that we grouped into 24 families in table 1. Further, we used DECOD to predict binding motifs in the 871 promoter sequences from the genes in the up regulated path, contrasting 620 promoter sequences in the down regulated paths. DECOD was run with motif width 6-8 and 10 motifs were retrieved for each width. Then STAMP [4] was used to match each motif to known TF matrices in TRANSFAC (version 11.3). All hits with a STAMP E-value $\leq$ 1.5E-03 were discarded, the remaining hits are shown in table 2. Out of the 24 identified TF families DECOD was able to predict 10 (42%) as shown in table 1. We show all the DECOD identified motifs that resemble the real motifs in Fig. 2

TABLE 1. Analysis of identified transcription factors (TFs) at the 6 hour time point split node. Column 1 contains in each row TF family members identified by DREM using the human TF-gene interaction data for annotation (not learning). TFs with alternative name are shown in brackets. Column 2 shows for which of these TFs at least one family member was identified using DECOD for motif finding and subsequent matching with STAMP, see text.

TFs	identified by DECOD
VDR	
SP1,SP2,SP3	
RUNX1,RUNX2	✓
ZEB1 (AREB6)	✓
RFX1,RFX5,RFXAP	
JUN	
HNF4A	✓
SMAD1 - SMAD7	
MYB	✓
SREBF1	✓
ELK1	✓
RXRA	✓
ZNF354C	
GATA3	✓
TP63 (p53)	✓
DBP	
ZBTB7A	
BACH2	
GABPA	✓
STAT6	✓
MZF1	
RXRB	
WT1	
ZIC2	

Table 2: Full list of significant STAMP matches for the DECOD motifs. DECOD was run with motif width 6-8 (column 1) and 10 motifs were retrieved for each width (column 2). Then STAMP [4] was used to match each motif to known TF matrices in TRANSFAC (version 11.3). All hits with an E-value $\leq$ 1.5E-03 were discarded. The name of the matched TF with its TRANSFAC ID is shown in column 3 and the STAMP E-value in column 4.

Width	Motif#	Match	Evalue

Table 2: Full list of significant STAMP matches for the DECOD motifs. DECOD was run with motif width 6-8 (column 1) and 10 motifs were retrieved for each width (column 2). Then STAMP [4] was used to match each motif to known TF matrices in TRANSFAC (version 11.3). All hits with an E-value $\leq$ 1.5E-03 were discarded. The name of the matched TF with its TRANSFAC ID is shown in column 3 and the STAMP E-value in column 4.

Width	Motif#	Match	Evalue
6	Motif1	STE12_M00664	1.95E-06
6	Motif1	TCF-4_M00671	1.18E-05
6	Motif1	IRF_M00972	1.71E-04
6	Motif1	ICSBP_M00699	2.29E-04
6	Motif1	IRF_M00772	5.24E-04
6	Motif2	AR_M00447	5.07E-04
6	Motif2	TFE_M01029	5.81E-04
6	Motif2	p53_M00272	6.97E-04
6	Motif2	MATalpha2_M00031	1.50E-03
6	Motif2	DMRT3_M01148	1.82E-03
6	Motif3	ERF2_M01057	1.60E-06
6	Motif3	ATF4_M00514	1.57E-03
6	Motif3	repressor_M00014	1.62E-03
6	Motif3	CREB_M00039	1.99E-03
6	Motif3	E12_M00693	2.09E-03
6	Motif4	PEND_M01015	6.58E-06
6	Motif4	Rim101p_M01030	3.62E-05
6	Motif4	STE11_M01005	1.09E-04
6	Motif4	Alx-4_M00619	1.60E-04
6	Motif4	ROX1_M00728	1.70E-04
6	Motif5	Ets_M00971	1.42E-07
6	Motif5	PEA3_M00655	3.09E-07
6	Motif5	c-Ets-1_M00743	3.12E-07
6	Motif5	Elf-1_M00746	1.25E-06
6	Motif5	c-Ets-1_p54_M00032	1.97E-06
6	Motif6	OVO_M01101	2.52E-08
6	Motif6	SPF1_M00702	1.46E-04
6	Motif6	ROX1_M00728	1.70E-04
6	Motif6	RAV1_M00343	1.82E-04
6	Motif6	HP1_M00725	3.46E-04
6	Motif7	MYB.Ph3_M00219	1.10E-07
6	Motif7	AFP1_M00616	1.90E-06
6	Motif7	Lhx3_M00510	3.03E-04

Table 2: Full list of significant STAMP matches for the DECOD motifs. DECOD was run with motif width 6-8 (column 1) and 10 motifs were retrieved for each width (column 2). Then STAMP [4] was used to match each motif to known TF matrices in TRANSFAC (version 11.3). All hits with an E-value ≤ 1.5E-03 were discarded. The name of the matched TF with its TRANSFAC ID is shown in column 3 and the STAMP E-value in column 4.

Width	Motif#	Match	Evalue
6	Motif7	RORalpha2_M00157	5.33E-04
6	Motif7	BR-C_M00093	7.34E-04
6	Motif8	RBP-Jkappa_M01112	3.00E-08
6	Motif8	Ik-1_M00086	1.51E-07
6	Motif8	Su_H_M00234	1.94E-07
6	Motif8	Ik-3_M00088	2.83E-07
6	Motif8	Ik-2_M00087	9.77E-07
6	Motif9	PEND_M01015	1.08E-05
6	Motif9	Nkx2-5_M01043	1.81E-05
6	Motif9	Tel-2_M00678	3.89E-05
6	Motif9	RXR_M01152	9.83E-05
6	Motif9	PXR_M01153	1.31E-04
6	Motif10	Pax-1_M00326	3.53E-07
6	Motif10	Opaque-2_M00010	1.43E-06
6	Motif10	NIT2_M00142	7.85E-06
6	Motif10	Evi-1_M00011	1.78E-05
6	Motif10	GATA-1_M00347	4.48E-05
7	Motif1	MEF-2_M00405	5.61E-07
7	Motif1	aMEF-2_M00403	5.15E-06
7	Motif1	TATA_M00216	6.88E-06
7	Motif1	C-EBPgamma_M00622	2.18E-05
7	Motif1	TCF-4_M00671	2.34E-05
7	Motif2	Nanog_M01123	1.01E-06
7	Motif2	STAT3_M00225	2.60E-06
7	Motif2	IRF_M00772	3.39E-06
7	Motif2	STAT1_M00224	5.50E-06
7	Motif2	STAT1_M00492	6.43E-06
7	Motif3	Nrf-2_M00108	1.77E-09
7	Motif3	GABP_M00341	9.65E-09
7	Motif3	c-Ets-1_p54_M00032	9.37E-08
7	Motif3	c-Ets-1_M00743	4.63E-07
7	Motif3	E74A_M00016	9.51E-07
7	Motif4	dl_M00043	3.32E-06

Table 2: Full list of significant STAMP matches for the DECOD motifs. DECOD was run with motif width 6-8 (column 1) and 10 motifs were retrieved for each width (column 2). Then STAMP [4] was used to match each motif to known TF matrices in TRANSFAC (version 11.3). All hits with an E-value $\leq$ 1.5E-03 were discarded. The name of the matched TF with its TRANSFAC ID is shown in column 3 and the STAMP E-value in column 4.

Width	Motif#	Match	Evalue
7	Motif4	STE12_M00664	6.24E-06
7	Motif4	POU1F1_M00744	2.88E-05
7	Motif4	FOXP3_M00992	4.29E-05
7	Motif4	GT-1_M00635	4.56E-05
7	Motif5	DMRT5_M01150	1.22E-07
7	Motif5	OVO_M01101	1.56E-05
7	Motif5	SPF1_M00702	4.97E-05
7	Motif5	c-Ets-2_M00340	2.02E-04
7	Motif5	LXR_M00766	4.95E-04
7	Motif6	Elf-1_M00110	2.21E-06
7	Motif6	TATA_M00216	1.47E-05
7	Motif6	unc-86_M00689	4.91E-05
7	Motif6	E4BP4_M00045	6.02E-05
7	Motif6	SOX10_M01131	7.38E-05
7	Motif7	Nrf-1_M00652	3.77E-08
7	Motif7	GCM_M00634	3.91E-04
7	Motif7	Tax-CREB_M00115	3.92E-04
7	Motif7	GCM_M00270	2.00E-03
7	Motif7	p53_M00034	2.29E-03
7	Motif8	XFD-1_M00267	4.49E-06
7	Motif8	Freac-3_M00291	1.25E-05
7	Motif8	Croc_M00266	3.02E-05
7	Motif8	Lentiviral_M00318	8.24E-05
7	Motif8	BR-C_M00094	1.60E-04
7	Motif9	ERF2_M01057	4.36E-09
7	Motif9	repressor_M00014	1.45E-07
7	Motif9	E2F-1_M00430	1.91E-06
7	Motif9	E2F_M00918	3.41E-05
7	Motif9	E2F-1_M00939	4.21E-05
7	Motif10	dl_M00043	4.52E-06
7	Motif10	DEAF1_M01001	1.77E-05
7	Motif10	AREB6_M00415	1.82E-05
7	Motif10	STE12_M00664	3.15E-05

Table 2: Full list of significant STAMP matches for the DECOD motifs. DECOD was run with motif width 6-8 (column 1) and 10 motifs were retrieved for each width (column 2). Then STAMP [4] was used to match each motif to known TF matrices in TRANSFAC (version 11.3). All hits with an E-value ≤ 1.5E-03 were discarded. The name of the matched TF with its TRANSFAC ID is shown in column 3 and the STAMP E-value in column 4.

Width	Motif#	Match	Evalue
7	Motif10	PTF1-beta_M00657	1.21E-04
8	Motif1	DMRT3_M01148	1.65E-05
8	Motif1	ICSBP_M00699	5.57E-05
8	Motif1	Nanog_M01123	1.10E-04
8	Motif1	HNF3beta_M00131	1.11E-04
8	Motif1	IRF_M00772	1.46E-04
8	Motif2	STATx_M00223	3.18E-06
8	Motif2	Nrf-2_M00108	6.83E-06
8	Motif2	c-Ets-1_p54_M00032	2.19E-05
8	Motif2	E74A_M00016	2.23E-05
8	Motif2	STAT1_M00224	3.26E-05
8	Motif3	STE11_M01005	1.09E-05
8	Motif3	Rim101p_M01030	2.70E-05
8	Motif3	BR-C_M00094	9.59E-05
8	Motif3	POU1F1_M00744	1.13E-04
8	Motif3	STE11_M00274	1.66E-04
8	Motif4	MEF-2_M00405	1.33E-05
8	Motif4	aMEF-2_M00403	4.35E-05
8	Motif4	C-EBPgamma_M00622	3.74E-04
8	Motif4	FOXO3_M00477	3.95E-04
8	Motif4	BR-C_M00094	4.18E-04
8	Motif5	Elk-1_M00007	5.82E-10
8	Motif5	Nrf-2_M00108	3.89E-09
8	Motif5	GABP_M00341	3.85E-08
8	Motif5	c-Ets-1_p54_M00032	5.30E-08
8	Motif5	c-Ets-2_M00340	4.72E-07
8	Motif6	Gfi1b_M01058	4.27E-05
8	Motif6	WRKY_M00681	6.05E-04
8	Motif6	Pbx-1b_M00124	7.45E-04
8	Motif6	COMP1_M00057	2.02E-03
8	Motif6	IRF-1_M00747	2.09E-03
8	Motif7	Ik-1_M00086	1.33E-06
8	Motif7	RBP-Jkappa_M01112	1.26E-05

Table 2: Full list of significant STAMP matches for the DECOD motifs. DECOD was run with motif width 6-8 (column 1) and 10 motifs were retrieved for each width (column 2). Then STAMP [4] was used to match each motif to known TF matrices in TRANSFAC (version 11.3). All hits with an E-value $\leq$ 1.5E-03 were discarded. The name of the matched TF with its TRANSFAC ID is shown in column 3 and the STAMP E-value in column 4.

Width	Motif#	Match	Evalue
8	Motif7	Lyf-1_M00141	2.76E-05
8	Motif7	C-EBPbeta_M00117	6.27E-05
8	Motif7	Ik-3_M00088	1.21E-04
8	Motif8	HAC1_M00730	3.97E-05
8	Motif8	AR_M00481	7.92E-04
8	Motif8	YY1_M00069	1.52E-03
8	Motif8	LBP-1_M00644	1.80E-03
8	Motif8	HEB_M00698	3.72E-03
8	Motif9	ERF2_M01057	8.36E-08
8	Motif9	repressor_M00014	2.79E-06
8	Motif9	HAC1_M00730	1.53E-04
8	Motif9	ACAAT_M00309	2.53E-04
8	Motif9	E2F-1_M00430	3.11E-04
8	Motif10	Nrf-1_M00652	1.91E-06
8	Motif10	c-Ets-1_p54_M00032	4.40E-06
8	Motif10	GABP_M00341	7.25E-06
8	Motif10	c-Ets-1_M00743	1.13E-05
8	Motif10	c-Ets-1_M01078	1.41E-04

DECOD Run Width	DECOD Recovered Motif	Matched motif (Returned by STAMP)	Matched TF (STAMP)	Match E-value (STAMP)	P-value of TF Enrichment (DREM)
8	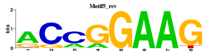	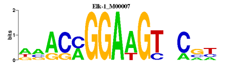	Elk-1	5.81e-10	2.18e-3 (ELK1)
7	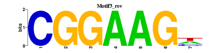	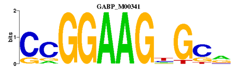	GABP	9.54e-9	7.22e-3 (GABPA)
6	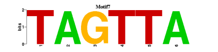	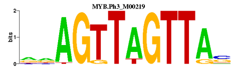	MYB.Ph3	1.10e-7	1.80e-3 (MYB)
8	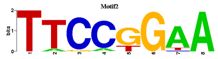	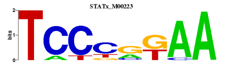	STATx	3.17e-6	7.22e-3 (STAT6)
6	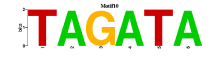	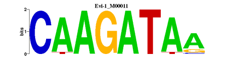	Evi-1	1.78e-5	8.74e-5 (RUNX1)
7	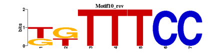	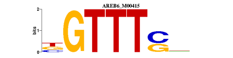	AREB6	1.83e-5	1.99e-4 (ZEB1)
6	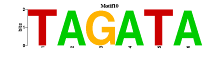	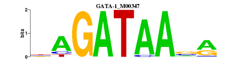	GATA-1	4.48e-5	3.17e-3 (GATA3)
6	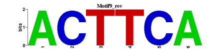	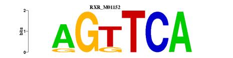	RXR	9.83e-5	2.86e-3 (RXRA)
8	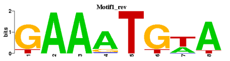	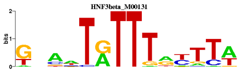	HNF3beta	1.11e-4	6.31e-4 (HNF4A)
6	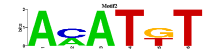	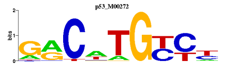	p53	6.97e-4	4.10e-3 (TP63)

FIGURE 2. Display of all DECOD predicted motifs (column 2), that are similar to one of the 24 TF family members from table 1. Column 1 gives the motif width DECOD was run with and column 3 the sequence logo of the TRANSFAC matrix that matched. The TF name (column 4), STAMP E-value (column5) and the enrichment p-value computed with DREM using the TF-gene data as annotation only (column 6) are shown.

REFERENCES

[1] Judith A Blake, Carol J Bult, James A Kadin, Joel E Richardson, Janan T Eppig, and Mouse Genome Database Group. The Mouse Genome Database (MGD): premier model organism resource for mammalian genomics and genetics. *Nucleic acids research*, 39(Database issue):D842–8, January 2011.

[2] ENCODE Project Consortium, Ewan Birney, John A Stamatoyannopoulos, Anindya Dutta, Roderic Guigó, Thomas R Gingeras, Elliott H Margulies, Zhiping Weng, Michael Snyder, Emmanouil T Dermitzakis, Robert E Thurman, Michael S Kuehn, Christopher M Taylor, Shane Neph, Christoph M Koch, Saurabh Asthana, Ankit Malhotra, Ivan Adzhubei, Jason A Greenbaum, Robert M Andrews, Paul Flicek, Patrick J Boyle, Hua Cao, Nigel P Carter, Gayle K Clelland, Sean Davis, Nathan Day,

Pawandeep Dhami, Shane C Dillon, Michael O Dorschner, Heike Fiegler, Paul G Giresi, Jeff Goldy, Michael Hawrylycz, Andrew Haydock, Richard Humbert, Keith D James, Brett E Johnson, Ericka M Johnson, Tristan T Frum, Elizabeth R Rosenzweig, Neerja Karnani, Kirsten Lee, Gregory C Lefebvre, Patrick A Navas, Fidencio Neri, Stephen C J Parker, Peter J Sabo, Richard Sandstrom, Anthony Shafer, David Vetrie, Molly Weaver, Sarah Wilcox, Man Yu, Francis S Collins, Job Dekker, Jason D Lieb, Thomas D Tullius, Gregory E Crawford, Shamil Sunyaev, William S Noble, Ian Dunham, France Denoeud, Alexandre Reymond, Philipp Kapranov, Joel Rozowsky, Deyou Zheng, Robert Castelo, Adam Frankish, Jennifer Harrow, Srinka Ghosh, Albin Sandelin, Ivo L Hofacker, Robert Baertsch, Damian Keefe, Sujit Dike, Jill Cheng, Heather A Hirsch, Edward A Sekinger, Julien Lagarde, Josep F Abril, Atif Shahab, Christoph Flamm, Claudia Fried, Jörg Hackermüller, Jana Hertel, Manja Lindemeyer, Kristin Missal, Andrea Tanzer, Stefan Washietl, Jan Korbel, Olof Emanuelsson, Jakob S Pedersen, Nancy Holroyd, Ruth Taylor, David Swarbreck, Nicholas Matthews, Mark C Dickson, Daryl J Thomas, Matthew T Weirauch, James Gilbert, Jorg Drenkow, Ian Bell, XiaoDong Zhao, K G Srinivasan, Wing-Kin Sung, Hong Sain Ooi, Kuo Ping Chiu, Sylvain Foissac, Tyler Alioto, Michael Brent, Lior Pachter, Michael L Tress, Alfonso Valencia, Siew Woh Choo, Chiou Yu Choo, Catherine Ucla, Caroline Manzano, Carine Wyss, Evelyn Cheung, Taane G Clark, James B Brown, Madhavan Ganesh, Sandeep Patel, Hari Tammana, Jacqueline Chrast, Charlotte N Henrichsen, Chikatoshi Kai, Jun Kawai, Ugrappa Nagalakshmi, Jiaqian Wu, Zheng Lian, Jin Lian, Peter Newburger, Xueqing Zhang, Peter Bickel, John S Mattick, Piero Carninci, Yoshihide Hayashizaki, Sherman Weissman, Tim Hubbard, Richard M Myers, Jane Rogers, Peter F Stadler, Todd M Lowe, Chia-Lin Wei, Yijun Ruan, Kevin Struhl, Mark Gerstein, Stylianos E Antonarakis, Yutao Fu, Eric D Green, Ulaş Karaöz, Adam Siepel, James Taylor, Laura A Liefer, Kris A Wetterstrand, Peter J Good, Elise A Feingold, Mark S Guyer, Gregory M Cooper, George Asimenos, Colin N Dewey, Minmei Hou, Sergey Nikolaev, Juan I Montoya-Burgos, Ari Löytynoja, Simon Whelan, Fabio Pardi, Tim Massingham, Haiyan Huang, Nancy R Zhang, Ian Holmes, James C Mullikin, Abel Ureta-Vidal, Benedict Paten, Michael Seringhaus, Deanna Church, Kate Rosenbloom, W James Kent, Eric A Stone, NISC Comparative Sequencing Program, Baylor College of Medicine Human Genome Sequencing Center, Washington University Genome Sequencing Center, Broad Institute, Children's Hospital Oakland Research Institute, Serafim Batzoglou, Nick Goldman, Ross C Hardison, David Haussler, Webb Miller, Arend Sidow, Nathan D Trinklein, Zhengdong D Zhang, Leah Barrera, Rhona Stuart, David C King, Adam Ameur, Stefan Enroth, Mark C Bieda, Jonghwan Kim, Akshay A Bhinge, Nan Jiang, Jun Liu, Fei Yao, Vinsensius B Vega, Charlie W H Lee, Patrick Ng, Atif Shahab, Annie Yang, Zarmik Moqtaderi, Zhou Zhu, Xiaoqin Xu, Sharon Squazzo, Matthew J Oberley, David Inman, Michael A Singer, Todd A Richmond, Kyle J Munn, Alvaro Rada-Iglesias, Ola Wallerman, Jan Komorowski, Joanna C Fowler, Phillippe Couttet, Alexander W Bruce, Oliver M Dovey, Peter D Ellis, Cordelia F Langford, David A Nix, Ghia Euskirchen, Stephen Hartman, and Al... Urban. Identification and analysis of functional elements in 1human genome by the ENCODE pilot project. *Nature*, 447(7146):799–816, June 2007.

[3] Jason Ernst, Heather L Plasterer, Itamar Simon, and Ziv Bar-Joseph. Integrating multiple evidence sources to predict transcription factor binding in the human genome. *Genome research*, 20(4):526–536, April 2010.

[4] Shaun Mahony and Panayiotis V Benos. STAMP: a web tool for exploring DNA-binding motif similarities. *Nucleic acids research*, 35(Web Server issue):W253–8, July 2007.

[5] V Matys, O V Kel-Margoulis, E Fricke, I Liebich, S Land, A Barre-Dirrie, I Reuter, D Chekmenev, M Krull, K Hornischer, N Voss, P Stegmaier, B Lewicki-Potapov, H Saxel, A E Kel, and E Wingender. TRANSFAC and its module TRANSCompel: transcriptional gene regulation in eukaryotes. *Nucleic acids research*, 34(Database issue):D108–10, January 2006.

[6] modENCODE Consortium, Sushmita Roy, Jason Ernst, Peter V Kharchenko, Pouya Kheradpour, Nicolas Negre, Matthew L Eaton, Jane M Landolin, Christopher A Bristow, Lijia Ma, Michael F Lin, Stefan Washietl, Bradley I Arshinoff, Ferhat Ay, Patrick E Meyer, Nicolas Robine, Nicole L Washington, Luisa Di Stefano, Eugene Berezikov, Christopher D Brown, Rogerio Candeias, Joseph W Carlson, Adrian

Carr, Irwin Jungreis, Daniel Marbach, Rachel Sealfon, Michael Y Tolstorukov, Sebastian Will, Artyom A Alekseyenko, Carlo Artieri, Benjamin W Booth, Angela N Brooks, Qi Dai, Carrie A Davis, Michael O Duff, Xin Feng, Andrey A Gorchakov, Tingting Gu, Jorja G Henikoff, Philipp Kapranov, Renhua Li, Heather K MacAlpine, John Malone, Aki Minoda, Jared Nordman, Katsutomo Okamura, Marc Perry, Sara K Powell, Nicole C Riddle, Akiko Sakai, Anastasia Samsonova, Jeremy E Sandler, Yuri B Schwartz, Noa Sher, Rebecca Spokony, David Sturgill, Marijke van Baren, Kenneth H Wan, Li Yang, Charles Yu, Elise Feingold, Peter Good, Mark Guyer, Rebecca Lowdon, Kami Ahmad, Justen Andrews, Bonnie Berger, Steven E Brenner, Michael R Brent, Lucy Cherbas, Sarah C R Elgin, Thomas R Gingeras, Robert Grossman, Roger A Hoskins, Thomas C Kaufman, William Kent, Mitzi I Kuroda, Terry Orr-Weaver, Norbert Perrimon, Vincenzo Pirrotta, James W Posakony, Bing Ren, Steven Russell, Peter Cherbas, Brenton R Graveley, Suzanna Lewis, Gos Micklem, Brian Oliver, Peter J Park, Susan E Celniker, Steven Henikoff, Gary H Karpen, Eric C Lai, David M MacAlpine, Lincoln D Stein, Kevin P White, and Manolis Kellis. Identification of functional elements and regulatory circuits by Drosophila modENCODE. *Science (New York, N.Y.)*, 330(6012):1787–1797, December 2010.

[7] Saranyan K Palaniswamy, Stephen James, Hao Sun, Rebecca S Lamb, Ramana V Davuluri, and Erich Grotewold. AGRIS and AtRegNet. a platform to link cis-regulatory elements and transcription factors into regulatory networks. *Plant physiology*, 140(3):818–829, March 2006.

[8] Ruth L Seal, Susan M Gordon, Michael J Lush, Mathew W Wright, and Elspeth A Bruford. genenames.org: the HGNC resources in 2011. *Nucleic acids research*, 39(Database issue):D514–9, January 2011.

[9] Dominique Vlieghe, Albin Sandelin, Pieter J De Bleser, Kris Vleminckx, Wyeth W Wasserman, Frans van Roy, and Boris Lenhard. A new generation of JASPAR, the open-access repository for transcription factor binding site profiles. *Nucleic acids research*, 34(Database issue):D95–7, January 2006.
